# A mutant allele of the long-day floral activator NtFT5 is associated with the short-day-specific flowering of tobacco cultivar Maryland Mammoth

**DOI:** 10.1038/s42003-026-10729-4

**Published:** 2026-08-26

**Authors:** Lena Grundmann, Marius M. Zimmermann, Christos Iordanidis, Andrea Känel, Axel Schwarze, David R. Wiedmann, Jost Muth, Richard M. Twyman, Dirk Prüfer, Gundula A. Noll

**Affiliations:** 1 Fraunhofer Institute for Molecular Biology and Applied Ecology IME, Münster, Germany; 2 Institute of Plant Biology and Biotechnology, University of Münster (UM), Münster, Germany; 3 Fraunhofer Institute for Molecular Biology and Applied Ecology IME, Aachen, Germany; 4 TRM Ltd., Scarborough, UK

**Keywords:** Flowering, Plant molecular biology

## Abstract

Flowering in day-neutral tobacco (*Nicotiana tabacum*) cultivars requires the photoperiod-dependent expression of FLOWERING LOCUS T (FT)-like floral activators, which compete with FT-like inhibitors to interact with FD proteins and induce the switch from vegetative to reproductive growth. In the short-day (SD) cultivar Maryland Mammoth (MM), vegetative growth persists under long-day (LD) conditions, generating unusually tall plants. We found that overexpression of the major LD floral inducer (NtFT5) from the day-neutral cultivar Hicks induces flowering in MM plants under LD conditions. However, a 2-bp deletion near the end of the endogenous *NtFT5*_MM_ gene generates a truncated NtFT5_MM_ protein that still interacts with FD proteins but acts as a weaker floral inducer than NtFT5. The truncation does not impair NtFT5_MM_ protein stability but may affect its binding to NtFD1, weakening the expression of target genes. Our results provide a potential explanation for the MM gigantism phenotype first observed more than 100 years ago.

**Figure:**
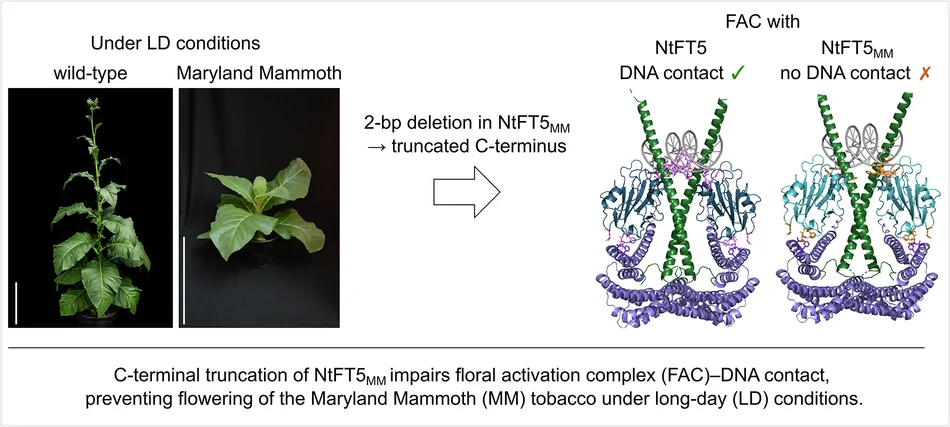

## Abstract (web summary)

A two-nucleotide deletion shortens a key floral protein, weakening its ability to activate flowering and explaining why Maryland Mammoth tobacco, central to the discovery of photoperiodism over 100 years ago, only flowers under short days.

## Introduction

The transition from vegetative to reproductive development in flowering plants requires a precisely timed response to multiple endogenous and exogenous signals. The latter often include day length, a phenomenon known as photoperiodism, and plants can be assigned to three groups on this basis: short-day (SD), long-day (LD), or day-neutral. The earliest description of photoperiodism involved the tobacco cultivar Maryland Mammoth (MM), which fails to flower under LD conditions and therefore exhibits extreme vegetative growth^1^. Subsequent work confirmed that this phenotype results from photoperiodic control rather than environmental stress^1^. Since these early studies, the regulation of photoperiodic flowering has been investigated in multiple plant species, often revealing that permissive photoperiodic conditions generate a mobile signal (florigen) that mediates the switch from vegetative to reproductive growth^2^. The FLOWERING LOCUS T (FT)-like protein family is a major part of that signal^3^. In many plants, FT proteins produced in the leaves are transported via the phloem to the shoot apical meristem (SAM), where they interact with bZIP transcription factors of the FD family to activate the floral transition^4–7^. In addition to its tightly regulated transcription^3^, FT is also subject to post-translational control, and specific mutations have been shown to alter FT protein stability or impair its transport to the SAM, demonstrating that these processes are essential for proper flowering, at least in Arabidopsis (*Arabidopsis thaliana*)^8,9^.

FT belongs to the phosphatidylethanolamine-binding protein (PEBP) family of widely conserved developmental regulators^10–13^. FT proteins are generally floral activators, whereas PEBPs from the TERMINAL FLOWER 1 (TFL1)-like clade are floral inhibitors that maintain the inflorescence meristem^11,14^. Interestingly, the *FT* gene family in some plants has undergone expansion and neofunctionalization, with some members evolving into inhibitors^2,15,16^. For example, two antagonistic FT homologs have been identified in sugar beet (*Beta vulgaris*), where the floral inhibitor BvFT1 represses the activator *BvFT2* at the transcriptional level^15^. In day-neutral tomato (*Solanum lycopersicum*), flowering is regulated by the differential photoperiodic expression of four *FT*-like genes encoding the floral inhibitors SlSP5G, SlSP5G2 and SlSP5G3, and the activator SlSP3D. The *SlSP3D* gene is expressed similarly under LD and SD conditions, whereas *SlSP5G* is mainly expressed under LD conditions, and *SlSP5G2* and *SlSP5G3* are mainly expressed under SD conditions^17–19^.

The tetraploid tobacco (*Nicotiana tabacum*) genome also encodes multiple FT homologs, which have been studied in detail in the day-neutral cultivar SR1^16,20,21^. Some FT homologs (NtFT1–NtFT3) are floral inhibitors whereas others (NtFT4 and NtFT5) are floral activators^16,20,21^. *NtFT1–NtFT4* are expressed predominantly under SD conditions, whereas *NtFT5* is expressed under both SD and LD conditions. Accordingly, even in day-neutral tobacco, flowering depends on the differential photoperiodic expression of these *FT* genes^16,20^. Unlike *FT* genes from other species, none of the tobacco homologs show a circadian expression profile^20^. Silencing the floral activator gene *NtFT5* by RNA interference (RNAi) significantly delayed flowering under LD conditions, whereas CRISPR/Cas9-mediated knockout of *NtFT5* rendered the mutants completely unable to flower under LD conditions. This indicates that NtFT5 is a major floral inducer during long days^20,22^. Furthermore, the *NtFT4* and *NtFT2* genes (encoding an activator and inhibitor, respectively) are expressed at similar levels under SD conditions, and the proteins show dose-dependent effects on flowering. This indicates that they compete at the protein level rather than regulating each other transcriptionally, as described for the antagonistic FT genes in sugar beet^15,20^. FT proteins assemble into heteromeric complexes with FD transcription factors, forming the so-called floral activation complex (FAC). In tobacco, three functional FD homologs have been identified (NtFD1, NtFD3 and NtFD4), all of which interact with FT proteins^20^. Importantly, NtFD1 preferentially interacts with the floral activator NtFT4 rather than the inhibitor NtFT2, thereby supporting the theory that different NtFT proteins compete for FD binding, thus fine-tuning floral transition^20^.

Based on our previous findings that NtFT5 functions as the predominant LD floral activator in tobacco^20,22^, we isolated and characterized this gene in the SD cultivar MM. We carried out overexpression studies to determine its contribution to the flowering phenotype in MM compared to the day-neutral cultivar Hicks (which is considered isogenic to MM except at the MM locus, whose genetic and molecular identity is still unknown^23^). Our results provide insight into the molecular basis of a phenomenon that has been observed but not understood for more than a century^24^.

## Results

### Expression of endogenous floral activator genes *NtFT4* and *NtFT5* in cultivars MM and Hicks

**Fig. 1:**
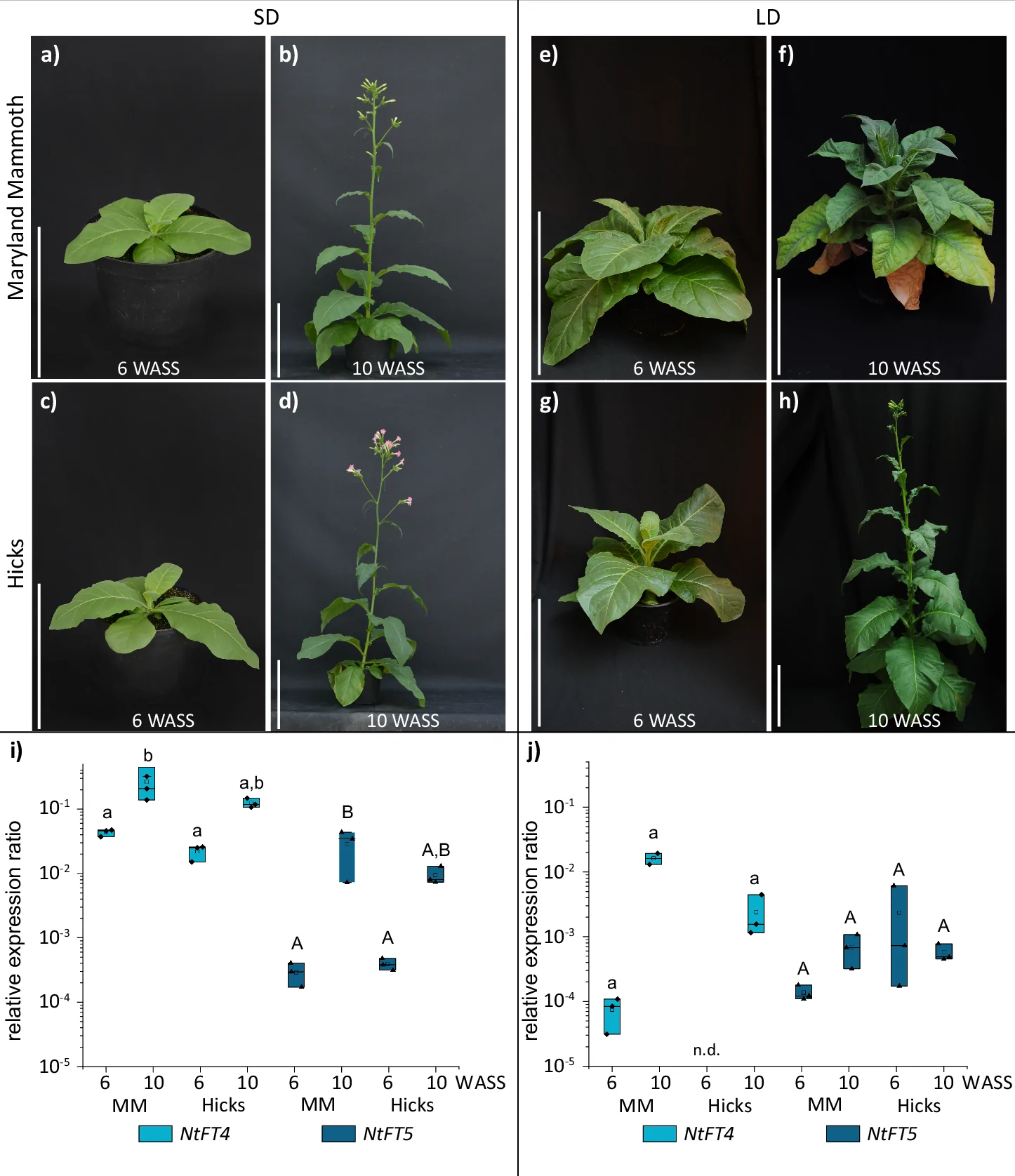
Phenotype and *NtFT4*/*NtFT5* gene expression in tobacco cultivars Maryland Mammoth (MM) and Hicks under short-day (SD) and long-day (LD) conditions. Under SD conditions, flower development was observed in (**a**, **b**) MM and (**c**, **d**) Hicks. Under LD conditions, (**e**, **f**) no flower development was observed in MM, but (**g**, **h**) flowers were produced by Hicks. Pictures were taken 6 and 10 weeks after seed sowing (WASS). Scale bars = 30 cm. *NtFT4* and *NtFT5* expression was analyzed by qPCR in *n* = 3 biological replicates per time point. Each biological replicate comprised pooled medial leaf tissue from *n* = 3 individual plants. Values were normalized to the reference gene *NtEF-1α*. In the boxplots, center line = median, open square = mean, diamonds (*NtFT4*) and rectangles (*NtFT5*) = individual data points of independent biological replicates (pooled from three individual plants), box limits = upper and lower quartiles. Different letters indicate statistically significant differences (one-way ANOVA with Bonferroni-Holm correction; *P* < 0.05). *NtFT4* was predominantly expressed under SD conditions (**i**), with mRNA levels rising during the reproductive phase in both cultivars. Under LD conditions (**j**), *NtFT4* mRNA levels were low (MM) or undetectable (Hicks) at 6 WASS and increased in both cultivars at 10 WASS, but the levels were comparable to those detected under SD conditions at 6 WASS in MM (**i**) when plants of both cultivars still showed vegetative growth (**a**, **c**). *NtFT5* expression increased with the initiation of floral development in both cultivars under SD conditions (**i**). Under LD conditions (**j**), *NtFT5* expression was slightly higher in Hicks than in MM 6 WASS, but mRNA levels were similar in both cultivars 10 WASS.

Flower development is induced under both LD and SD conditions in most tobacco cultivars, but cultivar MM flowers only under SD conditions (Fig. 1a–h). *NtFT5* knockout in tobacco results in a non-flowering phenotype under LD conditions, whereas flowering is delayed under SD conditions compared to WT plants^22^. To determine whether the LD-specific non-flowering phenotype of MM reflects the silencing of *NtFT5*, the major floral activator under LD conditions^16,20,22^, we compared the expression of *NtFT5* in MM and day-neutral Hicks by qPCR (Fig. 1i, j). We found that *NtFT5* was expressed in both cultivars under LD and SD conditions and the expression profiles were similar in each cultivar when matched for day length and the time point of harvest (6 or 10 WASS). *NtFT5* is therefore not silenced in MM. In more detail, *NtFT5* mRNA was generally less abundant under LD than SD conditions in both cultivars, and under SD conditions it was expressed at basal levels 6 WASS but was induced during the reproductive phase (10 WASS). Under LD conditions, *NtFT5* mRNA levels increased between 6 and 10 WASS in MM, whereas we detected almost no change between 6 and 10 WASS in Hicks. The increase in *NtFT5* mRNA levels between 6 and 10 WASS was less steep in MM under LD conditions than SD conditions. *NtFT5* mRNA levels were nearly identical in both cultivars 10 WASS under LD conditions, but Hicks flowered at this point whereas MM remained in the vegetative phase.

We also analyzed the expression of *NtFT4*, encoding a floral activator predominantly expressed under SD conditions in day-neutral cultivar SR1^16,20^. Our qPCR results showed that *NtFT4* was also predominantly expressed under SD conditions in Hicks and MM, and mRNA levels strongly increased during the reproductive phase. *NtFT4* mRNA was also detected at low levels under LD conditions, mainly at 10 WASS in MM, but the levels were comparable to those detected under SD conditions at 6 WASS in MM when plants of both cultivars still showed vegetative growth (Fig. 1i, j). In summary, flowering under SD conditions is strongly and significantly correlated with *NtFT4* and *NtFT5* expression for both cultivars, whereas flowering under LD conditions is only moderately and weakly correlated with *NtFT4* and *NtFT5* expression, respectively (Supplementary Data 1). Interestingly, flowering under SD conditions was delayed by 1 week in MM compared to Hicks, even though *NtFT4* and *NtFT5* were expressed at similar levels in both cultivars.

### Floral induction in transgenic MM and Hicks plants expressing *NtFT5*

**Fig. 2:**
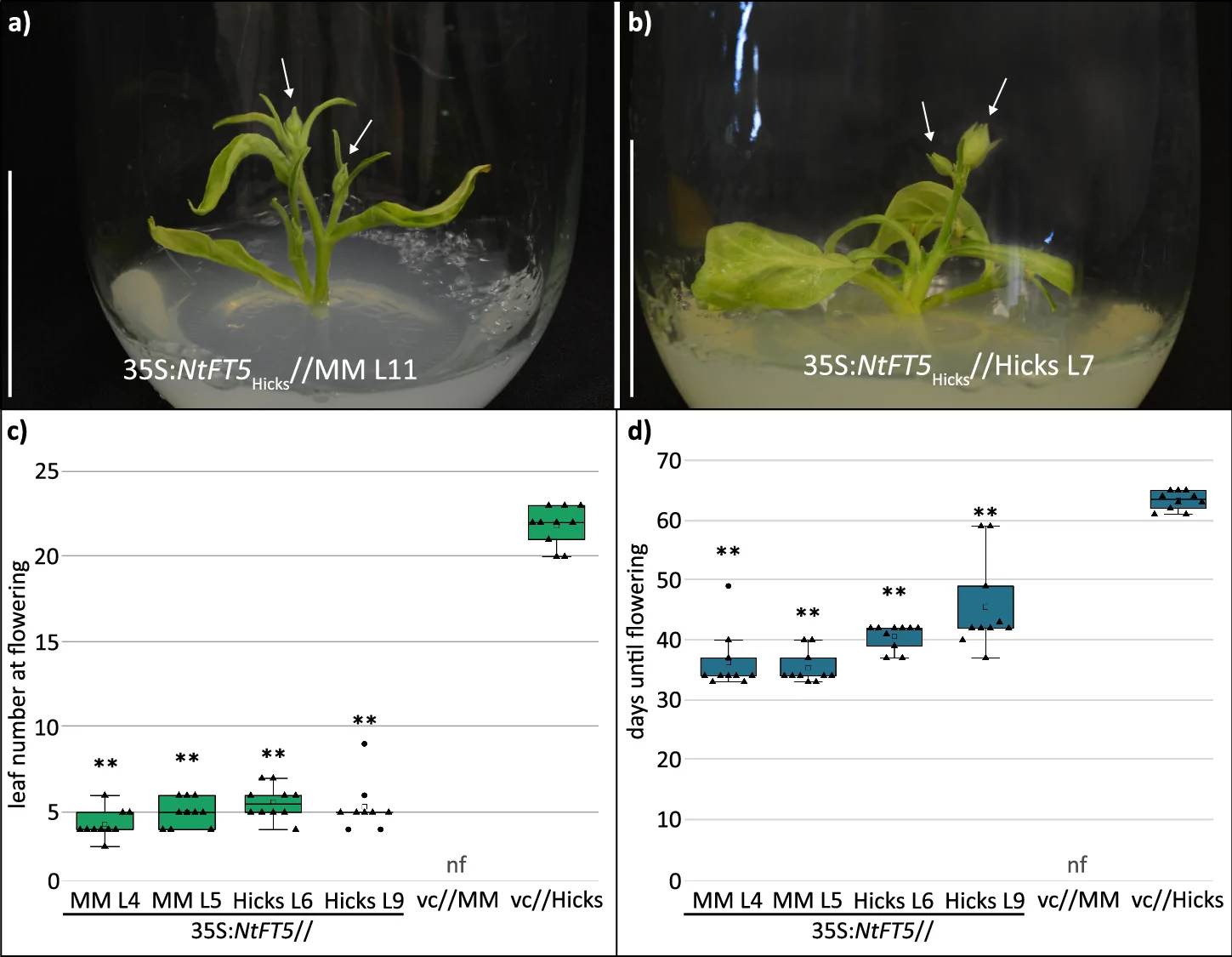
Overexpression of *NtFT5*_Hicks_ leads to early flowering in tobacco cultivars MM and Hicks. Constitutive overexpression of *NtFT5*_Hicks_ causes early flowering in (**a**) MM and (**b**) Hicks. Pictures were taken in tissue culture. Arrows indicate the formation of buds. Scale bars = 5 cm. **c** In generation T_1_, all plants were grown in the greenhouse under LD conditions. The total number of leaves was determined when the first flower had opened. All lines overexpressing *NtFT5* produced fewer leaves than the Hicks vector control. **d** The number of days until flowering was defined as the period between seed sowing and the day the first flower opened. Strong overexpression of *NtFT5* in MM and Hicks resulted in profound early flowering phenotypes compared to the Hicks and non-flowering (nf) MM vector controls under LD conditions. For (**c**, **d**), individual data points (as triangles) are shown for two 35S:*NtFT5*//MM lines (L4 and L5, n = 10), two 35S:*NtFT5*//Hicks lines (L6 *n* = 10 and L9 *n* = 9), and one vector control line for MM and Hicks (*n* = 10). Normal distribution was confirmed by applying the Kolmogorov-Smirnov test. Statistical significance was determined using a pairwise Welch’s *t*-test with Bonferroni-Holm correction (**P < 0.01). In the boxplots, center line = median, open square = mean, box limits = upper and lower quartiles, whiskers = 1.5× interquartile range, and circles = outliers.

Given that *NtFT5* is similarly expressed in both cultivars and thus not silenced in MM, we tested for a functional deficiency in the *NtFT5*_MM_ gene and whether MM has the propensity to flower under LD conditions when provided with a fully functional sequence. We isolated the *NtFT5* coding sequence from Hicks (which flowers under LD conditions) and transformed MM plants (and Hicks as a control) with the 35S:*NtFT5*_Hicks_ cassette. The constitutive expression of *NtFT5*_Hicks_ caused a profound early flowering phenotype in both cultivars (8 and 10 transgenic MM and Hicks lines, respectively; Fig. 2a, b). We found that 50% of the lines began flowering in tissue culture and failed to form roots (four 35S:*NtFT5*_Hicks_ MM lines and five 35S:*NtFT5*_Hicks_ Hicks lines), but the other 50% were transferred to the greenhouse and generated seeds. Our qPCR data revealed that transgenic lines from both cultivars accumulated ~10^4^-fold more *NtFT5* mRNA than the vector control (Supplementary Fig. S1a, b). For more detailed analysis, we studied the T_1_ progeny of two independent transgenic lines and one vector control line for each cultivar in the greenhouse under LD conditions (Fig. 2c, d). We observed profound early flowering in all transgenic lines overexpressing *NtFT5*_Hicks_, resulting in significantly fewer leaves and significantly fewer days until flowering compared to the Hicks and non-flowering MM vector control lines. *NtFT5*_Hicks_ can therefore induce flowering during the early stages of development even under LD conditions when overexpressed in the SD cultivar MM.

### *NtFT5* sequence variation and its genetic association with the MM phenotype

**Fig. 3:**
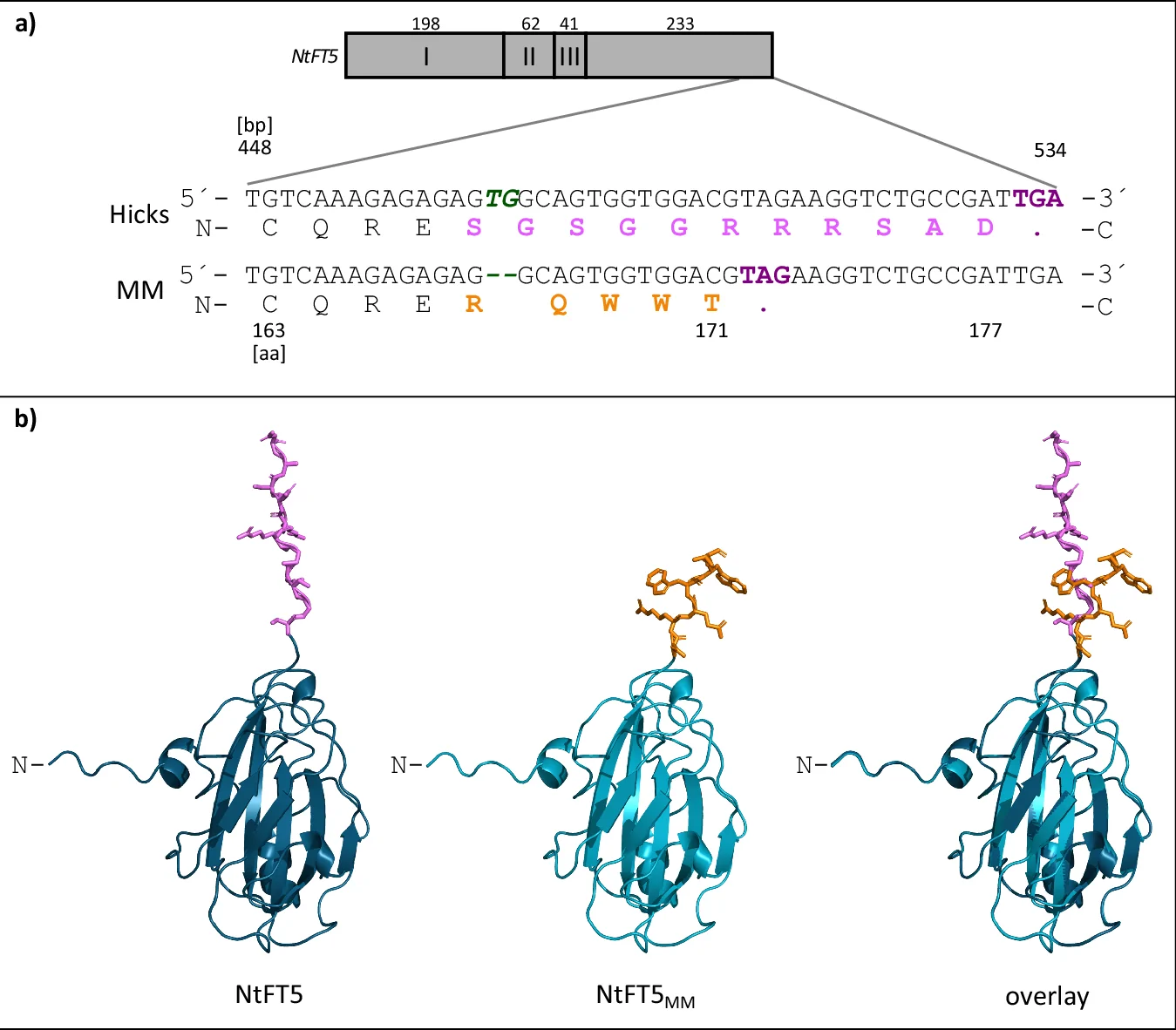
Sequence and structural analysis of NtFT5_MM_. **a** Exon structure of *NtFT5* and sequence alignment of the 3′ end of the coding sequence showing a 2-bp deletion (marked in green) 29 bp upstream of the typical stop codon (in violet) in *NtFT5*_MM_, resulting in a truncated amino acid sequence. The premature stop codon is marked in violet. **b** Structural modeling using AlphaFold^25^ likewise revealed the truncated C-terminus in NtFT5_MM_. C-terminal regions are highlighted in pink for NtFT5 (residues 167–177; SGSGGRRRSAD) and in orange for NtFT5_MM_ (residues 167–171; RQWWT).

The experiments described above showed that *NtFT5* is not suppressed in MM, but there appears to be a functional difference between the *NtFT5* genes in each cultivar. Comparing the *NtFT5*_Hicks_ and *NtFT5*_MM_ coding sequences revealed that *NtFT5*_Hicks_ is identical to *NtFT5* in cultivar SR1^18^. We therefore use the generic designation *NtFT5* hereafter when referring to the *NtFT5*_Hicks_ gene and corresponding protein. In contrast, the *NtFT5*_MM_ sequence featured a 2-bp deletion 29 bp upstream of the stop codon. The resulting frameshift mutation altered the remainder of the amino acid sequence and introduced a premature stop codon, shortening the protein by six amino acids (Fig. 3a). Structural modeling using AlphaFold^25^ likewise revealed the truncated C-terminus in NtFT5_MM_ (Fig. 3b).

**Fig. 4:**
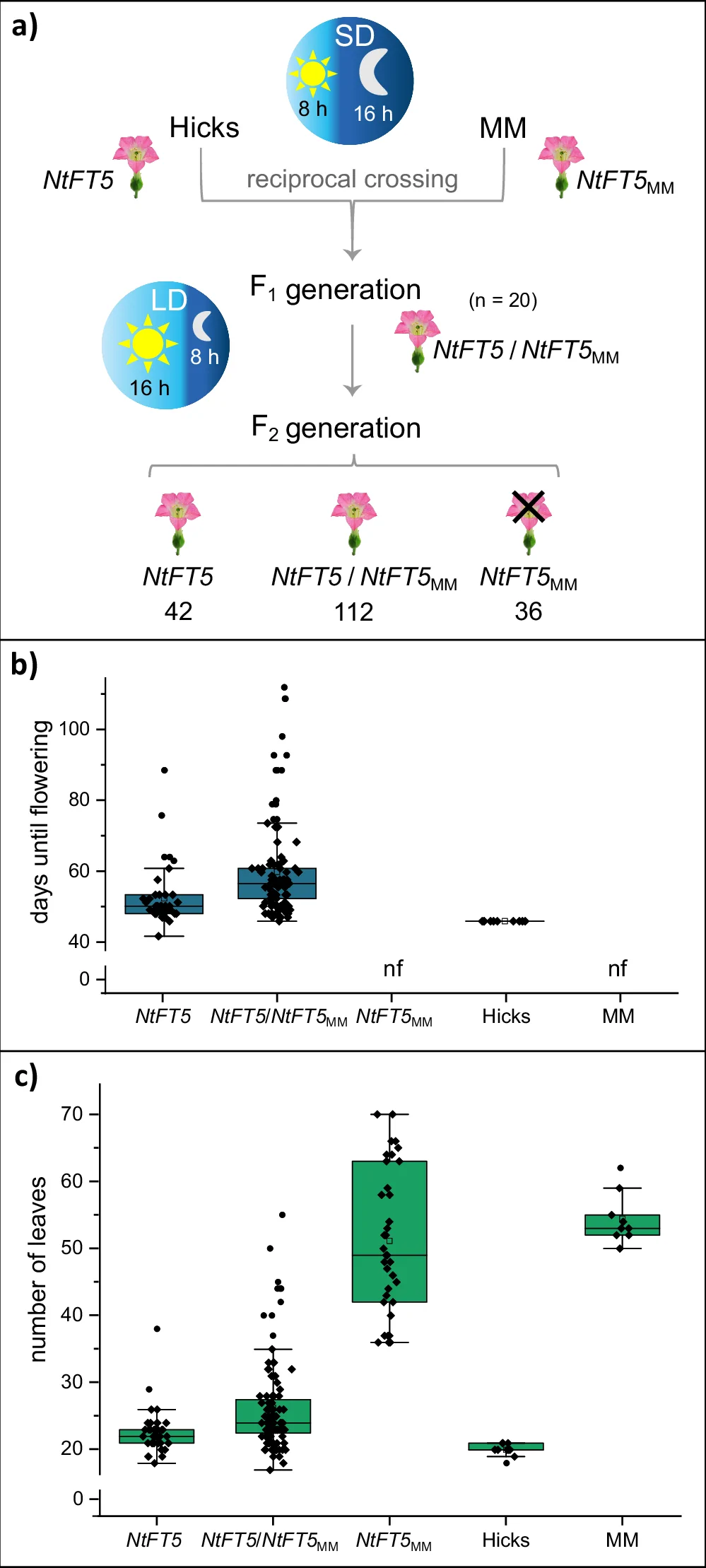
Reciprocal crosses of cultivars Hicks and MM. **a** Experimental workflow of the crossing experiment. Flowering MM and Hicks plants were crossed reciprocally. In the F_1_ generation (n = 20) all plants were heterozygous (*NtFT5*/*NtFT5*_*MM*_) and flowered. Detailed phenotyping of non-flowering homozygous *NtFT5*_*MM*_ plants and flowering *NtFT5* and *NtFT5*/*NtFT5*_*MM*_ plants was carried out in the F_2_ generation. **b** The number of days until flowering was defined as the period between seed sowing and the day the first flower opened. The presence of two functional *NtFT5* alleles in F_2_ plants (*n* = 42) conferred a normal flowering phenotype like the Hicks plants (*n* = 10), whereas heterozygosity (*NtFT5*/*NtFT5*_*MM*_) conferred a delayed flowering phenotype (*n* = 112). All F_2_ individuals homozygous for *NtFT5*_*MM*_ (*n* = 36) did not flower, like the MM plants (*n* = 9, nf = non-flowering). **c** The total number of leaves was determined when the first flower had opened (or after 120 days for non-flowering *NtFT5*_*MM*_ and MM plants at the end of the experiment). For the boxplots in (**b**, **c**), center line = median, square = mean, box limits = upper and lower quartiles, whiskers = 1.5× interquartile range, diamonds = individual data points and circles = outliers.

To determine whether the 2-bp deletion in *NtFT5*_MM_ correlates with the non-flowering phenotype, we crossed the MM and Hicks cultivars (Fig. 4a). We grew both cultivars under SD conditions to induce flowering and performed reciprocal crosses. F_1_ seeds were sown in soil, and 10 plants from each cross were grown in the greenhouse under LD conditions. All plants flowered, and FLA revealed one *NtFT5* and one *NtFT5*_MM_ allele as expected. We then pooled the seeds of 10 F_1_ plants for each reciprocal cross and cultivated 95 F_2_ plants together with the parental cultivars (*n* = 9–10) in the greenhouse under LD conditions. All Hicks plants started flowering after 46 days with a mean number of 20 leaves, whereas none of the MM plants had flowered after 120 days. In this timeframe, we observed 154 flowering and 36 non-flowering F_2_ plants (Fig. 4b; Supplementary Table S1). Genotyping the F_2_ plants revealed that all non-flowering F_2_ plants were homozygous for *NtFT5*_MM_, 112 flowering F_2_ plants were heterozygous (*NtFT5*/*NtFT5*_MM_), and 42 flowering F_2_ plants were homozygous for *NtFT5*. Interestingly, F_2_ plants homozygous for *NtFT5* flowered on average after 52 days with a mean number of 22.5 leaves, whereas heterozygous F_2_ plants flowered on average after 59 days with a mean number of 26 leaves (Fig. 4b, c). We could therefore show that the non-flowering MM phenotype under LD conditions segregates with the *NtFT5*_MM_ allele and that the presence of two functional *NtFT5* alleles induces earlier flowering compared to heterozygous plants.

### Functional comparison of NtFT5 and NtFT5_MM_ in transgenic lines

**Fig. 5:**
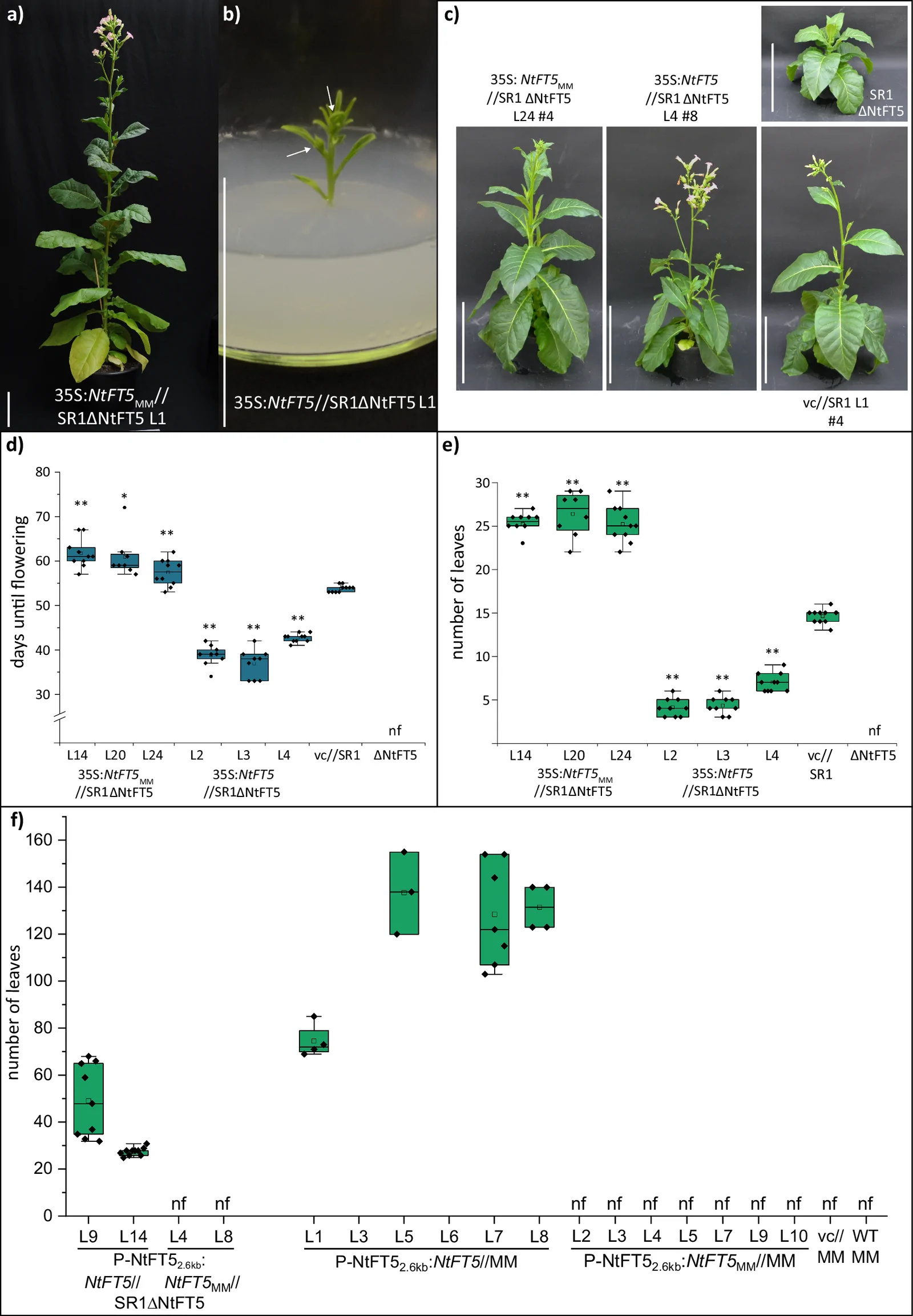
Phenotypic analysis of *NtFT5* and *NtFT5*_MM_ overexpression in SR1∆*NtFT5* plants. **a** Constitutive overexpression of *NtFT5*_MM_ in SR1∆*NtFT5* plants led to flower development in the greenhouse when the plants reached approximately 1.2 m in height. Scale bar = 10 cm. **b** In contrast, *NtFT5* overexpression in SR1∆*NtFT5* plants caused a profound early flowering phenotype in tissue culture. Arrows indicate the formation of buds. Scale bar = 10 cm. **c** Constitutive overexpression of *NtFT5*_MM_ in the non-flowering SR1∆*NtFT5* background caused delayed flowering compared to the wild-type SR1 vector control (vc) under LD conditions (T_1_ generation), indicating only partial complementation, whereas overexpression of *NtFT5* induced early flowering in transgenic plants. Scale bars = 30 cm. **d** The number of days until flowering was defined as the period between seed sowing and the day the first flower opened. Strong overexpression of *NtFT5*_MM_ in SR1∆*NtFT5* plants delayed flowering in all transgenic lines compared to the SR1 vector control. Plants overexpressing *NtFT5* showed profound early flowering phenotypes compared to the SR1 vector control. **e** The total number of leaves was determined when the first flower had opened. All SR1∆*NtFT5* lines overexpressing *NtFT5*_MM_ produced more leaves and all SR1∆*NtFT5* lines overexpressing *NtFT5* produced fewer leaves than the SR1 vector control. In (**d**, **e**), mean values are shown for three 35S:*NtFT5*_MM_//SR1∆*NtFT5* lines (L14 and L24, *n* = 10; L20, *n* = 8), three 35S:*NtFT5*//SR1∆*NtFT5* lines (L2 and L3, *n* = 9; L4, *n* = 10), and one vector control line (*n* = 10). Normal distribution was confirmed by applying the Kolmogorov-Smirnov test. Statistical significance was determined using a pairwise Welch’s *t*-test with Bonferroni-Holm correction (**P < 0.01, *P < 0.05). **f** Flowering phenotype of *SR1ΔNtFT5* plants (left) and MM plants (right) expressing *NtFT5* or *NtFT5*_*MM*_ driven by a 2.6-kb fragment of the *NtFT5* promoter. Leaf number was determined when the first flower opened. The number of flowering plants per line is provided in Supplementary Fig. S2c. For (**d**–**f**), nf = non-flowering at the end of the experiment; in the boxplots, center line = median, open square = mean, box limits = upper and lower quartiles, whiskers = 1.5× interquartile range, diamonds = individual data points and circles = outliers.

We next tested the ability of NtFT5_MM_ to act as a floral promoter. We previously used the CRISPR/Cas9 system to generate *NtFT5*-deficient SR1 plants (SR1∆*NtFT5*), which are unable to flower under LD conditions^22^. We therefore transformed these SR1∆*NtFT5* plants with constructs expressing *NtFT5* or *NtFT5*_MM_ driven by the constitutive CaMV 35S promoter (Fig. 5a, b; Supplementary Fig. S2). Most 35S:*NtFT5* transgenic plants (10/13) showed a profound early flowering phenotype that failed to produce roots and thus could not be transferred to the greenhouse for seed production. In contrast, 0/12 35S:*NtFT5*_MM_ transgenic plants flowered in tissue culture but when transferred to the greenhouse after root induction they flowered at later developmental stages (Fig. 5a). We analyzed transgene expression in young leaf tissue by qPCR (the primers amplified the same 190-bp fragment in the coding sequence of *NtFT5* and *NtFT5*_MM_) and observed higher *NtFT5*_MM_ and *NtFT5* mRNA levels in the transgenic plants than the SR1 vector control (Supplementary Fig. S2a).

Floral induction by *NtFT5*_MM_ was investigated in more detail by studying the transgenic *N. tabacum* cv. SR1∆*NtFT5* lines in the T_1_ generation (Fig. 5c–e; Supplementary Fig. S2b). We cultivated three independent transgenic lines each (*n* = 8–10) and one SR1 vector control line (n = 10) in the greenhouse under LD conditions and documented the flowering phenotype for one representative transgenic line each compared to a vector control (Fig. 5c). Although *NtFT5*_MM_ mRNA levels strongly increased compared to transgenic plants overexpressing *NtFT5* and the vector control (Supplementary Fig. S2b), *NtFT5*_MM_ overexpression did not induce early flowering in the SR1∆*NtFT5* plants as already observed with primary transformants. Flowering was significantly delayed in all three 35S:*NtFT5*_MM_//SR1∆*NtFT5* lines, and the number of leaves increased significantly compared to the SR1 vector control (Fig. 5d, e). In comparison, all individuals of the three transgenic lines overexpressing *NtFT5* showed profound early flowering, resulting in significantly fewer leaves and significantly fewer days until flowering compared to the SR1 vector control with endogenous *NtFT5* expression (Fig. 5d, e). *NtFT5*_MM_ therefore only partially complements the non-flowering phenotype of SR1∆*NtFT5* plants, showing that NtFT5_MM_ is a weaker floral inducer than NtFT5.

We also transformed SR1∆*NtFT5* and MM plants with constructs expressing *NtFT5* or *NtFT5*_MM_ driven by the ~2.6-kb endogenous *NtFT5* promoter, which is identical in SR1 and MM (Fig. 5f). None of the primary P-NtFT5_2.6kb_:*NtFT5* or P-NtFT5_2.6kb_:*NtFT5*_MM_ transgenic plants flowered in tissue culture or subsequently in the greenhouse (LD conditions). To allow seed production, these plants were grafted onto florally induced WT SR1 rootstocks. Following germination on selective media, we cultivated at least two independent transgenic lines per construct (*n* = 10), one MM vector control line and MM WT plants (*n* = 5) in the greenhouse under LD conditions. In the SR1∆*NtFT5* background, 9–10 transgenic plants per line expressing P-NtFT5_2.6kb_:*NtFT5* started flowering, but none of those expressing P-NtFT5_2.6kb_:*NtFT5*_MM_ (Fig. 5f; Supplementary Fig. S2c). When *NtFT5* was driven by the endogenous *NtFT5* promoter in the MM background, plants from four of six lines started flowering in the T_1_ generation: 3/10 from L5, 4/10 from L1 and L8, and 7/10 from L7 (Fig. 5f; Supplementary Fig. S2c). In contrast, when *NtFT5*_MM_ was driven by the endogenous *NtFT5* promoter in the MM background, none of the 70 plants (10 for each of the seven lines) flowered (Fig. 5f; Supplementary Fig. S2c). Thus, *NtFT5* but not *NtFT5*_MM_ driven by the *NtFT5* promoter could partially complement the non-flowering phenotype of SR1∆*NtFT5* and MM plants, confirming that NtFT5_MM_ is a weaker floral inducer than NtFT5. However, not all P-NtFT5_2.6kb_:*NtFT5*//MM plants flowered, so the expression of *NtFT5* driven by the 2.6-kb endogenous promoter does not fully restore native *NtFT5* expression.

### Evaluation of NtFT5_MM_ protein stability

**Fig. 6:**
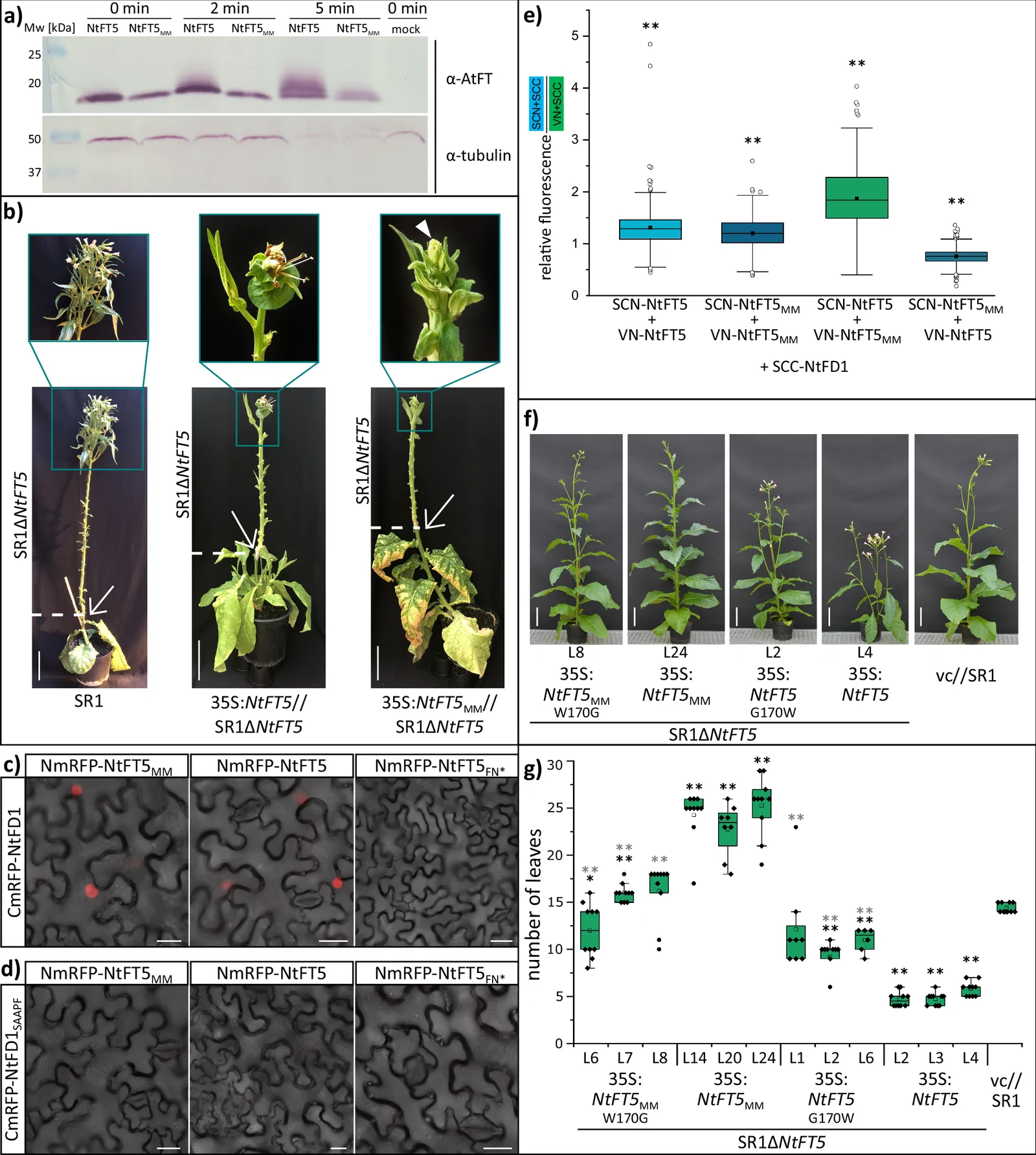
Functional characterization of NtFT5_MM_ and NtFT5. **a** Western blot to determine NtFT5 and NtFT5_MM_ protein stability in leaf samples after transient expression in *N. benthamiana* using the pTRBO vector. Leaf samples were snap-frozen and incubated at room temperature for different time intervals, and proteins were detected using AtFT- and α-tubulin-specific primary antibodies and an alkaline phosphatase-labeled secondary antibody. Precision Plus Protein standards were used as size markers (Mw). **b** Grafting experiments confirmed that NtFT5 and NtFT5_MM_ proteins are transported from wild-type (WT), 35S:*NtFT5* and 35S:*NtFT5*_MM_ stock to the SAM to induce flower-like structures on SR1∆*NtFT5* scions. Pictures were taken when scions flowered. Emerging leaves on the scion and axillary shoots on the stock were removed regularly to enhance the source–sink gradient. Scale bars = 20 cm. BiFC analysis revealed that NtFD1 (**c**) interacts with NtFT5_MM_ and NtFT5 in *N. benthamiana* leaf epidermal cells, whereas NtFD1_SAAPF_ (**d**) does not interact with tobacco FTs; 35S:*CmRFP-NtFD1* or 35S:*CmRFP-NtFD1*_SAAPF_ was co-expressed with 35S:*NmRFP* fusions of *NtFT5*_MM_, *NtFT5* and *NtFT5*_FN*_. Scale bars = 25 µm. **e** In mcBiFC assays, NtFD1 preferentially associated with NtFT5 rather than NtFT5_MM_. T-DNAs carrying two CaMV 35S cassettes encoding combinations of SCN and VN fusion proteins were introduced into *N. benthamiana* leaf epidermal cells together with a separate T-DNA containing a 35S:*SCC-NtFD1* construct. SCN + SCC and VN + SCC fluorescence was measured in the nuclei. Values represent the SCN + SCC signal divided by the VN + SCC signal. Sample sizes = individual cell nuclei from independent leaf infiltration experiments across at least three plants (left to right): 428, 341, 535, 542. SCN, N-terminal part of S(CFP)3 A; VN, N-terminal part of Venus; SCC, C-terminal part of S(CFP)3 A. Statistical significance was determined using a pairwise Welch’s t-test with Bonferroni-Holm correction (**P < 0.01). **f** Replacing NtFT5_MM_ tryptophan with glycine in transgenic 35S:*NtFT5*_MM W170G_//SR1∆*NtFT5* lines resulted in a WT-like flowering phenotype, whereas replacing glycine with tryptophan in transgenic 35S:*NtFT5*_G170W_//SR1∆*NtFT5* lines abolished the profound early flowering phenotype under LD conditions (T_1_ generation). One line each is shown as an example. Scale bars = 12 cm **g** The number of leaves was determined when the first flower opened. All SR1∆*NtFT5* lines overexpressing *NtFT5*_MM_ produced more leaves and all lines overexpressing *NtFT5* produced fewer leaves than the SR1 vector control (vc). The 35S:*NtFT5*_G170W_//SR1∆*NtFT5* lines produced more leaves and the 35S:*NtFT5*_MM W170G_//SR1∆*NtFT5* lines produced fewer leaves than the corresponding 35S:*NtFT5*_(MM)_//SR1∆*NtFT5* lines. Individual data points (diamonds) are shown for three 35S:*NtFT5*_MM_//SR1∆*NtFT5* lines (L14 and L24, *n* = 10; L20, *n* = 8), three 35S:*NtFT5*//SR1∆*NtFT5* lines (L2, L3 and L4, *n* = 10), three 35S:*NtFT5*_MM W170G_//SR1∆*NtFT5* lines (L6, L7 and L8, *n* = 10), three 35S:*NtFT5*_G170W_//SR1∆*NtFT5* lines (L1, *n* = 8; L2 *n* = 10; L6, *n* = 6) and one vector control line (*n* = 10). Normal distribution was confirmed by applying the Kolmogorov-Smirnov test. Statistical significance was determined using a pairwise Welch’s *t*-test with Bonferroni-Holm correction (**P < 0.01; *P < 0.05; black = significant compared to vector control; gray = significant compared to corresponding native construct). In the boxplots in (**e**, **g**), center line = median, square = mean, box limits = upper and lower quartiles, whiskers = 1.5× interquartile range, and circles = outliers.

Next, we determined whether the C-terminal truncation of NtFT5_MM_ affects protein stability, as previously reported for C-terminal FT mutants in Arabidopsis^8^. First, crude protein extracts of T_1_ seedlings from three independent transgenic lines (as in Fig. 5) and SR1 WT seedlings as controls were used for western blotting. We detected NtFT5 and NtFT5_MM_ using the AtFT-specific antibody only in crude protein extracts of transgenic seedlings overexpressing the corresponding NtFT. Specific signals were present for one 35S:*NtFT5*//SR1∆*NtFT5* line (L3), as well as two 35S:*NtFT5*_MM_//SR1∆*NtFT5* lines (L14 and L24; Supplementary Fig. S3). The band for NtFT5_MM_ migrated further, reflecting its lower molecular weight due to the truncated C-terminus. To investigate protein stability in more detail, we used the TMV RNA-based overexpression (TRBO) vector for high-level transient expression of NtFT5 and NtFT5_MM_ proteins in *N. benthamiana*^26^. Snap-frozen leaf samples infiltrated with either pTRBO-*NtFT5* or pTRBO-*NtFT5*_MM_ (two independent infiltrations in each case) were incubated at room temperature for 0, 2, 5, 15, or 60 min and subsequently used for western blotting, with mock-infiltrated samples serving as controls (Fig. 6a; Supplementary Fig. S4). Distinct signals for NtFT5 and NtFT5_MM_ were detected using the AtFT-specific antibody in crude protein extracts from infiltrated leaves incubated at room temperature for 0 or 2 min. In the mock-control, only tubulin was detected. All signals faded over time and were barely detectable in samples kept for 60 min at room temperature (Fig. 6a; Supplementary Fig. S4). This demonstrated that the truncated C-terminus of NtFT5_MM_ does not appear to affect protein stability.

### Assessment of NtFT5_MM_ phloem mobility

Next, we investigated whether the truncation of NtFT5_MM_ impairs its movement through the phloem, given that efficient FT transport is required for floral induction and certain FT mutants are known to be defective in long-distance mobility^9^. We therefore grafted SR1∆*NtFT5* scions onto rootstocks of either 35S:*NtFT5*//SR1∆*NtFT5* or 35S:*NtFT5*_MM_//SR1∆*NtFT5* lines as well as WT control plants (*n* = 3 each). All SR1∆*NtFT5* scions grafted onto WT control stocks flowered 7–9 weeks after grafting, with 35–49 leaves/nodes produced on the scion (Fig. 6b). After ~11 weeks, one SR1∆*NtFT5* scion grafted onto 35S:*NtFT5*//SR1∆*NtFT5* stock, and two grafted onto 35S:*NtFT5*_MM_//SR1∆*NtFT5* stock, showed floral-like structures, with 21 and 24/25 leaves/nodes produced on the scion, respectively (Fig. 6b). However, floral transition of the SAM occurred in only 50% of these grafts, and none formed complete inflorescences as induced by WT stocks. Because 35S:*NtFT5*//SR1∆*NtFT5* stocks frequently produced axillary shoots, which we removed regularly, this may have impaired source–sink transport to the scion and may explain why only one out of three scions formed floral-like structures. For the 35S:*NtFT5*_MM_//SR1∆*NtFT5* stocks, the appearance of floral-like structures on two of three scions is consistent with the transport of NtFT5_MM_ to the shoot apex, although it remains functionally impaired compared to NtFT5. Accordingly, floral transition mediated by 35S:*NtFT5*_MM_//SR1∆*NtFT5* stocks was slightly delayed compared to the SR1∆*NtFT5* scion grafted onto a 35S:*NtFT5*//SR1∆*NtFT5* stock, providing additional evidence that NtFT5_MM_ is a weaker floral inducer.

### Analysis of FT–FD complex formation using BiFC and mcBiFC

Given that the stability and phloem mobility of NtFT5_MM_ is apparently normal, we wanted to gain more insight into the mechanistic difference between NtFT5 and NtFT5_MM_. We therefore tested for interaction with the recently identified tobacco FD proteins^20^. To initiate floral development, FT interacts with FD in the SAM^4,5,14,27^, and similar FT/FD interactions have been described in tobacco^20^. We conducted BiFC assays in which the C-terminal portion of the fluorescent reporter protein mRFP was fused to the N-terminus of NtFD1, NtFD3, NtFD4, or NtFD1 with a mutated STAPF motif (negative control NtFD1_SAAPF_) to remove a phosphorylation site and prevent interactions with FT^20^. The N-terminal portion of mRFP was fused to the N-terminus of NtFT5_MM_, NtFT5, the well-characterized AtFT as a positive control, and a truncated version of NtFT5 lacking the last 15 amino acids (NtFT5_FN*_; Supplementary Fig. S5) as a negative control. The CmRFP-FD proteins were co-expressed systematically with the NmRFP-FT proteins. As expected, CLSM revealed nuclear fluorescence for AtFT combined with any of the three native NtFD proteins (Supplementary Fig. S5) but not when NtFT5_FN*_ was combined with native NtFD proteins (Fig. 6c; Supplementary Fig. S5). Furthermore, nuclear fluorescence was detected when either NtFT5_MM_ or NtFT5 was combined with any of the three native NtFD proteins (NtFD1 shown as a representative example in Fig. 6c, for others see Supplementary Fig. S5) but not with NtFD1_SAAPF_ (Fig. 6d). We confirmed the presence of the mRFP-FT fusion proteins by western blot (Supplementary Fig. S6).

We conducted mcBiFC assays to test the binding affinity for NtFD1. Using this assay, we recently confirmed that floral repressor NtFT2 binds with lower affinity to NtFD1 compared to floral activator NtFT4^20^. We therefore prepared two cassettes in which CaMV 35S drives the expression of NtFT5 or NtFT5_MM_ as N-terminal fusions with the N-terminal component of cyan fluorescent protein S(CFP)3 A (SCN) together with an N-terminal fusion of NtFT5 or NtFT5_MM_ with the N-terminal component of Venus (VN) (Supplementary Fig. S7). A construct in which the N-terminal portion of NtFD1 was fused to the C-terminal portion of S(CFP)3 A (SCC) was carried by a separate bacterial strain. Interactions between SCN and SCC reconstitute S(CFP)3 A, which emits blue fluorescence when excited, whereas chimeric association between VN and SCC emits green fluorescence. After agroinfiltration and the cultivation of *N. benthamiana* under constant light for 3 days, abaxial epidermal leaf cells were analyzed by CLSM. The co-expression of SCN-NtFT5 and VN-NtFT5 with SCC-NtFD1, as well as SCN-NtFT5_MM_ and VN-NtFT5_MM_ with SCC-NtFD1, resulted in little difference between the blue and green signals and served as an equilibrium control with balanced competition (Fig. 6e; Supplementary Fig. S7). However, when SCN-NtFT5, VN-NtFT5_MM_ and SCC-NtFD1 were co-expressed, a significant shift towards the SCN/SCC blue signal was detected, whereas the co-expression of SCN-NtFT5_MM_, VN-NtFT5 and SCC-NtFD1 produced a dominant green VN/SCC signal. This suggests NtFT5_MM_ has a lower binding affinity than NtFT5 for NtFD1, and that NtFD1 may preferably associate with NtFT5 because it is more competitive at the protein level than NtFT5_MM_ with its truncated C-terminus.

Our results show that the C-terminal residues of NtFT5 fulfill an important role in floral transition. Arabidopsis mutants with the substitution G171E in the corresponding region have a late flowering phenotype^14,27,28^. In NtFT5_MM_, a tryptophan residue replaces the corresponding glycine (G170W), so we designed overexpression constructs with reciprocal amino acid substitutions (*NtFT5*_G170W_ and *NtFT5*_MM W170G_) and analyzed these in transgenic SR1∆*NtFT5* lines (Supplementary Fig. S8a). We regenerated three independent 35S:*NtFT5*_G170W_ and 35S:*NtFT5*_MM W170G_ transgenic lines each and investigated these lines in the T_1_ generation compared to 35S:*NtFT5* and 35S:*NtFT5*_MM_ transgenic lines (Fig. 6f, g; Supplementary Fig. S8b,d). We cultivated three independent transgenic lines each (*n* = 6–10) and one SR1 vector control (*n* = 10) in the greenhouse under LD conditions and documented the flowering phenotype for one representative transgenic line each in comparison to a vector control (Fig. 6f). *NtFT5*_G170W_ and *NtFT5*_MM W170G_ mRNA levels were much higher than the vector control, in the range of the *NtFT5*_MM_ mRNA overexpression levels (Supplementary Fig. S8b). Immunodetection confirmed the presence of the corresponding proteins in all transgenic lines (Supplementary Fig. S8c). However, *NtFT5*_MM W170G_//SR1∆*NtFT5* lines flowered significantly earlier and the number of leaves was significantly lower compared to the 35S:*NtFT5*_MM_//SR1∆*NtFT5* lines (Fig. 6g; Supplementary Fig. S8d). Accordingly, 35S:*NtFT5*_G170W_//SR1∆*NtFT5* lines flowered later than 35S:*NtFT5*//SR1∆*NtFT5* overexpressing plants, resulting in the production of significantly more leaves and significantly more days until flowering (Fig. 6g; Supplementary Fig. S8d). This indicates that both substitutions at position 170 (tryptophan to glycine in NtFT5_MM W170G_ and glycine to tryptophan in NtFT5_G170W_) produced floral inducers stronger than NtFT5_MM_ but weaker than NtFT5.

## Discussion

Many plant species initiate the developmental switch from vegetative to reproductive growth under specific photoperiodic conditions. Although tobacco is a day-neutral species, floral initiation depends on the photoperiod-dependent expression of various *FT* genes^16,20^. Previous studies have mostly focused on *FT* genes expressed under SD conditions, but the recently identified *NtFT5* gene is expressed under both SD and LD conditions. NtFT5 has been characterized as the major floral activator under LD conditions in tobacco cultivar SR1, making this protein an interesting candidate for further analysis in the SD cultivar MM^20,22^.

Comparative expression analysis revealed that *NtFT5* is expressed at similar levels in cultivar MM and the day-neutral cultivar Hicks, which excludes the possibility that the LD-specific non-flowering phenotype of MM is caused by the silencing of this locus (Fig. 1i, j). We also found that the *NtFT5* coding sequence was identical in cultivars Hicks and SR1, and that the overexpression of this sequence in MM permitted flowering under LD conditions (Fig. 2), suggesting that the NtFT5_MM_ protein may be functionally impaired. Further analysis revealed a 2-bp deletion near the 3ʹ end of the *NtFT5*_MM_ gene causing a frameshift that introduced a premature termination codon and an altered C-terminus (Fig. 3). By analyzing the F_2_ offspring of reciprocal crosses between Hicks and MM, we found that a homozygous 2-bp deletion in the *NtFT5*_MM_ gene is tightly associated with the non-flowering phenotype under LD conditions whereas flowering is delayed in heterozygous plants (Fig. 4). A similar effect was also described for *NtFT5* knockout mutations^22^. The mutated NtFT5_MM_ protein lacks the six amino acids normally found at the C-terminus and has five substitutions at the new C-terminus (Fig. 3). The NtFT5_MM_ protein was detected by western blot, and floral structures were induced in the SAM of SR1∆*NtFT5* scions grafted onto 35S:*NtFT5*_MM_//SR1∆*NtFT5* stocks (Fig. 6a, b; Supplementary Fig. S3), indicating that neither protein stability nor transport is affected by the C-terminal truncation and alteration of NtFT5_MM_. We tested the ability of NtFT5 and NtFT5_MM_ to induce flowering under LD conditions by expressing each variant in SR1∆*NtFT5* plants. As expected, *NtFT5* overexpression resulted in profound early flowering (Fig. 5b–e), as described for transgenic tobacco overexpressing other floral activators such as NtFT4 and AtFT^16,20^. In contrast, the overexpression of *NtFT5*_MM_ caused plants to flower at a later developmental stage, even though *NtFT5*_MM_ mRNA was more abundant than *NtFT5* mRNA in the corresponding transgenic SR1∆*NtFT5* plants (Supplementary Fig. S2a, b). The late-flowering of transgenic 35S:*NtFT5*_MM_//SR1∆*NtFT5* lines compared with the normal flowering time of SR1 vector controls (expressing *NtFT5* at endogenous levels) showed that a fully functional endogenous *NtFT5* gene can more effectively induce flowering than the strong overexpression of *NtFT5*_MM_. Accordingly, we found that only the expression of *NtFT5* under the control of its endogenous promoter could complement the non-flowering phenotypes of SR1∆*NtFT5* and MM plants, whereas the expression of P-NtFT5_2.6kb_:*NtFT5*_MM_ did not induce flowering (Fig. 5f; Supplementary Fig. S2c). This suggests that the truncated and altered C-terminus of *NtFT5*_MM_ weakens its function as a floral activator and may affect its ability to bind interaction partners from the FAC complex.

**Fig. 7:**
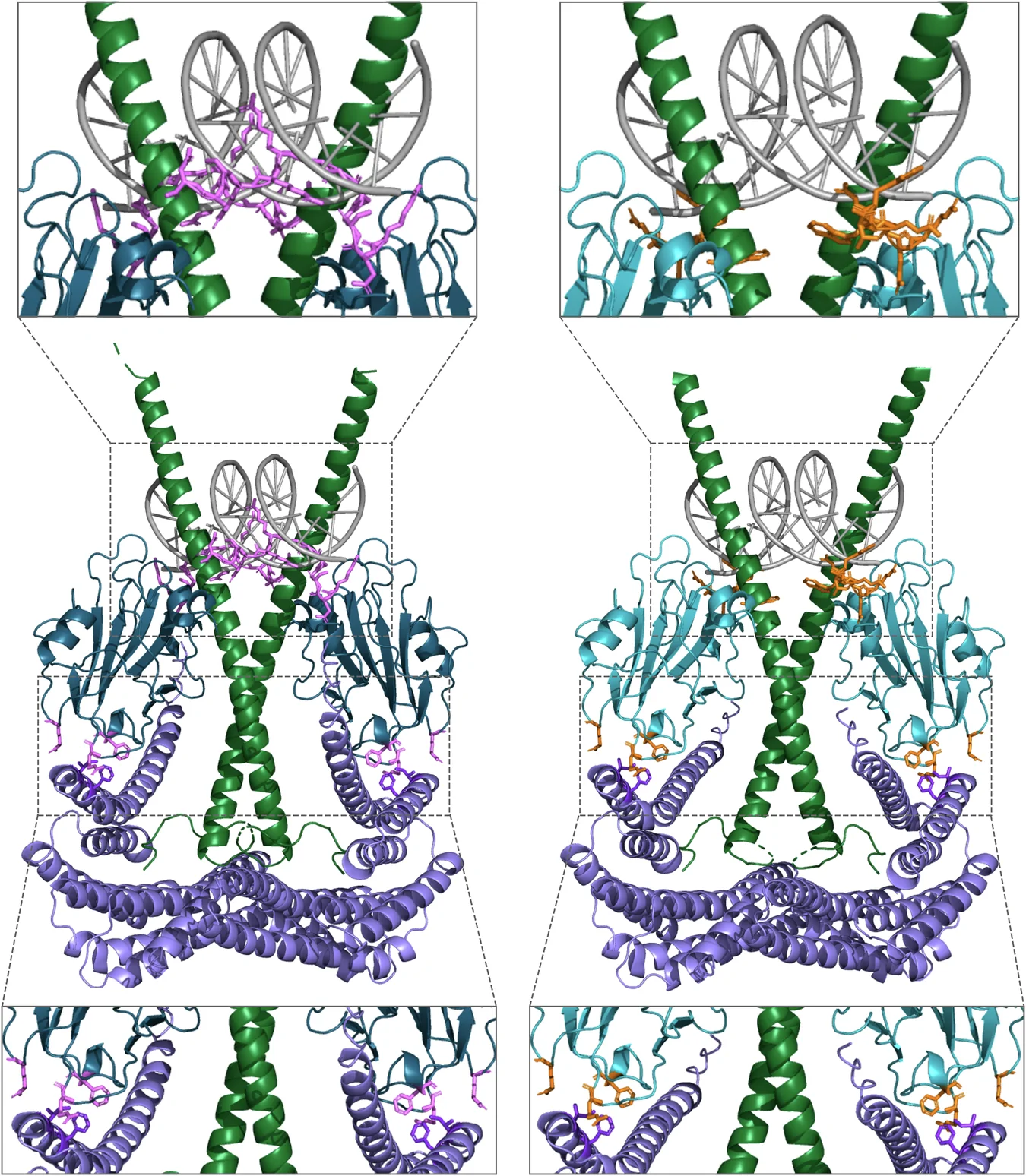
Predicted structures of the FAC–DNA complexes comprising NtFT5, NtFD1 and 14-3-3c (left) or NtFT5_MM_, NtFD1 and 14-3-3c (right). Across all modeled complexes, FT surfaces that interact with 14-3-3 proteins adopt highly similar orientations and positions (bottom). The C-terminal region of NtFT5 extends into and associates with the DNA (top left), whereas the truncated and altered C-terminus of NtFT5_MM_ does not associate with the DNA (top right), probably reflecting the reduced net positive charge and additional steric constraints. Complexes were modeled with AlphaFold 3^25^ using the DNA sequence TTGA**CACGTG**GTCAA (with a G-box motif for FD binding in bold) and two copies each of FT (in dark or light blue), FD (in green), and 14-3-3 proteins (in purple). The structure was visualized using PyMOL v2.4.0. C-terminal regions are highlighted in pink for NtFT5 (residues 165–177; RESGSGGRRRSAD) and in orange for NtFT5_MM_ (residues 165–171; RERQWWT). FT residues critical for 14-3-3 interaction^29,30^ are shown in pink for NtFT5 (R61, F100, R129) and in orange for NtFT5_MM_ (R61, F100, R129). Key 14-3-3 residues involved in FT binding^30^ are highlighted in purple for Nt14-3-3c (F205, I209).

As previously shown in Arabidopsis and rice (*Oryza sativa*), FT proteins interact with FD proteins to regulate flower development^4,5,29^. Similarly, tobacco FT proteins interact with three FD proteins, and it was therefore important to determine whether the truncated NtFT5_MM_ protein retained this ability^20^. Our BiFC experiments showed that both NtFT5 and NtFT5_MM_ interacted with all three tobacco FD proteins, but mcBiFC experiments revealed that NtFD1 may preferably associate with NtFT5 because it is more competitive than NtFT5_MM_ at the protein level (Fig. 6c–e; Supplementary Fig. S5). Because residues mediating 14-3-3-dependent FT–FD interaction are conserved upstream of the 2-bp deletion in NtFT5_MM_ (Fig. 7, bottom panels), our results suggest that additional C-terminal residues are required for strong interaction. These genetic findings are consistent with a model in which the C-terminal tail of AtFT (especially the positively charged residues R166, R173 and R174) mediates direct DNA contacts required for the recruitment of AtFT to AtFD–14-3-3 and the subsequent assembly of the FAC on target promoters^30^. In addition to the three arginine residues in AtFT, NtFT5 contains a fourth arginine at the C-terminus, and AlphaFold modeling shows that these residues probably make direct contacts with DNA (Fig. 7, top panels). In contrast, NtFT5_MM_ lacks the C-terminal arginine cluster (R172–174) and contains two tryptophan residues within its truncated C-terminus, which may interfere with the ability of FAC to interact with DNA by reducing the net positive charge and causing steric hindrance (Fig. 7, top panels). Truncation and mutation at the C-terminus therefore appear to inhibit FAC assembly despite normal transcription, stability, and transport. In line with the updated FAC model, the strong overexpression of a C-terminally truncated and mutated FT variant, such as NtFT5_MM_, cannot fully accomplish efficient recruitment to FD-14-3-3–DNA in the apex, explaining the partial complementation with delayed flowering.

Furthermore, the C-terminal residues of NtFT5 fulfill an important role in floral activation, as shown by the late-flowering phenotype of AtFT mutants with the substitution G171E. In NtFT5_MM_, the corresponding glycine residue is replaced with tryptophan (G170W), suggesting that the truncated and altered C-terminus of NtFT5_MM_ may influence its recruitment to the FD-14-3-3–DNA complex. Indeed, replacing the tryptophan residue in NtFT5_MM_ with glycine in transgenic 35S:*NtFT5*_MM W170G_//SR1∆*NtFT5* lines restored WT flowering behavior, whereas the converse mutation in transgenic 35S:*NtFT5*_G170W_//SR1∆*NtFT5* lines abolished the profound early flowering phenotype (Fig. 6f, g), consistent with steric constraints imposed by tryptophan at this position. Experiments in Arabidopsis have shown that the overexpression of a truncated *FT* gene encoding a protein lacking the last seven amino acids (FTΔ168) causes a late flowering phenotype in *ft-10* mutant plants compared to *ft-10* plants overexpressing functional *FT*^8^. FTΔ168 and NtFT5_MM_ both lack the arginine residues at R173 and R174 and the glycine residue at position 170/171, which is strongly conserved in the FT-like family.

Interestingly, members of the TFL1-like family of floral repressors have a highly conserved C-terminus that lacks the glycine residue at the corresponding position (Supplementary Fig. S9). However, the glycine residue is present in both floral activators and floral inhibitors of the FT-like family, so its precise function in terms of floral regulation remains unclear. Additional amino acid residues that are necessary for the regulatory activity of FT proteins have been described^30–33^, and it seems likely that the presence of all these conserved residues is required for the correct function of FT-like floral activators. According to the updated FAC model, alterations in the FT C-terminal region may weaken FT recruitment to the FD–14-3-3–DNA complex, which is consistent with the delayed-flowering phenotypes seen in C-terminal FT variants^30^.

*NtFT5* knockout lines and MM plants can flower under SD conditions due to the presence of the functional floral activator NtFT4, whereas *NtFT4/NtFT5* knockout lines do not flower under LD and SD conditions^34^. Flowering under SD and LD conditions is therefore strictly dependent on NtFT proteins and is not regulated by other signaling pathways, such as hormonal pathways. With regard to MM, this means that the high levels of mRNA encoding the strong activator NtFT4 and the weak activator NtFT5_MM_ under SD conditions are sufficient to redundantly induce flowering, whereas the low levels of both transcripts under LD conditions are not (Fig. 1i, j). Threshold levels for tobacco FTs have already been described in the context of floral activation and repression^16^. In line with this, only the strong overexpression of *NtFT5*_MM_ can overcome the non-flowering phenotype in SR1∆*NtFT5* plants (Fig. 5). However, even strong overexpression can only complement the non-flowering phenotype to a certain extent, given that the transgenic 35S:*NtFT5*_MM_//SR1∆*NtFT5* plants still induce flower development at a later time point compared to the SR1 vector control. Under SD conditions, the MM plants flowered 1 week later than the day-neutral cultivar Hicks (Fig. 1). RNAi experiments in tobacco SR1 plants have shown that the silencing of *NtFT4* or *NtFT5* can delay flowering, but the effect was stronger for *NtFT5*^20^. This suggests NtFT5 has a stronger effect than NtFT4 in flower development and explains the delayed flowering phenotype of cultivar MM compared to day-neutral cultivar Hicks under SD conditions, given that the latter still produces a functional NtFT5 protein.

Taken together, our results demonstrate that NtFT5_MM_ acts as a weak floral activator even when strongly overexpressed. Accordingly, we propose a model for the MM phenotype in which, under LD conditions, NtFT5_MM_ is not efficiently recruited to the target DNA-bound FD–14-3-3 complex at the shoot apex (Fig. 7), thereby reducing florigen output. Under SD conditions, due to functional redundancy, the high levels of *NtFT4* expression compensate for the weakened NtFT5_MM_ activity, thereby enabling the transition to reproductive growth. Thus, our results provide a molecular explanation for the MM locus with the 2-bp deletion in *NtFT5* and give insight into the SD-specific flowering behavior of MM, a tobacco cultivar that was instrumental in the discovery of photoperiodism and was first described more than a century ago.

## Materials and methods

### Plant materials and cultivation conditions

Seeds of day-neutral *N. tabacum* cv. Hicks, strict SD flowering *N. tabacum* cv. Maryland Mammoth (IPK Gatersleben, Germany) and strict SD flowering *N. tabacum* SR1∆*NtFT5* were sown in soil and cultivated in the greenhouse under LD conditions (16-h photoperiod, artificial light on if natural light fell below 700 μmol m^−^^2^ s^−1^, 22–25 °C in the light, 20–25 °C in the dark) or in phytotrons under SD conditions (8-h photoperiod, 200 μmol m^−2^ s^−1^, 25 °C in the light, 22 °C in the dark). Medial leaves (fourth or fifth true leaf counted from the bottom) were harvested 6 and 10 weeks after seed sowing (WASS) under LD and SD conditions, and each sample comprised tissue pooled from three plants.

Transgenic tobacco plants were generated by leaf disc transformation^35^ using *Agrobacterium tumefaciens* strain LBA4404^36^ following electroporation with the appropriate plasmids, and were regenerated on MS medium^37^ containing 25 mg/L hygromycin for sterile selection under LD conditions (100 μmol m^−2^ s^−1^, 23 °C). After root formation, independent transgenic lines were transferred to the greenhouse and grown in soil. T_1_ seedlings were cultivated under sterile conditions on selective medium as above. Seedlings were either harvested for mRNA/protein expression analysis or up to 10 individuals were transferred to the greenhouse for phenotyping.

### Gene expression studies

Samples were snap-frozen in liquid nitrogen and stored at –80 °C. Total RNA was extracted using the innuPREP Plant RNA kit (Analytik Jena, Jena, Germany) and residual genomic DNA was removed using the Turbo DNA-free kit (Thermo Fisher Scientific, Waltham, MA, USA). First-strand cDNA was synthesized using PrimeScript RT Master Mix (Takara, Saint-Germain-en-Laye, France) followed by quantitative real-time PCR (qPCR) analysis using the CFX 96 Real-Time system (Bio-Rad, Munich, Germany) on a C1000 Touch Thermal Cycler. We mixed 0.1 µl template cDNA and 500 nM of each primer (Supplementary Table S2) with KAPA SYBR Fast qPCR Master Mix (Merck, Darmstadt, Germany). Each reaction comprised an initial denaturation step for 3 min at 95 °C, followed by 40 cycles of denaturation at 95 °C for 3 s and annealing/extension at primer-specific temperatures for 30 s (Supplementary Table S2). Melt curve analysis was carried out to confirm the specificity of the amplicon (5 s, 58–95 °C, ΔT = 0.5 °C). The target transcripts and reference *NtEF-1α*^38^ were analyzed in triplicate, and the quantification cycle (C_q_) values of technical replicates were averaged. The NTC (non-template control) and NRT (non-reverse-transcribed control) were analyzed in duplicate. Data were analyzed using Bio-Rad CFX Manager v3.1. *NtFT4* and *NtFT5* expression levels were determined using 2^-ΔΔCT^ Method^39^.

### Cloning experiments

*NtFT5* coding sequences were amplified from tobacco cDNA (*N. tabacum* cv. Hicks and cv. MM) and *AtFT* from Arabidopsis cDNA (*A. thaliana* cv. Col-0) using the primers listed in Supplementary Table S2. The overexpression constructs were prepared by digesting the *NtFT5* and *NtFT5*_MM_ amplicons with XhoI/XbaI and inserting them into vector pRT104 containing the CaMV 35S promoter and terminator^40^. The expression cassette was then excised with HindIII and ligated into vector pBinHyg^22,41^. The *NtFT5* and *NtFT5*_MM_ expression constructs driven by endogenous P-NtFT5_2.6kb_ were prepared by digesting P-NtFT5_2.6kb_ with XmaI/XhoI and the *NtFT5* and *NtFT5*_MM_ amplicons with XhoI/XbaI, and inserting them into vector plab12.1 containing the 35S terminator (linearized with XmaI/XbaI). The overexpression constructs for agroinfiltration were prepared by digesting the *NtFT5* and *NtFT5*_MM_ amplicons with PacI/NotI and inserting the products into vector pTRBO^26^.

For bimolecular fluorescence complementation (BiFC) assays, the *AtFT, NtFT5* and *NtFT5*_MM_ coding sequences were amplified using the primers listed in Supplementary Table S2, digested with SalI/NotI or XhoI/XbaI and inserted into vector pENTR4 (Invitrogen, Karlsruhe, Germany). The pENTR4 constructs were then transferred to pBatTL NmRFP-*ccd*B by LR recombination using the Gateway LR Clonase Enzyme mix (Invitrogen). Split mRFP destination vectors containing *NtFD1*, *NtFD3, NtFD4* and NtFD1_SAAPF_ were already available^20^. Destination vectors were kindly provided by Dr Guido Jach and Dr Joachim Uhrig, MPI Cologne, Germany^42^. To generate a suitable negative control for BiFC, different NtFT5 truncations and amino acid substitutions were generated in regions considered important for the interaction between rice FT and FD^29^ (Supplementary Fig. S5). To generate the *NtFT5*_R61G_ and *NtFT5*_R129A_ constructs, we used overlapping primers to introduce the mutation (Supplementary Table S2) and the *NtFT5* sequence cloned in vector pENTR4 as the template. Parental plasmids were digested with DpnI. Of the many chimeric NtFT5 proteins, only the shortest NtFT5 version we tested (NtFT5_FN*_) did not interact with NtFD1 and was therefore selected as the negative control for BiFC analysis. For the multicolor BiFC (mcBiFC) assay, P35S:SCN-NtFT2:Tnos-Spacer and P35S:VN-NtFT2:Tnos pBluescriptII^18^ were digested with StuI/BsrGI and ligated with the *NtFT5* and *NtFT5*_MM_ coding sequences to generate vectors pBS-*SCN-NtFT5*-Sp, pBS-*SCN-NtFT5*_MM_-Sp, pBS-*VN-NtFT5* and pBS-*VN-NtFT5*_MM_. To generate dual expression cassettes, P35S:*VN-NtFT5*_(MM)_ was excised using ClaI/DraIII and transferred to pBS-*SCN-NtFT5*_(MM)_-Sp to create four intermediate vectors. The dual expression cassettes were excised using PmeI/BamHI and transferred to plab12.1 (linearized with XmnI/BamHI) to create four binary vectors named plab-*SCN-NtFTx*-Sp-*VN-NtFTx*. To generate the SCC-NtFD1 fusion, SCC-HA was amplified using the primers listed in Supplementary Table S2, digested with SalI/BamHI and transferred to pRT104 (linearized with XhoI/BamHI). The NtFD1 coding sequence was amplified, digested with BamHI/XbaI and transferred to pRT-SCC-HA (linearized with BamHI/XbaI). The P35S:SCC-NtFD1:T35S cassette was excised with HindIII and transferred to plab12.1 to create the binary vector plab-SCC-NtFD1.

The overexpression constructs for *NtFT5*_G170W_ and *NtFT5*_MM W170G_ were prepared by digesting the *NtFT5*_G170W_ and *NtFT5*_MM W170G_ amplicons (generated using the primers listed in Supplementary Table S2) with XhoI/XbaI and inserting them into vector pRT104 containing the CaMV 35S promoter and terminator^40^. The entire expression cassette was then excised with HindIII and transferred to vector plab12.10^43^.

### Fragment length analysis

F_1_ and F_2_ individuals of the reciprocal cross of Hicks and MM were genotyped by fragment length analysis (FLA) of the *NtFT5* genomic sequence using a fragment of exon 4 containing the *NtFT5*_MM_-specific 2-bp deletion. The fragment was amplified using primers (see Supplementary Table S2) carrying the fluorescent dye 6-carboxyfluorescein (6-FAM). To identify *NtFT5* and *NtFT5*_MM_ alleles, the amplicon lengths of the *NtFT5* and *NtFT5*_MM_ alleles from cultivars Hicks and MM, respectively, were simultaneously determined and used as references. The PCR samples were mixed with the GeneScan 600 LIZ dye Size Standard v2.0 (Thermo Fisher Scientific) and analyzed in an ABI 3730 Genetic Analyzer (Applied Biosystems, Waltham, MA, USA). Data were evaluated using Applied Biosystems Sizing Analysis Module Peak Scanner Software v3.0.

### (Multicolor) bimolecular fluorescence complementation analysis

Transient expression was carried out by co-infiltrating the leaves of 3–4-week-old *N. benthamiana* plants (grown under LD conditions) with *A. tumefaciens* strain GV3101 pMP90 carrying the appropriate expression constructs for FT (*AtFT*, *NtFT5*, *NtFT5*_MM_, *NtFT5*_FN*_, *NtFT5*_FNC*_, *NtFT5*_R61G_ or *NtFT5*_R129A_) and FD (*NtFD1*, *NtFD1*_SAAPF_, *NtFD3* or *NtFD4*) and *A. tumefaciens* strain C58C1 carrying the pCH32 helper plasmid containing virulence genes and the pBIN61 plasmid encoding the tomato bushy stunt virus RNA silencing suppressor protein p19^44,45^. The expression and helper strains were infiltrated at OD_600nm_ = 1 and 0.3, respectively. We monitored mRFP fluorescence by confocal laser scanning microscopy (CLSM) using a TCS SP5 X microscope (Leica Microsystems, Wetzlar, Germany) at excitation and emission wavelengths of 549 and 569–629 nm, respectively. For mcBiFC^46,47^, the SCN/VN expression strains, the SCC-NtFD1 expression strain and the helper strain were infiltrated at OD_600 nm_ = 0.5, 0.33 and 0.3, respectively. Plants were cultivated under continuous light for 3–4 days, and leaf discs were screened for fluorescent cells in the abaxial epidermis. Fluorescence was observed by CLSM using a Leica STELLARIS 8 microscope (Leica Microsystems) at excitation/emission wavelengths of 458/470–490 nm for S(CFP) 3 A and 488/505–525 nm for the VN/SCC chimera. Nuclear fluorescence was quantified using Leica LAS AF software, and the data were analyzed using Origin 2020.

### Protein stability assay

Transient overexpression was carried out by agroinfiltration using *A. tumefaciens* strain GV3101 pMP90 carrying the expression constructs pTRBO-*NtFT5* or pTRBO-*NtFT5*_MM_ and *A. tumefaciens* strain C58C1 carrying the pCH32 helper plasmid and pBIN61 plasmid as above. The expression and helper strains were infiltrated at OD_600 nm_ 1 and 0.6, respectively. After 3 days, infiltrated leaves were snap-frozen in liquid nitrogen, and 100 mg frozen leaf material was kept at room temperature for 0, 2, 5, 15, or 60 min. We then added 100 µl Laemmli buffer (50% glycerol, 10% SDS, 0.25 M Tris-HCl pH 6.8, 5% 2-mercaptoethanol) containing 0.05% bromophenol blue. The samples were vortexed for 2 min, denatured by heating to 100 °C, and separated by SDS-PAGE (10 µl per lane) before western blotting.

### Western blotting

Total protein extracted from 3-week-old transgenic and wild-type (WT) seedlings or from infiltrated leaves (BiFC and protein stability experiments) was heated to 100 °C in Laemmli buffer containing 0.05% bromophenol blue and separated by SDS-PAGE on a 12% polyacrylamide gel before electroblotting onto a nylon membrane. FT proteins were detected using an anti-FT antibody (AS06198; Agrisera, Vännäs, Sweden) diluted 1:1000, and an alkaline phosphatase-conjugated goat anti-rabbit IgG secondary antibody (G-21079; Invitrogen) diluted 1:10,000. Tubulin was detected using an α-tubulin-specific antibody (AA4.3; Developmental Studies Hybridoma Bank, Iowa City, IA, USA) diluted to 0.3 µg/ml, and an alkaline phosphatase-conjugated goat anti-mouse IgG secondary antibody (A3562; Sigma-Aldrich) diluted 1:10,000.

### Grafting experiments

SR1 WT and 35S:*NtFT5*_(MM)_//SR1∆*NtFT5* transgenic plants were cultivated in the greenhouse until floral buds became visible. Non-flowering SR1∆*NtFT5* scions were then grafted onto each stock (*n* = 3) before further cultivation in the greenhouse. Emerging leaves of the SR1∆*NtFT5* scions were removed regularly to enhance the source–sink gradient. To ensure seed production by P-NtFT5_2.6kb_:*NtFT5*//SR1∆*NtFT5* and P-NtFT5_2.6kb_:*NtFT5*_MM_//SR1∆*NtFT5*, scions of these primary transformants were grafted onto florally induced SR1 WT plants.

### Statistics and Reproducibility

Normal distribution of data was confirmed using the Kolmogorov-Smirnov test where sample size was sufficient. All statistical tests were two-sided. For comparisons among multiple groups, we applied one-way ANOVA with Bonferroni-Holm correction (*P* < 0.05). For pairwise comparisons between groups, we used a Welch’s t-test with Bonferroni-Holm correction (significance thresholds: *P < 0.05, **P < 0.01). Effect sizes are reported as η² (eta-squared) for omnibus ANOVA tests and as Cohen’s d for pairwise comparisons. Cohen’s d was calculated as the mean difference divided by the pooled standard deviation. Exact P values, test statistics (t or F values), degrees of freedom, and effect sizes are provided in the file Supplementary Data 1.

For gene expression analysis by qPCR, we tested *n* = 3 biological replicates per time point, each comprising pooled leaf tissue from three individual plants. For transgene expression analysis, samples were isolated from pooled seedlings (*n* = 5) or individual plants, and error bars represent the combined standard deviation of target and reference gene technical replicates. For phenotyping experiments (flowering time and leaf number), sample sizes ranged from *n* = 6 to *n* = 10 per transgenic line or control. We analyzed at least two independent transgenic lines per construct. For the multicolor BiFC assay, sample sizes ranged from 341 to 542 nuclei per condition.

Biological replicates are defined as independent plants grown and analyzed separately, unless otherwise stated (e.g., pooled tissue from multiple plants for qPCR). Independent transgenic lines (minimum of two per construct) were analyzed to confirm the reproducibility of phenotypic effects. Technical replicates refer to repeated measurements of the same biological sample.

Protein stability experiments were repeated independently with similar results. Western blots included appropriate loading controls (Ponceau S staining or Coomassie Brilliant Blue). Protein–protein interaction experiments (BiFC and mcBiFC) and grafting experiments were carried out at least twice independently with consistent results. Structural predictions were generated computationally and do not involve biological replicates.

### Reporting summary

Further information on research design is available in the Nature Portfolio Reporting Summary linked to this article.

## Supplementary information

Transparent Peer Review file

Supplementary Information

Description of Additional Supplementary Files

Supplementary Data 1

Reporting Summary

## Data Availability

All data supporting the findings of this study are available within the manuscript and its supplementary materials. Uncropped and unedited blot images of blots shown in Fig. 6a can be found in Supplementary Fig. S4. Source data underlying the graphs and charts presented in the figures are available in Supplementary Data 1.
