# Supplementary material for: A mutant allele of the long-day floral activator NtFT5 is associated with the short-day-specific flowering of tobacco cultivar Maryland Mammoth
Source: Commun Biol. 2026 Aug 26;9:1144. doi: 10.1038/s42003-026-10729-4 (PMC13507162; doi:10.1038/s42003-026-10729-4)
Supplement: Supplementary file 1 [file 42003_2026_10729_MOESM1_ESM.pdf]

nature portfolio

Peer Review File

A mutant allele of the long-day floral activator NtFT5 is associated with the short-day-specific flowering of tobacco cultivar Maryland Mammoth

Corresponding Author: Professor Gundula Noll

This file contains all reviewer reports in order by version, followed by all author rebuttals in order by version.

Version 0:

Reviewer comments:

Reviewer #1

(Remarks to the Author)
Major Concerns:
The manuscript by Lena Grundmann et al. presents a causal mutant allele responsible for the short-day-specific flowing of the Maryland Mammoth (MM) cultivar. The authors focus on the flowering activator FT5, comparing the wild-type FT5 and FT5MM for their endogenous expression profile, transgenic flowering photoperiod, and the role of FT5's C-terminus in its interaction with FD1 to promote flowering. They claim that FT5MM is the causal mutant allele for responsible for the MM short-day flowering.
The MM cultivar is instrumental in the discovery of photoperiodism in plants, but the gene affected by the Maryland Mammoth mutation is not known yet. Thanks to extensive research on Arabidopsis, the conserved photoperiod pathway is now relatively well-understood. While the activator FT5 is not a novel finding, the authors' identification its relationship to the extreme flowing phenotype of MM provides a crucial piece in solving a century-old puzzle.
This manuscript presents two main conclusions - that FT5 is the causal mutation and changes in its C-terminus affect its interaction with FD1. However, the paper is burdened with extraneous text that distracts from the main message. The manuscript lacks clear organization, and the evidence is not presented coherently. Specifically, the third section of the results is overly long, and difficult to follow.

Detailed comments:
1. The third result section, "Functional characterization of NtFT5MM" is too long, and should be divided into several sub-sections for better readability.
2. L393, "To gain more insight into the mechanistic difference between NtFT5 and NtFT5MM", authors first perform BiFC to test the interaction between two FT5s and FD1, then shift to protein stability and protein transfer analyses, and later used multicolor BiFC to differentiate their interactions to FD1. This sequence is somewhat confusing, as the protein stability and transfer experiments divert attention from the key focus on the interaction analysis. These latter experiments should be removed or relocated.
3. The key question regarding the FTMM's mutation at its C terminus is how it is associated to MM's short-day response. I noticed that the C-terminus contains several positive R, like a nucleus localization sequence (NLS). It is worth considering whether a deficient NLS could attenuate nucleus import, thereby reducing the FT5MM interaction with FD1 and impacting its function. After all, the flowering phenotypes of FT5MM W170G and NtFT5G170W are not same as the NtFT5 transgenic lines (Fig 5g), which suggests other residues, aside from G170, may play important roles.
4. As interaction test between FT5 and FD1, other assays like Y2H can be included.
5. The FT7 data seems to be tangential and could be moved to the discussion section. PCR cannot definitively exclude its presence in MM, especially in the absence of amino acid and DNA sequence information.
6. The figure 1i) and 1j) is difficult to interpret the floating bars. It would be clearer to present FT4 and FT5 in separate plots.
7. L479, ref "Beinecke et al, 2018)" is ref#18.
8. L347, what is the source of P-NtFT5, from Hicks or MM? Comparing the two FT versions driven by the endogenous FT5MM promoter and their respective flowering photoperiods is crucial evidence for the claims.
9. L356, why only a subset lines (3/10, 4/10, and 7/10) recover their long-day photoperiod?
10. I do not see the method description of FLA analysis.
11. L400, which 15 amino acids are deleted in FT5*?

12. Why the WB signal in Fig S6 is not specific as in FigS5.

Reviewer #2

(Remarks to the Author)
This study provides a systematic elucidation of the molecular mechanism behind the non-flowering phenotype in the tobacco variety Maryland Mammoth (MM) under long-day conditions. It demonstrates that a 2-bp deletion in the key flowering activator NtFTS leads to a C-terminal truncation, which compromises its interaction with NtFD1 and ultimately impairs flowering induction. The research is methodologically sound and supported by a comprehensive dataset, underscoring its biological significance. However, there is potential for improvement in experimental logic, data presentation, and the depth of discussion.
Specific suggestions are outlined below:
(1) Introduction
The historical background of the MM variety is somewhat lengthy and could be condensed.
The overview of FT/FD interactions in tobacco lacks systematic organization; incorporating recent advances in this area is recommended.
The relationship between this study and prior work (e.g., Beinecke et al., 2018) should be explicitly articulated.
(2) Results
Fig. 1i–j: The correlation between NtFT4/5 expression trends and phenotypic changes is not immediately evident. Including a time-course expression heatmap along with statistical analysis of expression data would help clarify whether the observed differences are significant.
Fig. 3a: The sequence alignment should highlight the specific mutation site and its potential location within the protein structure (e.g., within a functional domain).
Grafting experiment (Fig. 5d): The current sample size (n = 3) is relatively small; increasing the number of biological replicates would strengthen the reliability of the conclusions.
Point mutation rescue experiment (Fig. 5f–g): While the results are clear, it would be beneficial to confirm whether the expression levels of the mutant protein are comparable to those of the wild type.
Quantitative analysis of the binding affinity (e.g., KD values) between NtFTS/NtFTSMM and NtFD1 using methods such as yeast two-hybrid or surface plasmon resonance (SPR) is recommended.
Analyzing expression changes of downstream flowering-related genes (e.g., AP1/FUL homologs) in the shoot apical meristem (SAM) could help determine whether the FT mutation affects the expression intensity of downstream targets.
Using AlphaFold2 to predict structural differences between NtFT5 and NtFT5MM is advisable.
(3) Discussion
A deeper exploration is needed to explain why NtFTSMM still promotes flowering under short-day conditions, including an analysis of potential functional redundancy or synergy with other FT members (e.g., NtFT4).
The discussion on how C-terminal truncation affects FD binding ability is insufficient. Incorporating existing structural knowledge of FT proteins to formulate reasonable hypotheses is recommended.
The article does not address whether NtFTSMM might indirectly regulate flowering through other signaling pathways (e.g., hormonal pathways).
The explanation for the delayed flowering of the MM variety under short-day conditions is inadequate. Considering the involvement of additional regulatory factors in this process is suggested.
(4) Language and Format
Some sentences are excessively long, impairing readability (e.g., lines 78–82). Splitting these into shorter sentences is recommended.
Reference formatting is inconsistent; some journal names are abbreviated non-standardly (e.g., "Frontiers in Plant Sciences" should be "Front Plant Sci").

Reviewer #3

(Remarks to the Author)
This manuscript addresses a classic, century-old question in plant biology the molecular basis for the short-day (SD) flowering requirement and long-day (LD) gigantism of the Nicotiana tabacum cv.'Maryland Mammoth (MM). The authors convincingly demonstrate that this phenotype is associated with a 2-bp deletion in NtFT5, the major LD floral activator. This deletion results in a frameshift and a truncated protein NtFT5MM that, while stable and mobile, functions as a weak floral inducer. Using genetic crosses, complementation assays, and protein interaction studies, the authors propose a model where the reduced binding affinity of NtFT5MM for the floral partner NtFD1 drops the total floral activation signal below the necessary threshold for flowering under LD conditions.
The work is scientifically sound, well-structured, and provides a highly satisfying solution to a long-standing mystery. The combination of genetic, molecular, and biochemical evidence is a significant strength. My recommendations below are intended to further strengthen the mechanistic claims of the manuscript.

1. For the SR1 material, more relevant background should be provided for a better understanding of this work?
2. The tight segregation of the homozygous NtFT5MM allele with the non-flowering phenotype in F2 crosses (Fig. 4) provides a clear genetic link.
3. For the gene expression levels of NtFT4 and NtFT5 in Fig. 1i and 1j, should statistical analysis be performed? For the the gene expression difference in one column such as NtFT5 in 6 WASS Hicks, why the difference is so large? Biological replicates performed or not?

4. The last concern is the circadian gene and protein pattern of NtFT5 in MM and Hicks. Have ever been published? Just wonder if NtFT5 and NtFT5MM protein behave circadian rhythm.
5. In the introduction, Hicks which is isogenic to MM except at the NtFT5 locus. Right? Not the MM locus? In the method, what are medial leaves?

Version 1:

Reviewer comments:

Reviewer #1

(Remarks to the Author)
The structure predicted using AlphaFold does not appear to provide additional mechanistic insight in its current form (Fig. 3a). Unless interaction interfaces or specific functional predictions are analyzed and discussed.

The manuscript lacks sufficient background to justify why protein stability and transfer were examined. Neither the Introduction nor the Results section clearly explains the biological reasoning or hypothesis underlying these experiments.

In Figure 6a, the protein level of FT5MM appears relatively weaker even at the 0 min time point. This raises the possibility that the protein may already be expressed at low levels or be intrinsically unstable prior to treatment.

The results of protein stability and transfer should be presented in two separate paragraphs.

Reviewer #2

(Remarks to the Author)
This manuscript is acceptable, I have no further questions

Reviewer #3

(Remarks to the Author)
The authors have significantly improved the clarity and logical flow of the manuscript by restructuring the Results section and partitioning the functional characterization of NtFT5MM into well-defined subsections. The reorganization of experimental data—placing protein stability and transport assays before the mechanistic dissection of the Florigen Activation Complex (FAC)—provides a much more coherent narrative that mirrors the biological progression of the florigen signal. Furthermore, the integration of recent structural insights from Gao et al. (2025) strengthens the theoretical framework of the paper, successfully transitioning the study from a purely phenotypic description to a deeper mechanistic exploration of how C-terminal mutations specifically impair NtFD1 recruitment.

While the reliance on in planta BiFC and mcBiFC assays is justified given the reported stability issues of FT-like proteins in heterologous systems, the authors should more explicitly discuss the biochemical implications of the G170 residue. I suggest adding a focused discussion on whether this mutation alters the overall protein folding or specifically disrupts the 'docking' interface with 14-3-3 and NtFD1. Specifically, the authors should clarify if the NtFT5MM variant still maintains its capacity to form the initial 14-3-3 dimer bridge, as this would isolate the defect to the final recruitment of the FD transcription factor, thereby providing a 'smoking gun' for the Maryland Mammoth phenotype.

Version 2:

Reviewer comments:

Reviewer #1

(Remarks to the Author)
This manuscript is acceptable.

Reviewer #3

(Remarks to the Author)
After rounds of rebuttal, the manuscript is better than ever. I have no more questions on the current version.

Open Access This Peer Review File is licensed under a Creative Commons Attribution 4.0 International License, which permits use, sharing, adaptation, distribution and reproduction in any medium or format, as long as you give appropriate credit to the original author(s) and the source, provide a link to the Creative Commons license, and indicate if changes were made.
In cases where reviewers are anonymous, credit should be given to 'Anonymous Referee' and the source.
The images or other third party material in this Peer Review File are included in the article's Creative Commons license, unless indicated otherwise in a credit line to the material. If material is not included in the article's Creative Commons license and your intended use is not permitted by statutory regulation or exceeds the permitted use, you will need to obtain permission directly from the copyright holder.
To view a copy of this license, visit https://creativecommons.org/licenses/by/4.0/

Reviewers' comments with point-by point response:

Reviewer #1 (Remarks to the Author):

Major Concerns:

The manuscript by Lena Grundmann et al. presents a causal mutant allele responsible for the short-day-specific flowing of the Maryland Mammoth (MM) cultivar. The authors focus on the flowering activator FT5, comparing the wild-type FT5 and FT5MM for their endogenous expression profile, transgenic flowering photoperiod, and the role of FT5's C-terminus in its interaction with FD1 to promote flowering. They claim that FT5MM is the causal mutant allele for responsible for the MM short-day flowering.

The MM cultivar is instrumental in the discovery of photoperiodism in plants, but the gene affected by the Maryland Mammoth mutation is not known yet. Thanks to extensive research on Arabidopsis, the conserved photoperiod pathway is now relatively well-understood. While the activator FT5 is not a novel finding, the authors' identification its relationship to the extreme flowing phenotype of MM provides a crucial piece in solving a century-old puzzle.

This manuscript presents two main conclusions - that FT5 is the causal mutation and changes in its C-terminus affect its interaction with FD1. However, the paper is burdened with extraneous text that distracts from the main message. The manuscript lacks clear organization, and the evidence is not presented coherently. Specifically, the third section of the results is overly long, and difficult to follow.

Response:

Thank you for the detailed and constructive review. We appreciate both the recognition of the manuscript's strengths and the clear feedback regarding its structural weaknesses. We provide a point-by-point response to the comments below.

Detailed comments:

1. The third result section, "Functional characterization of NtFT5MM" is too long, and should be divided into several sub-sections for better readability.

Response:

We thank the reviewer for this helpful suggestion. We have now divided this section into several shorter subsections and added informative sub-headers. This restructuring improves the readability of the manuscript and emphasizes the sequential logic of the experiments.

2. L393, "To gain more insight into the mechanistic difference between NtFT5 and NtFT5MM", authors first perform BiFC to test the interaction between two FT5s and FD1, then shift to protein stability and protein transfer analyses, and later used multicolor BiFC to differentiate their interactions to FD1. This sequence is somewhat confusing, as the protein stability and transfer experiments divert attention from the key focus on the interaction analysis. These latter experiments should be removed or relocated.

Response:

We appreciate this insightful comment. We have restructured the entire section to present the experiments in a more coherent order. The revised section now begins with the analysis of protein stability and phloem transport, followed by the BiFC assays and the mcBiFC competition experiments. This order reflects the logical progression of the work: first excluding basic defects (stability, mobility) and then analyzing interaction specificity.

3. The key question regarding the FTMM's mutation at its C terminus is how it is associated to MM's short-day response. I noticed that the C-terminus contains several positive R, like a nucleus localization sequence (NLS). It is worth considering whether a deficient NLS could attenuate nucleus import, thereby reducing the FT5MM interaction with FD1 and impacting its function. After all, the flowering phenotypes of FT5MM W170G and NtFT5G170W are not

same as the NtFT5 transgenic lines (Fig 5g), which suggests other residues, aside from G170, may play important roles.

Response:

We thank the reviewer for raising this mechanistically important point. We now address this hypothesis in the discussion. Although the BiFC data show that both NtFT5 and $NtFT5_{MM}$ localize to the nucleus, we agree that additional C-terminal residues beyond G170 are important for FT/FD interaction and had already stated this in the manuscript (line 582). We have expanded the discussion to incorporate recent structural insights into FT–FD–DNA assembly as very recently reported by Gao et al. (2025) and explain how the C-terminal region mediates DNA-proximal docking and FAC incorporation. This supports the view that the lower activity of $NtFT5_{MM}$ reflects weakened FD1 recruitment rather than impaired nuclear import.

4. As interaction test between FT5 and FD1, other assays like Y2H can be included.

Response:

We appreciate the reviewer's suggestion to include additional interaction assays. We have performed several independent approaches to assess FT–FD interactions in tobacco, including Y2H, co-immunoprecipitation, and pulldown assays. We initially attempted to purify recombinant NtFT5 protein but were unable to obtain soluble protein from either E. coli or in vitro expression using the ALiCE kit (example shown in Review Fig. R1 at the end of this document).

<u>Y2H assays:</u>

Standard autoactivation controls confirmed that none of the BD–FT, BD–FD, or BD–14-3-3 constructs showed autoactivation on selective media (Fig. R2a). As validation, Arabidopsis proteins (AtFT, AtFD, AtGRF7) showed the expected interaction pattern: weak and orientation-dependent AtFT–AtFD interaction, strong AtFD–AtGRF7 interaction, and no AtFT–AtGRF7 interaction (Fig. R2b), fully consistent with the updated FAC model (Gao et al., 2025). Using tobacco proteins (NtFT5, NtFD1, multiple Nt14-3-3 isoforms, and NtFD1 phospho-variants; Fig. R2c), we observed robust NtFD1–Nt14-3-3 interactions but no stable NtFT5–NtFD1 or NtFT5–Nt14-3-3 interactions in any orientation. Phosphomimetic $NtFD1_{SEAPF}$ showed only minimal, and $NtFD1_{SAAPF}$ no detectable interaction with Nt14-3-3 isoforms. These observations indicate that NtFT5 does not form stable binary interactions in yeast and that FT recruitment requires a pre-assembled FD–14-3-3–DNA complex, which cannot be reconstructed in a Y2H system.

<u>CoIP and pulldown assays:</u>

To further evaluate FT5–FD1 interactions, we conducted CoIP experiments using His-NtFD1 and eGFP-NtFT5 (Fig. R3). Although all proteins were detectable in crude and nuclear extracts, eGFP-NtFT5 consistently appeared in elution fractions regardless of whether NtFD1 was co-expressed. To determine whether this unspecific signal originated from the GFP tag or from NtFT5 itself, we performed a control experiment using eGFP-tagged Strep protein. This control did not bind to Ni-INDIGO resin, demonstrating that the unspecific binding is attributable to NtFT5 rather than to the GFP tag. A similar limitation was evident in pulldown assays (Fig. R4–R5). When immobilized His-$NtFD1_{SEAPF}$ was incubated with extracts containing eGFP-NtFT5, faint FT5 signals were detected in elution fractions. Importantly, similarly faint signals were also detected in negative-control reactions lacking immobilized NtFD1, again indicating unspecific association of NtFT5 with Ni-INDIGO matrices.

Because both biochemical approaches were limited by NtFT5 binding non-specifically to the affinity matrix, and because all assays were performed in a heterologous system, further experiments in the appropriate endogenous context would be required. Such an approach, however, would exceed the scope of this revision in terms of time and content, making it a subject for future studies. We revised the manuscript to integrate recent insights into FT–FD–

DNA assembly from Gao et al. (2025) and now discuss our results within this mechanistic framework.

5. The FT7 data seems to be tangential and could be moved to the discussion section. PCR cannot definitively exclude its presence in MM, especially in the absence of amino acid and DNA sequence information.
Response:
We agree. The FT7 data have been removed because the results do not contribute to the core mechanistic conclusions.

6. The figure 1i) and 1j) is difficult to interpret the floating bars. It would be clearer to present FT4 and FT5 in separate plots.
Response:
We thank the reviewer for this helpful recommendation. FT4 and FT5 expression are now shown in separate panels with appropriate statistical analysis. This greatly improves the clarity of the data.

7. L479, ref "Beinecke et al, 2018)" is ref#18.
Response:
Corrected as requested.

8. L347, what is the source of P-NtFT5, from Hicks or MM? Comparing the two FT versions driven by the endogenous FT5MM promoter and their respective flowering photoperiods is crucial evidence for the claims.
Response:
We have clarified in the manuscript that the endogenous NtFT5 promoter is identical in SR1 and MM. This is now explicitly stated in line 390.

9. L356, why only a subset lines (3/10, 4/10, and 7/10) recover their long-day photoperiod?
Response:
We have stated in lines 404/405 "However, not all P-NtFT52.6kb:NtFT5//MM plants flowered, so the expression of NtFT5 driven by the 2.6-kb endogenous promoter does not fully restore native NtFT5 expression."

10. I do not see the method description of FLA analysis.
Response:
The method description is already present in the Methods section (163-172).

11. L400, which 15 amino acids are deleted in FT5*?
Response:
We have clarified that the deletions correspond to the last 15 amino acids (line 448) and added an alignment in Supplementary Fig. S6.

12. Why the WB signal in Fig S6 is not specific as in FigS5.
Response:
We thank the reviewer for this careful observation. We discovered that different antibodies were used inadvertently and corrected both the figure and the legend accordingly.

Reviewer #2 (Remarks to the Author):

This study provides a systematic elucidation of the molecular mechanism behind the non-flowering phenotype in the tobacco variety Maryland Mammoth (MM) under long-day conditions. It demonstrates that a 2-bp deletion in the key flowering activator NtFTS leads to a C-terminal truncation, which compromises its interaction with NtFD1 and ultimately impairs flowering induction. The research is methodologically sound and supported by a comprehensive dataset, underscoring its biological significance. However, there is potential for improvement in experimental logic, data presentation, and the depth of discussion.

Response:

Thank you for the positive evaluation of our work and for the constructive suggestions regarding experimental logic, data presentation, and discussion depth. We have carefully considered all comments and address them in detail in the point-by-point response below.

(1) Introduction

2.1 The historical background of the MM variety is somewhat lengthy and could be condensed.

Response:

We agree and have substantially condensed this section in the introduction.

2.2 The overview of FT/FD interactions in tobacco lacks systematic organization; incorporating recent advances in this area is recommended.

Response:

We have adjusted this part of the introduction to present a clearer, more structured overview.

2.3 The relationship between this study and prior work (e.g., Beinecke et al., 2018) should be explicitly articulated.

Response:

We now explicitly connect our study to Beinecke et al. (2018) and describe how our findings extend this prior work.

(2) Results

2.4 Fig. 1i–j: The correlation between NtFT4/5 expression trends and phenotypic changes is not immediately evident. Including a time-course expression heatmap along with statistical analysis of expression data would help clarify whether the observed differences are significant.

Response:

We thank the reviewer for this suggestion. We have separated the FT4 and FT5 expression datasets (as also requested by Reviewer 1), added statistical analyses, and provided the corresponding correlation analysis in Supplementary Data 1. Based on these additions, we consider the box-plot representation to be the most informative and interpretable format for highlighting temporal differences in expression. We therefore retained the box plots rather than adding a heat map, and we hope that the revised analyses and figures now present the expression trends with sufficient clarity.

2.5 Fig. 3a: The sequence alignment should highlight the specific mutation site and its potential location within the protein structure (e.g., within a functional domain).

Using AlphaFold2 to predict structural differences between NtFT5 and NtFT5MM is advisable.

Response:

We have improved the sequence alignment in Fig. 3a, marked the stop codon and mutation site, and added AlphaFold-based structural modeling (new Fig. 3b).

2.6 Grafting experiment (Fig. 5d): The current sample size (n = 3) is relatively small; increasing the number of biological replicates would strengthen the reliability of the conclusions.

Response:

We appreciate the reviewer's comment regarding the sample size in the grafting experiment. In principle, increasing the number of biological replicates is desirable. However, expanding this experiment within a realistic revision timeline is not feasible: the complete workflow, from sowing to the point at which flowering of the scions can be assessed, requires more than 6 months. In addition, generating further replicates is technically demanding because the different plant lines reach graft-compatible stem diameters at different times, which complicates the production of matched grafts.

Given this extended duration, the technical constraints, and the fact that the grafting assay provides supporting evidence for $FT5_{MM}$ mobility rather than forming the entire basis of our mechanistic conclusions, repeating the experiment would not yield a proportional scientific benefit. We have revised the manuscript to clarify that the grafting data provide additional evidence for the mobility of $NtFT5_{MM}$ (line 438) and retained the experiment because it independently corroborates the results of our stability, localization, and complementation assays.

2.7 Point mutation rescue experiment (Fig. 5f–g): While the results are clear, it would be beneficial to confirm whether the expression levels of the mutant protein are comparable to those of the wild type.

Response:

We have added a western blot (Supplementary Fig. S8c) and noted explicitly in the text that all corresponding proteins were immunodetected (lines 522/523).

2.8 Quantitative analysis of the binding affinity (e.g., KD values) between NtFT5/NtFT5MM and NtFD1 using methods such as yeast two-hybrid or surface plasmon resonance (SPR) is recommended.

Response:

We appreciate the reviewer's suggestion to include additional interaction assays. We have performed several independent approaches to assess FT–FD interactions in tobacco, including Y2H, co-immunoprecipitation, and pulldown assays. We initially attempted to purify recombinant NtFT5 protein but were unable to obtain soluble protein from either E. coli or in vitro expression using the ALiCE kit (example shown in Review Fig. R1 at the end of this document).

<u>Y2H assays:</u>

Standard autoactivation controls confirmed that none of the BD–FT, BD–FD, or BD–14-3-3 constructs showed autoactivation on selective media (Fig. R2a). As validation, Arabidopsis proteins (AtFT, AtFD, AtGRF7) showed the expected interaction pattern: weak and orientation-dependent AtFT–AtFD interaction, strong AtFD–AtGRF7 interaction, and no AtFT–AtGRF7 interaction (Fig. R2b), fully consistent with the updated FAC model (Gao et al., 2025). Using tobacco proteins (NtFT5, NtFD1, multiple Nt14-3-3 isoforms, and NtFD1 phospho-variants; Fig. R2c), we observed robust NtFD1–Nt14-3-3 interactions but no stable NtFT5–NtFD1 or NtFT5–Nt14-3-3 interactions in any orientation. Phosphomimetic $NtFD1_{SEAPF}$ showed only minimal, and $NtFD1_{SAAPF}$ no detectable interaction with Nt14-3-3 isoforms. These observations indicate that NtFT5 does not form stable binary interactions in yeast and that FT recruitment requires a pre-assembled FD–14-3-3–DNA complex, which cannot be reconstructed in a Y2H system.

<u>CoIP and pulldown assays:</u>

To further evaluate FT5–FD1 interactions, we conducted CoIP experiments using His-NtFD1 and eGFP-NtFT5 (Fig. R3). Although all proteins were detectable in crude and nuclear extracts,

eGFP-NtFT5 consistently appeared in elution fractions regardless of whether NtFD1 was co-expressed. To determine whether this unspecific signal originated from the GFP tag or from NtFT5 itself, we performed a control experiment using eGFP-tagged Strep protein. This control did not bind to Ni-INDIGO resin, demonstrating that the unspecific binding is attributable to NtFT5 rather than to the GFP tag. A similar limitation was evident in pulldown assays (Fig. R4–R5). When immobilized His-NtFD1$_{SEAPF}$ was incubated with extracts containing eGFP-NtFT5, faint FT5 signals were detected in elution fractions. Importantly, similarly faint signals were also detected in negative-control reactions lacking immobilized NtFD1, again indicating unspecific association of NtFT5 with Ni-INDIGO matrices.

Because both biochemical approaches were limited by NtFT5 binding non-specifically to the affinity matrix, and because all assays were performed in a heterologous system, further experiments in the appropriate endogenous context would be required. Such an approach, however, would exceed the scope of this revision in terms of time and content, making it a subject for future studies. We revised the manuscript to integrate recent insights into FT–FD–DNA assembly from Gao et al. (2025) and now discuss our results within this mechanistic framework.

2.9 Analyzing expression changes of downstream flowering-related genes (e.g., AP1/FUL homologs) in the shoot apical meristem (SAM) could help determine whether the FT mutation affects the expression intensity of downstream targets.

Response:

We appreciate the reviewer's thoughtful suggestion, which would indeed be informative for dissecting transcriptional cascades. However, our study is focused specifically on identifying and characterizing the molecular defect in FT5$_{MM}$ rather than mapping the downstream transcriptional network. Conducting meristem-level RNA profiling would require a separate experimental framework, including tightly staged time-series sampling across the floral transition, which is technically demanding and outside the scope of the current work.

Given these considerations, and the limited additional mechanistic insight such analyses would provide for the FT5-specific question addressed here, we consider this investigation beyond scope. We hope the reviewer agrees that the current dataset already offers a clear mechanistic explanation centered on the defective FT5 protein, while recognizing that a comprehensive analysis of downstream gene-expression dynamics would constitute a valuable follow-up study in its own right.

(3) Discussion

2.11 A deeper exploration is needed to explain why NtFTSMM still promotes flowering under short-day conditions, including an analysis of potential functional redundancy or synergy with other FT members (e.g., NtFT4).

Response:

We have expanded the last discussion paragraph to explain this, emphasizing functional redundancy and threshold effects involving NtFT4.

2.12 The discussion on how C-terminal truncation affects FD binding ability is insufficient. Incorporating existing structural knowledge of FT proteins to formulate reasonable hypotheses is recommended.

Response:

We have strengthened the structural interpretation of the C-terminal truncation by incorporating recent insights into FT–FD–DNA assembly and explicitly discussing how the DNA-contacting C-terminal residues contribute to FAC formation.

2.13 The article does not address whether NtFTSMM might indirectly regulate flowering through other signaling pathways (e.g., hormonal pathways).
2.14 The explanation for the delayed flowering of the MM variety under short-day conditions is inadequate. Considering the involvement of additional regulatory factors in this process is suggested.
Response to 2.13+2.14:
We have expanded the discussion (lines 604-611) and mention now that flowering in tobacco is strictly NtFT dependent, since NtFT5 knock-out plants do not flower under LD conditions and NtFT4/NtFT5 knock-out plants do not flower under SD conditions. Other signaling pathways are therefore not involved in flowering. We furthermore linked flowering of MM plants under SD to the partial functional redundancy between NtFT4 and $NtFT5_{MM}$.

(4) Language and Format
2.15 Some sentences are excessively long, impairing readability (e.g., lines 78–82). Splitting these into shorter sentences is recommended.
Response:
We have revised multiple sentences throughout the manuscript for clarity and readability.

2.16 Reference formatting is inconsistent; some journal names are abbreviated non-standardly (e.g., "Frontiers in Plant Sciences" should be "Front Plant Sci").
Response:
We have carefully standardized all references according to journal style.

Reviewer #3 (Remarks to the Author):

This manuscript addresses a classic, century-old question in plant biology the molecular basis for the short-day (SD) flowering requirement and long-day (LD) gigantism of the Nicotiana tabacum cv.'Maryland Mammoth (MM). The authors convincingly demonstrate that this phenotype is associated with a 2-bp deletion in NtFT5, the major LD floral activator. This deletion results in a frameshift and a truncated protein NtFT5MM that, while stable and mobile, functions as a weak floral inducer. Using genetic crosses, complementation assays, and protein interaction studies, the authors propose a model where the reduced binding affinity of NtFT5MM for the floral partner NtFD1 drops the total floral activation signal below the necessary threshold for flowering under LD conditions.

The work is scientifically sound, well-structured, and provides a highly satisfying solution to a long-standing mystery. The combination of genetic, molecular, and biochemical evidence is a significant strength. My recommendations below are intended to further strengthen the mechanistic claims of the manuscript.

Response:

Thank you for the positive evaluation and for the helpful recommendations to further strengthen the mechanistic conclusions. We have carefully considered all points and address them in detail in the point-by-point response below.

1. For the SR1 material, more relevant background should be provided for a better understanding of this work?

Response:

We now provide additional background on SR1 in the introduction (day-neutral behavior, in lines 75) and provided further clarification in the methods section (e.g., in lines 100/101).

2. The tight segregation of the homozygous NtFT5MM allele with the non-flowering phenotype in F2 crosses (Fig. 4) provides a clear genetic link.

Response:

We appreciate the reviewer's positive assessment of the segregation analysis. In the discussion, we describe the F_2 segregation pattern as showing a clear association between the homozygous $NtFT5_{MM}$ allele and the non-flowering phenotype.

3. For the gene expression levels of NtFT4 and NtFT5 in Fig. 1i and 1j, should statistical analysis be performed? For the the gene expression difference in one column such as NtFT5 in 6 WASS Hicks, why the difference is so large? Biological replicates performed or not?

Response:

We have included a statistical test and have updated the figure and legend accordingly. Biological replicates are clearly defined (line 239/240).

4. The last concern is the circadian gene and protein pattern of NtFT5 in MM and Hicks. Have ever been published? Just wonder if NtFT5 and NtFT5MM protein behave circadian rhythm.

Response:

We have clarified in the introduction (line 79/80) that NtFT genes do not show circadian rhythmicity in tobacco (Beinecke et al., 2018; Figure S9).

5. In the introduction, Hicks which is isogenic to MM except at the NtFT5 locus. Right? Not the MM locus? In the method, what are medial leaves?

Response:

We have clarified that Hicks and MM differ at the MM locus, now shown to be the $NtFT5_{MM}$ mutation (lines 95/96). We also defined "medial leaves" explicitly in the Methods in lines 104/105.

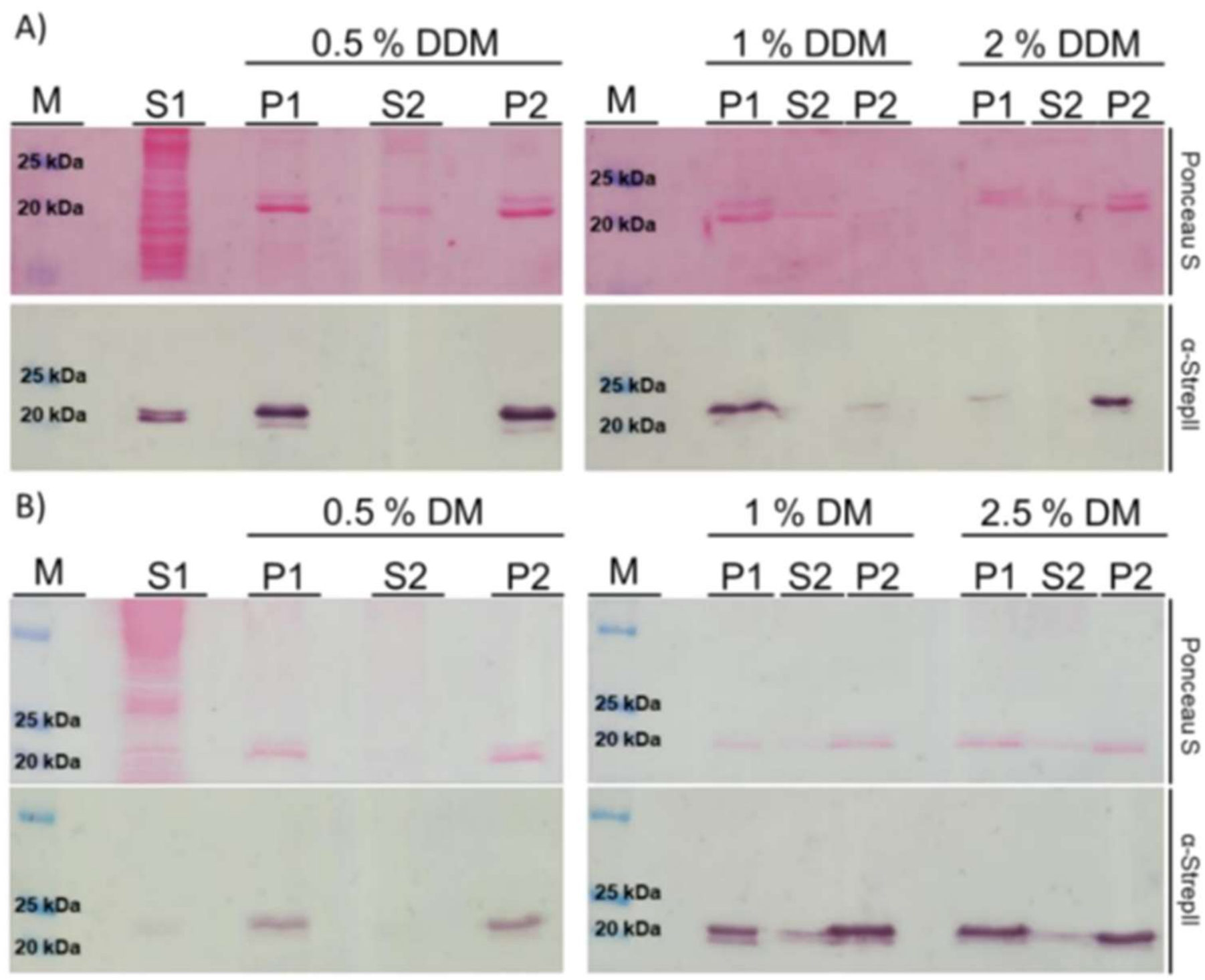

Figure R1: Purification of StrepII-NtFT5 expressed using the ALiCE kit from microsomes with different concentrations of DDM (A) and DM (B).

StrepII-NtFT5 (21 kDa) was expressed using the pAliCE02 vector. The purification of the recombinant protein from the microsomes includes three steps, from which four fraction are obtained: S1: cytosolic fraction without microsomes; P1: isolated microsomes before disruption; S2: Protein of interest; P2: microsome debris. After protein expression and purification from the microsomes, the proteins were separated by the SDS-Page and then transferred to a nitrocellulose membrane. The efficiency of the blot was checked by reversible Ponceau S staining. Different concentrations of DDM (A) and DM (B) were used to disrupt the microsomes, with the intention of obtaining the expressed protein in the soluble fraction (S2) at the end and not in the pellet fraction of the microsome debris (P2). α-StrepTag II antibodies (produced in mice) were used as the first antibody depending on the tag α-mouse IgG antibody conjugated with an alkaline phosphatase (produced in goats) were used as the second antibody. As reference, 4 µL of the Precision Plus Protein™ All Blue Marker was used (M).

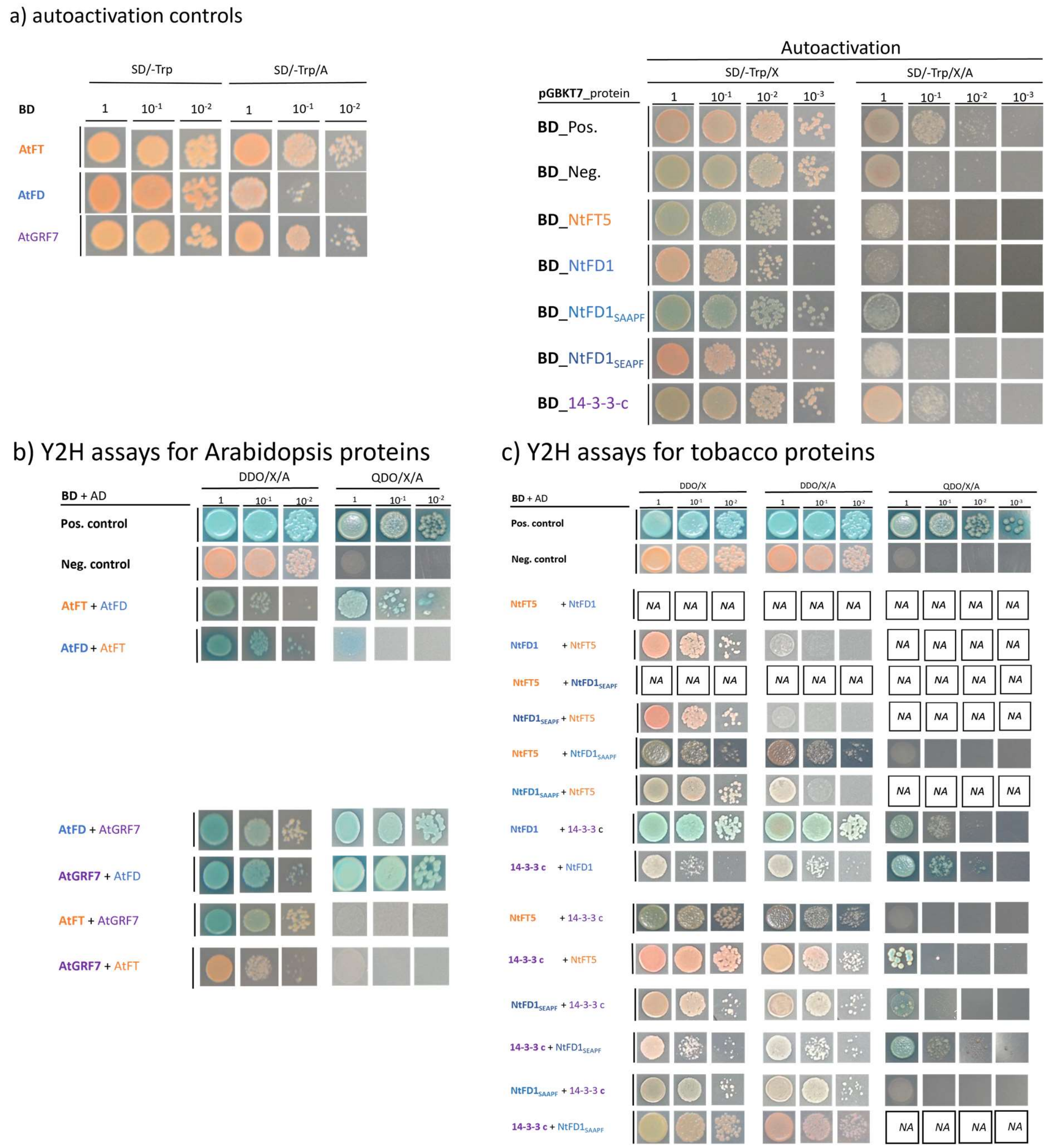

Fig. R2. Y2H analysis using FT, FD and 14-3-3 proteins from Arabidopsis (left) and tobacco (right). a) Standard autoactivation controls were conducted by expressing each BD–FT, BD–FD and BD-14-3-3 fusion on SD/–Trp plates supplemented with or without Aureobasidin A (400 ng/ml). All constructs showed growth on SD/–Trp (+ X-Gal), confirming successful transformation, but no or poor(er) growth on SD/–Trp (+ X-Gal) + Aureobasidin A. According to the manufacturer's criteria (Matchmaker manual, 2010), autoactivation is defined by strong growth and blue colony formation on SDO/X/A medium. As this was not observed for all BD-constructs, we concluded that none of our BD fusions exhibited autoactivation and that all constructs were suitable for subsequent interaction assays. **b)** As an initial validation, we tested Arabidopsis proteins (AtFT, AtFD, AtGRF7) and observed weak, orientation-dependent AtFT–AtFD signals, strong AtFD–AtGRF7 interactions, and no detectable AtFT–AtGRF7 interaction. **c)** We then tested tobacco NtFT5, NtFD1, Nt14-3-3s (three were tested, representative results shown for Nt14-3-3 c), and phospho-variants of NtFD1. No interaction was detected between BD–NtFT5 and AD–NtFD1, and only slight growth was observed in the reverse orientation on low-stringency medium (DDO/X/A), but not on QDO/X/A. Similarly, neither phospho-mimetic ($NtFD1_{SEAPF}$) nor non-phosphorylatable ($NtFD1_{SAAPF}$) variants clearly interacted with NtFT5. In contrast, NtFD1 showed a robust interaction with Nt14-3-3s in both directions, whereas NtFT5 did not interact with Nt14-3-3s, except for barely detectable background growth in one orientation. $NtFD1_{SEAPF}$ showed only minimal growth in any combination with Nt14-3-3s and, as expected, $NtFD1_{SAAPF}$ did not interact with Nt14-3-3s.

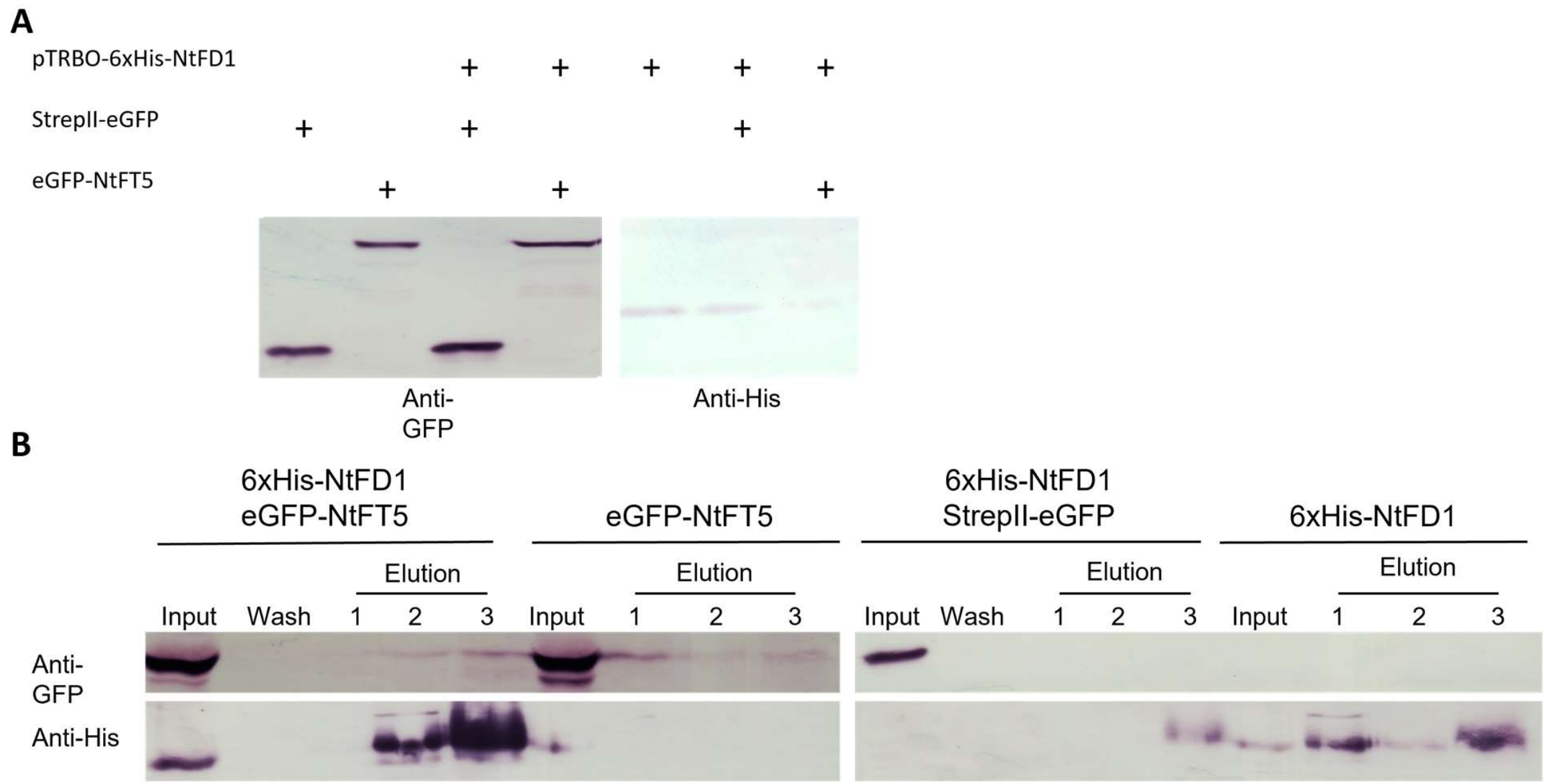

Fig. R3. CoIP of NtFT5 and NtFD1

A) Detection of eGFP-NtFT5, 6xHis-NtFD1 and StrepII-eGFP protein after Agroinfiltration in *N. benthamiana.* The full length coding sequence of *NtFD1* was fused to 6xHis Tag and inserted into the viral pTRBO vector, while *NtFT5* was fused to *eGFP* into the pLab12.10 vector. A StrepII-eGFP fusion, inserted in the pLab12.1 was used as a negativ control. *5*-week-old *N. benthamiana* leaves were injected with *A. tumefaciens* GV3101 PMP90*,* harboring the eGFP-NtFT5, 6xHis-NtFD1 and the StrepII-eGFP fusion constructs. The injected leaves were harvested 72 h after infiltration. For protein detection, crude proteins were extracted, by boiling 50 mg of leave material in 2x SDS loading buffer at 95°C for 10 min. The mixture was centrifuged at 4000 x g for 2 min and the protein containing supernatant was used for protein detection via Western Blot.

B) Co-Immunoprecipitation of eGFP-NtFT5 and 6xHis-NtFD1 using Ni-INDIGO magnetic-beads (CubeBiotech). 2 g of transiently transformed *N. benthamiana* leaves harboring the eGFP-NtFT5, 6xHis-NtFD1 and StrepII-eGFP proteins were crushed into a fine powder with liquid nitrogen, by mortar and pestle. The frozen material was lysed in 6 ml of Lysis buffer (50 mM Tris-HCl pH 7.5, 150 mM NaCl 10% Glycerol, 2 mM EDTA, 5 mM DTT, 0,5% Triton, 1:100 complete EDTA-free protease inhibitor tablets), by vortexing for 2 min. The suspension was squeezed through two layers of Miracloth. 1.5 ml of the supernatant was mixed with 100 µl of Ni-INDIGO Mag-beads and 5 mM of Imidazol was added to the beads to reduce unspecific binding of the beads. The samples were incubated for 1 h at 4°C while constant shaking. The beads were washed 4 times with wash buffer (50 mM Tris-HCl pH 7.5, 150 mM NaCl, 10% Glycerol, 2 mM EDTA, 20 mM Imidazole, 1:100 complete EDTA-free protease inhibitor tablets) and vortex vigorously. The proteins were eluted in total three times. The first two times, by adding 100 µl elution buffer (50 mM Tris-HCl pH 7.5, 150 mM NaCl, 10% Glycerol, 2 mM EDTA, 250 mM Imidazole, 1:100 complete EDTA-free protease inhibitor tablets) and vortex vigorously. In the last elution step the beads were boiled in 100 µl 2x SDS-loading buffer at 95°C for 10 min.

eGFP-NtFT5 and StrepII-eGFP was visuliazed by eGFP-specific primary antibody (GFP JL-8) and an alkaline phosphatase-labelled second antibody. For 6xHis-NtFD1 a His-specific primary antibody was used.

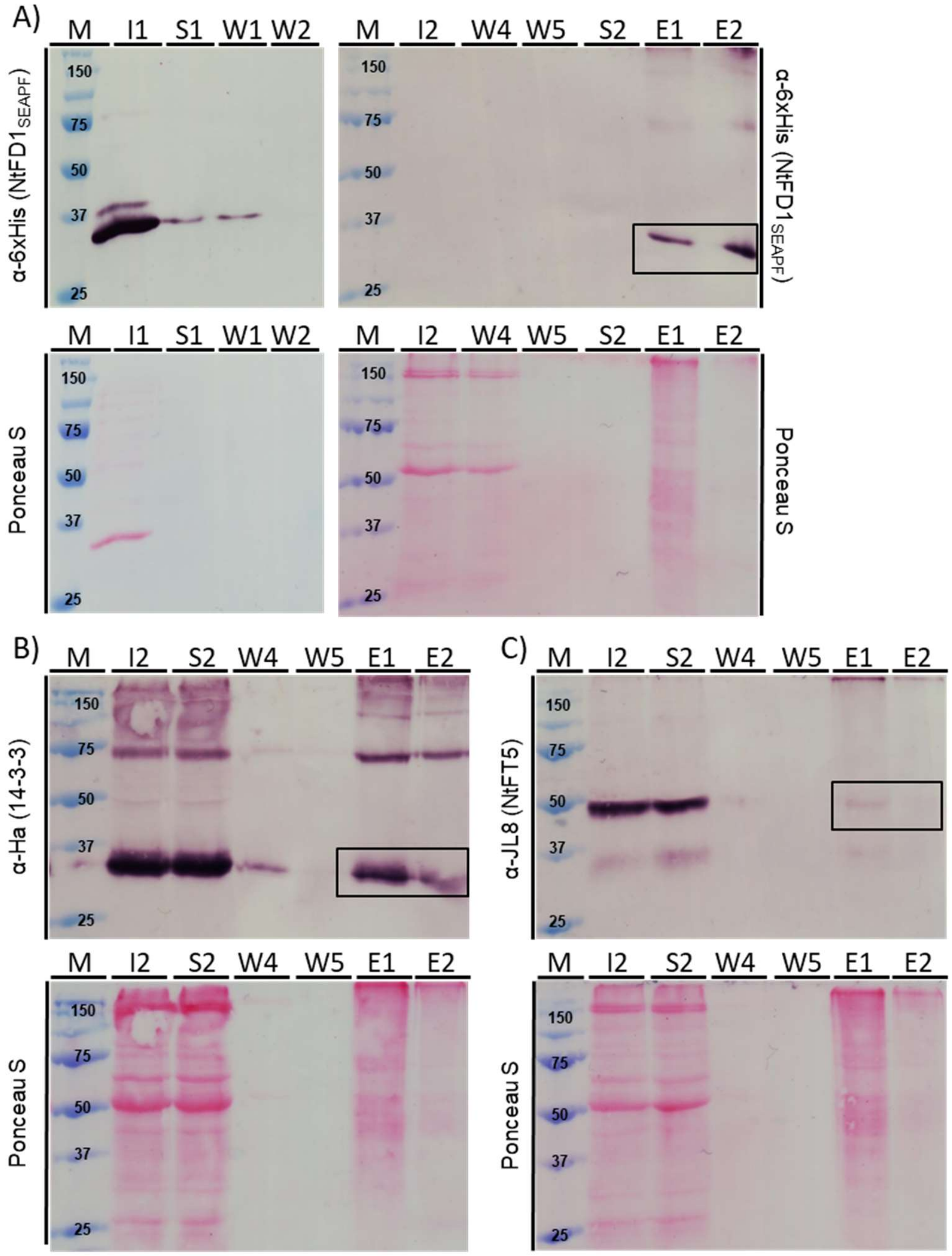

Fig. R4. Pulldown of eGFP-NtFT5 and HA-14-3-3c using 6xHis-NtFD1$_{SEAPF}$ bound to Ni-INDIGO MagBeads. 6xHis-NtFD1$_{SEAPF}$ (27 kDa) was expressed by ALiCE kit using the pALiCE01 vector (I1) and subsequently bound to Ni-INDIGO MagBeads. After several washing steps, the beads were incubated overnight with plant extract (I2) containing eGFP-NtFT5 (49 kDa) and HA-14-3-3c (30 kDa). This extract was previously produced using agrobacteria-mediated transformation. After further washing steps, elution was performed using imidazole. If interaction between NtFD1$_{SEAPF}$, NtFT5 and 14-3-3c has taken place, eGFP-NtFT5 and HA-14-3-3c should be detected here (black boxes). α-6xHistidine (A), α-HA (B) or α-JL8 (C) antibodies (produced in mouse) were used as the first antibody. The second antibody employed was α-mouse IgG antibody conjugated with an alkaline phosphatase (produced in goats). I = Input; S = Supernatant; W = Wash (W1/2: after first incubation, W4/5: after second incubation); E = Elution. 4 µL of Precision Plus Protein™ All Blue was used as a reference (M).

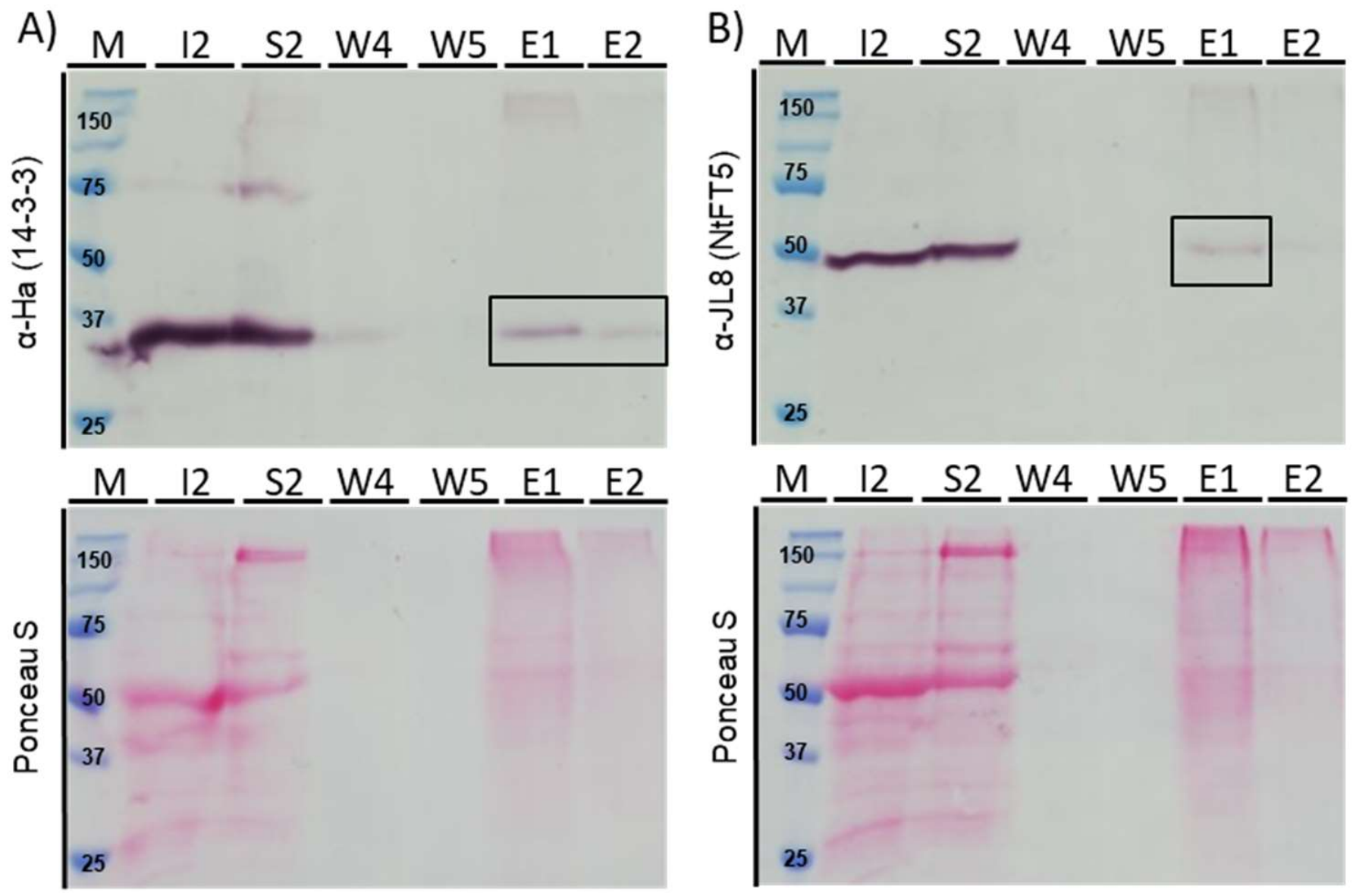

Fig. R5. Negative control of the pulldown of eGFP-NtFT5 and HA-14-3-3c using 6xHis-NtFD1SEAPF bound to Ni-INDIGO MagBeads. For a negative control, the plant extract (I2) containing eGFP-NtFT5 (49 kDa) and HA-14-3-3c (30 kDa) was incubated overnight with the beads without 6xHis-FD1$_{SEAPF}$ bound to these in advance. Ideally, neither HA-14-3-3c nor eGFP-NtFT5 should be detected in the elution in this set-up (black boxes), as there is no NtFD1$_{SEAPF}$ to interact with. α-HA or α-JL8 antibodies (produced in mouse) were used as the first antibody. The second antibody employed was α-mouse IgG antibody conjugated with an alkaline phosphatase (produced in goats). I = Input; S = Supernatant; W = Wash (W1/2: after first incubation, W4/5: after second incubation); E = Elution. 4 µL of Precision Plus Protein™ All Blue was used as a reference (M).

Reviewers' comments:

Reviewer #1 (Remarks to the Author):

1.1 The structure predicted using AlphaFold does not appear to provide additional mechanistic insight in its current form (Fig. 3a). Unless interaction interfaces or specific functional predictions are analyzed and discussed.

Response:

We have now augmented the discussion with a deeper analysis of the structural interactions predicted by AlphaFold 3 (lines 588–593; Fig. 7). FT surfaces that interact with 14-3-3 proteins adopt highly similar orientations and positions. The C-terminal residues of FT5 (R166, R172–174) mediate direct DNA contacts (as described for AtFT) that are required for recruitment to AtFD–14-3-3 and subsequent FAC assembly on target promoters (Gao et al. 2025). The altered C-terminal residues of FT5MM do not make contact with DNA, indicating that truncation and sequence alteration at the C-terminus can therefore restrict FAC assembly despite normal transcription, stability and transport. In line with the updated FAC model, strong overexpression of a C-terminally truncated and mutated FT variant, such as NtFT5MM, which has lost the C-terminal arginine cluster, cannot fully substitute for efficient recruitment to FD–14-3-3–DNA in the apex, explaining the partial complementation with delayed flowering.

1.2 The manuscript lacks sufficient background to justify why protein stability and transfer were examined. Neither the Introduction nor the Results section clearly explains the biological reasoning or hypothesis underlying these experiments.

Response:

We have added the following sentence to the Introduction (lines 62–65): "In addition to its tightly regulated transcription[3], FT is also subject to post-translational control, and specific mutations have been shown to alter FT protein stability or impair its transport to the SAM, demonstrating that these processes are essential for proper flowering, at least in Arabidopsis…[8,9]."

We have also emphasized this rationale in the Results. We now state: "…as previously reported for C-terminal FT mutants in Arabidopsis[8]." (lines 412/413) and, in the transport section (lines 432/433), we added: "…given that efficient FT transport is required for floral induction and certain FT mutants are known to be defective in long-distance mobility[9]."

These additions convey the biological motivation underlying the experiments more clearly.

1.3 The results of protein stability and transfer should be presented in two separate paragraphs.

Response:

As requested, we have separated these topics into two distinct sections and introduced a new subheading (line 430): "Assessment of NtFT5MM phloem mobility".

1.4 In Figure 6a, the protein level of FT5MM appears relatively weaker even at the 0 min time point. This raises the possibility that the protein may already be expressed at low levels or be intrinsically unstable prior to treatment.

Response:

The experiment shown in Fig. 6a was carried out using the pTRBO transient expression system in *N. benthamiana* leaf epidermal cells. Transient expression levels can vary between infiltrations, so

we repeated the experiment independently as already shown in Supplementary Fig. 4c,d, in which FT5 and FT5MM accumulate to similar levels at 0 min. Thus, although the reviewer's interpretation is reasonable for the particular experiment shown in Fig. 6a, the repetition indicates that FT5MM is not consistently expressed at lower levels and does not appear to be intrinsically unstable prior to treatment. We have now clarified this point in the revised manuscript and we have added the phrase "…two independent infiltrations in each case…" (line 422) to indicate that the experiment was repeated.

Reviewer #2 (Remarks to the Author):
This manuscript is acceptable, I have no further questions
Response:
We thank the reviewer for the helpful recommendations during the review process.

Reviewer #3 (Remarks to the Author):
The authors have significantly improved the clarity and logical flow of the manuscript by restructuring the Results section and partitioning the functional characterization of NtFT5MM into well-defined subsections. The reorganization of experimental data—placing protein stability and transport assays before the mechanistic dissection of the Florigen Activation Complex (FAC)—provides a much more coherent narrative that mirrors the biological progression of the florigen signal. Furthermore, the integration of recent structural insights from Gao et al. (2025) strengthens the theoretical framework of the paper, successfully transitioning the study from a purely phenotypic description to a deeper mechanistic exploration of how C-terminal mutations specifically impair NtFD1 recruitment.
Response:
We thank the reviewer for the helpful recommendations during the review process.

3.1 While the reliance on in planta BiFC and mcBiFC assays is justified given the reported stability issues of FT-like proteins in heterologous systems, the authors should more explicitly discuss the biochemical implications of the G170 residue. I suggest adding a focused discussion on whether this mutation alters the overall protein folding or specifically disrupts the 'docking' interface with 14-3-3 and NtFD1. Specifically, the authors should clarify if the NtFT5MM variant still maintains its capacity to form the initial 14-3-3 dimer bridge, as this would isolate the defect to the final recruitment of the FD transcription factor, thereby providing a 'smoking gun' for the Maryland Mammoth phenotype.
Response:
We have now augmented the discussion with a deeper analysis of the structural interactions predicted by AlphaFold 3 (lines 588–593; Fig. 7). We found that the residues mediating 14-3-3-dependent FT–FD interactions are conserved upstream of the 2-bp deletion in NtFT5MM. Furthermore, we propose that the presence of two tryptophan residues in FT5MM interferes with DNA-associated interactions of the FAC by reducing the net positive charge and introducing steric effects (lines 591–593). This may explain why substitution with glycine restores wild-type flowering behavior, whereas mutating the glycine residue to tryptophan in transgenic 35S:NtFT5G170W//SR1ΔNtFT5 lines abolishes the profound early flowering phenotype, probably due to steric constraints imposed by tryptophan at this position (lines 602–605).
