# Supplementary material for: A mutant allele of the long-day floral activator NtFT5 is associated with the short-day-specific flowering of tobacco cultivar Maryland Mammoth
Source: Commun Biol. 2026 Aug 26;9:1144. doi: 10.1038/s42003-026-10729-4 (PMC13507162; doi:10.1038/s42003-026-10729-4)
Supplement: Supplementary file 2 [file 42003_2026_10729_MOESM2_ESM.pdf]

Supplementary Information to

A mutant allele of the long-day floral activator NtFT5 is associated with the short-day-specific flowering of tobacco cultivar Maryland Mammoth

Lena Grundmann[1§], Marius M. Zimmermann[2§+], Christos Iordanidis[1], Andrea Känel[2], Axel Schwarze[2+], David R. Wiedmann[1+], Jost Muth[3], Richard M. Twyman[4], Dirk Prüfer[1,2] and Gundula A. Noll[1,2*]

[1]Fraunhofer Institute for Molecular Biology and Applied Ecology IME, Schlossplatz 8, 48143 Münster, Germany,

[2]University of Münster (UM), Institute of Plant Biology and Biotechnology, Schlossplatz 8, 48143 Münster, Germany,

[3]Fraunhofer Institute for Molecular Biology and Applied Ecology IME, Forckenbeckstraße 6, 52074 Aachen, Germany, and

[4]TRM Ltd, PO Box 493, Scarborough, YO11 9FJ, United Kingdom

[§]These authors contributed equally.

[*]For correspondence: Gundula A. Noll; Schlossplatz 8, 48143 Münster; Tel: +492518324998; gundula.noll@ime.fraunhofer.de

[+]M. Zimmermann, A. Schwarze and D. Wiedmann were affiliated with UM IBBP/Fraunhofer IME at the time the work was completed.

Fig. S1. Analysis of *NtFT5*$_{\text{Hicks}}$ (over-)expression in tobacco cultivars MM and Hicks by qPCR.
Fig. S2. Molecular and phenotypic analysis of SR1ΔNtFT5 and MM plants expressing *NtFT5* or *NtFT5*$_{\text{MM}}$.
Fig. S3. Detection of NtFT5 and NtFT5$_{\text{MM}}$ proteins by western blotting.
Fig. S4. Stability of NtFT5 and NtFT5$_{\text{MM}}$ proteins.
Fig. S5. BiFC analysis of NtFT5$_{\text{R61G}}$, NtFT5$_{\text{R129A}}$, NtFT5$_{\text{FNC*}}$ and AtFT with NtFD1, as well as NtFD3 and NtFD4 with NtFT5$_{\text{MM}}$, NtFT5, NtFT5$_{\text{FN*}}$ and AtFT.
Fig. S6. Western blot analysis of BiFC experiments with different mRFP-FT fusion proteins and NtFD1.
Fig. S7. Multicolor BiFC assay.
Fig. S8. Functional characterization of NtFT5$_{\text{MM W170G}}$ and NtFT5$_{\text{G170W}}$ mutants.
Fig. S9. Amino acid sequence alignment of FT-like and TFL-like proteins.
Table S1. Genotypes and phenotypes of F_2 plants.
Table S2. List of oligonucleotides used in this study.

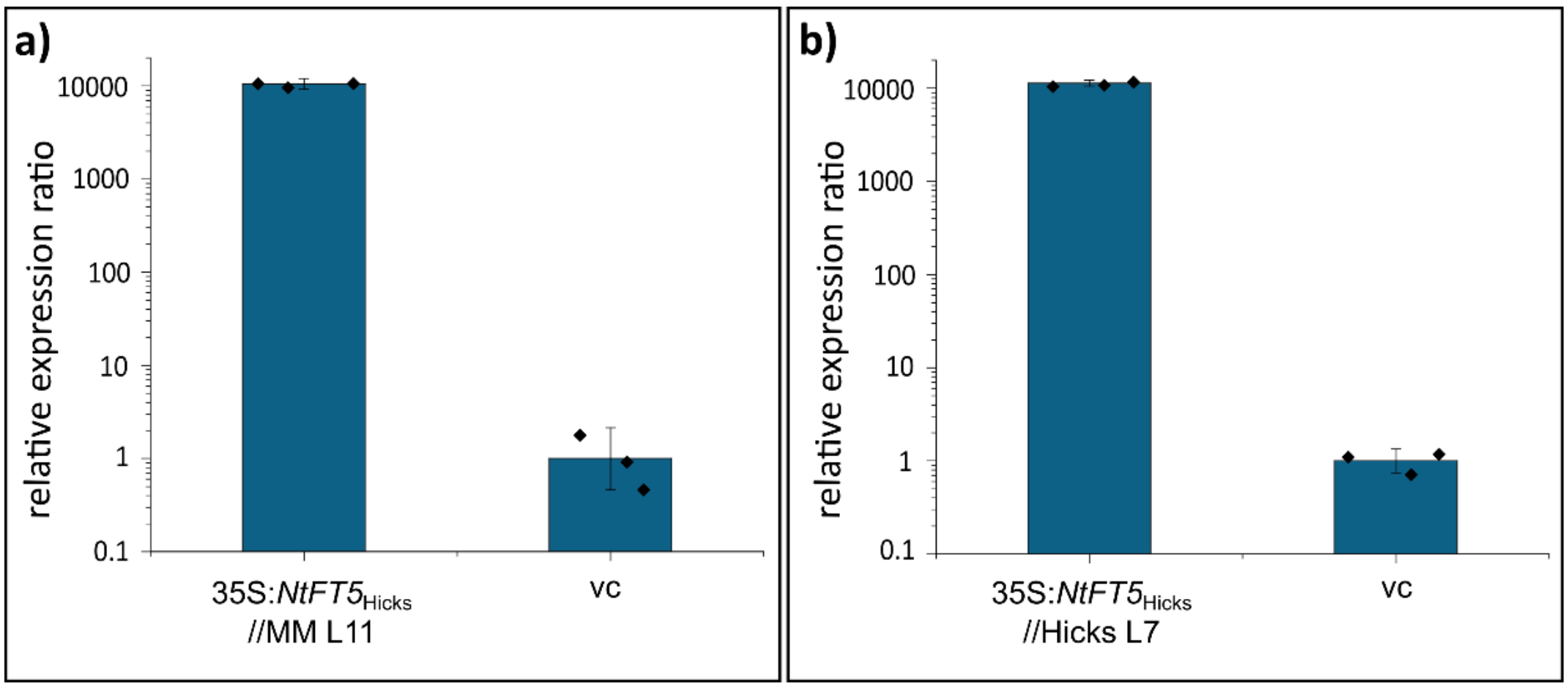

Figure S1. Analysis of *NtFT5*$_{\mathbf{Hicks}}$ (over-)expression in tobacco cultivars MM and Hicks by qPCR.
Samples were isolated from entire plants harvested at the tissue culture stage. Values were normalized to the reference gene *NtEF-1α*. Expression in the vector control (vc) was set to 1 and error bars represent the combined standard deviation of target and reference gene technical replicates. Technical replicates are shown as individual data points (diamonds). *NtFT5*$_{Hicks}$ mRNA levels were >10^4-fold higher in (**a**) MM L11 and (**b**) Hicks L7 transgenic plants than the corresponding vector controls.

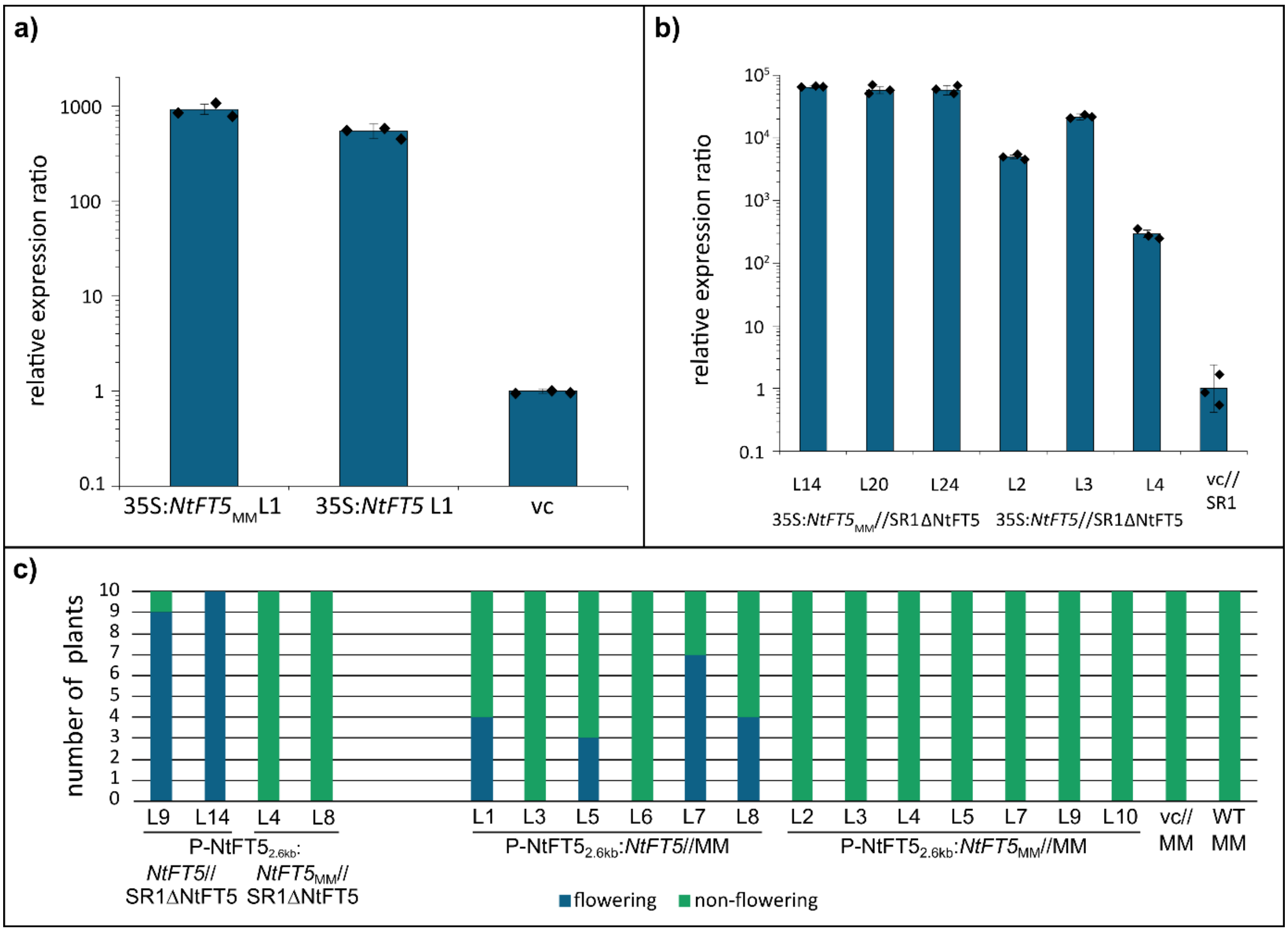

Figure S2. Molecular and phenotypic analysis of SR1ΔNtFT5 and MM plants expressing *NtFT5* or *NtFT5*$_{\mathbf{MM}}$.
a *NtFT5*$_{MM}$ and *NtFT5* expression was analyzed by qPCR using samples isolated from leaf material (*NtFT5*$_{MM}$) and the whole plant shoot for *NtFT5* and vector control (vc), both harvested in tissue culture. The values were normalized to the reference gene *NtEF-1α*. Expression in the vector control was set to 1 and error bars represent the combined standard deviation of the target and reference gene technical replicates. Technical replicates are shown as individual data points (diamonds). *NtFT5*$_{MM}$ mRNA levels (920-fold higher than vector control) and *NtFT5* mRNA levels (540-fold higher than vector control) strongly increased in both transgenic lines. **b** *NtFT5*$_{MM}$ and *NtFT5* expression was analyzed by qPCR using samples isolated from five pooled seedlings. The values were normalized to the reference gene *NtEF-1α*. Expression in the vector control was set to 1 and error bars represent the combined standard deviation of the target and reference gene technical replicates. Technical replicates are shown as individual data points (diamonds). The levels of *NtFT5*$_{MM}$ mRNA were higher in all lines compared to the vector control, ranging from 58,000-fold (L20 and L24) to 64,000-fold (L14) higher. *NtFT5* mRNA levels ranged from 300-fold (L4) to 22,000-fold (L3) higher than the vector control. **c** Number of flowering (blue) and non-flowering (green) plants for each line.

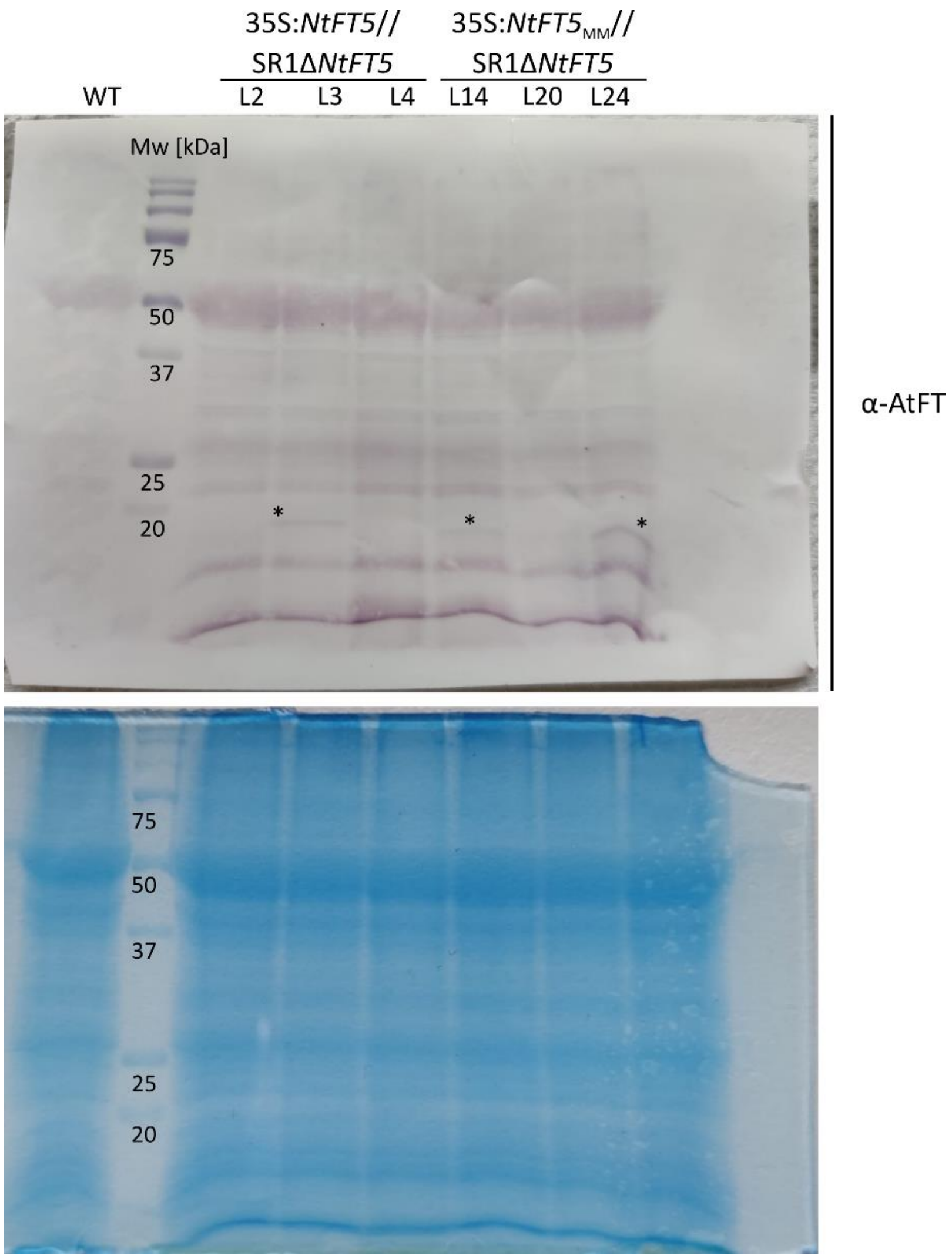

Figure S3. Detection of NtFT5 and $NtFT5_{MM}$ proteins by western blotting.
We loaded 15 µg of protein extracted from transgenic seedlings overexpressing NtFT5 and $NtFT5_{MM}$ in the SR1ΔNtFT5 background in each lane and detected the NtFT protein (*) with an AtFT-specific primary antibody and an alkaline phosphatase-labeled secondary antibody. Precision Plus Protein standards were used as size markers (Mw). Top panel shows the western blot. Bottom panel shows the polyacrylamide gel stained with Coomassie Brilliant Blue. Uncropped and unedited images of western blot and gel are shown.

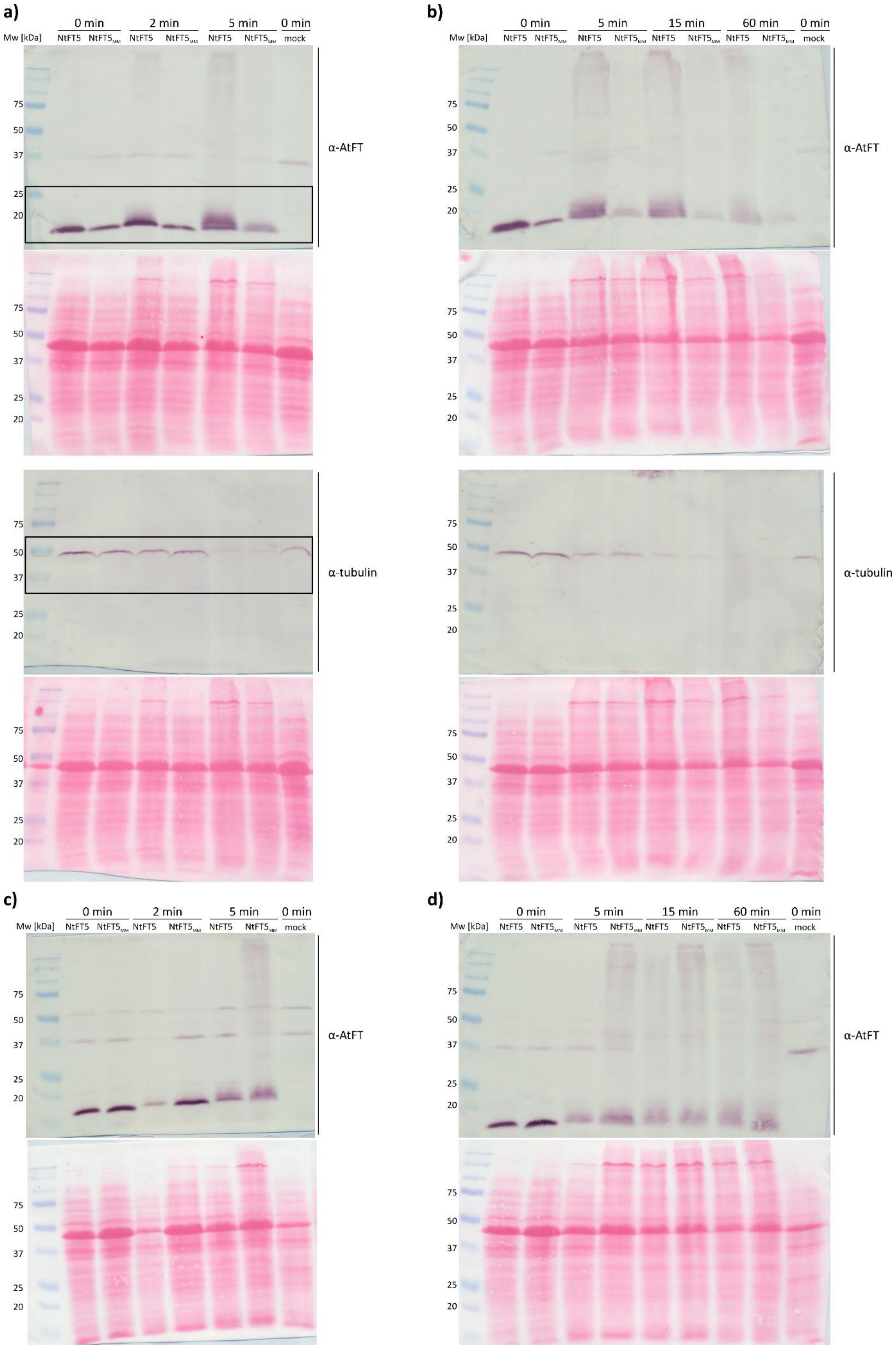

Figure S4. Stability of NtFT5 and NtFT5$_{MM}$ proteins.

NtFT5 and NtFT5$_{MM}$ protein stability was analyzed in leaf samples following transient expression in *N. benthamiana* using the pTRBO vector. Leaves were snap-frozen and 100 mg of each sample was incubated at room temperature for 0, 2, 5, 15 or 60 min. We then added 100 µl Laemmli buffer, vortexed the samples for 2 min, heated them to 100 °C, and separated them by SDS-PAGE (10 µl per lane) before western blotting. Proteins were detected using AtFT- and α-tubulin-specific primary antibodies and an alkaline phosphatase-labeled secondary antibody. Precision Plus Protein standards were used as size markers (Mw). Top panels show the western blot. Bottom panels show the membrane stained with Ponceau S as a loading control. **a** Uncropped image of western blots shown in Fig. 6a. **b** Further time intervals of same leaf material. **c,d** Repetition of the experiment shown in panels a and b.

NtFT5 FNCQRESGSGGRRRSAD.

NtFT5$_{FNC}$ FNC.

NtFT5$_{FN}$ FN.

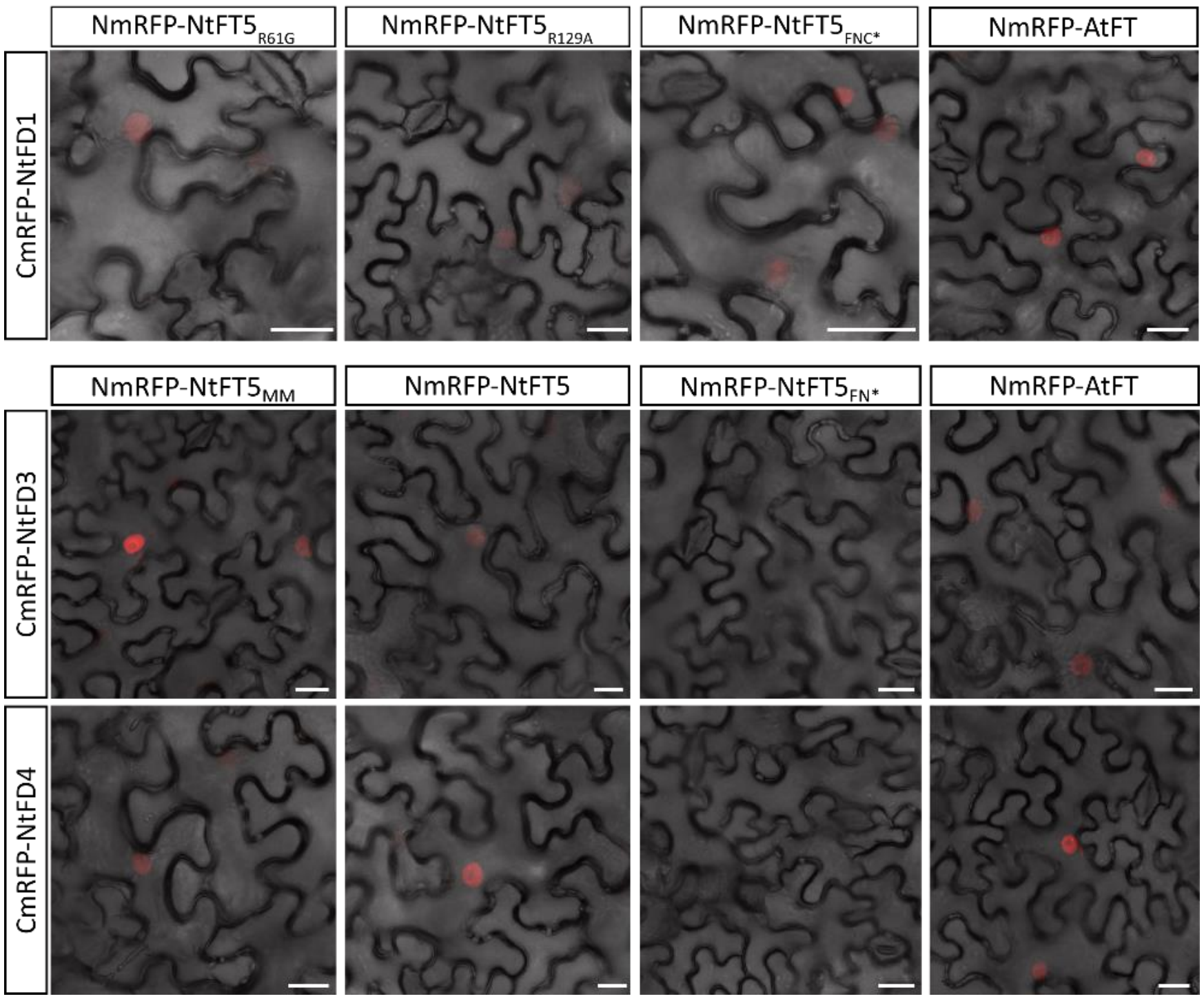

Figure S5. BiFC analysis of NtFT5$_{R61G}$, NtFT5$_{R129A}$, NtFT5$_{FNC*}$ and AtFT with NtFD1 as well as NtFD3 and NtFD4 with NtFT5$_{MM}$, NtFT5, NtFT5$_{FN*}$ and AtFT.

The 35S:*CmRFP-NtFD* constructs were co-expressed with 35S:*NmRFP* fusions of *NtFT5*$_{R61G}$, *NtFT5*$_{R129A}$, *NtFT5*$_{FNC*}$, *AtFT*, *NtFT5*$_{MM}$, *NtFT5* and *NtFT5*$_{FN*}$. BiFC analysis confirmed that NtFD1 interacts with NtFT5$_{R61G}$, NtFT5$_{R129A}$, NtFT5$_{FNC*}$ and AtFT, and also that both NtFD3 and NtFD4 interact with NtFT5$_{MM}$, NtFT5 and AtFT in *N. benthamiana* leaf epidermal cells. No interaction was detected for NtFT5$_{FN*}$ with NtFD1, NtFD3 or NtFD4. Scale bars = 25 µm.

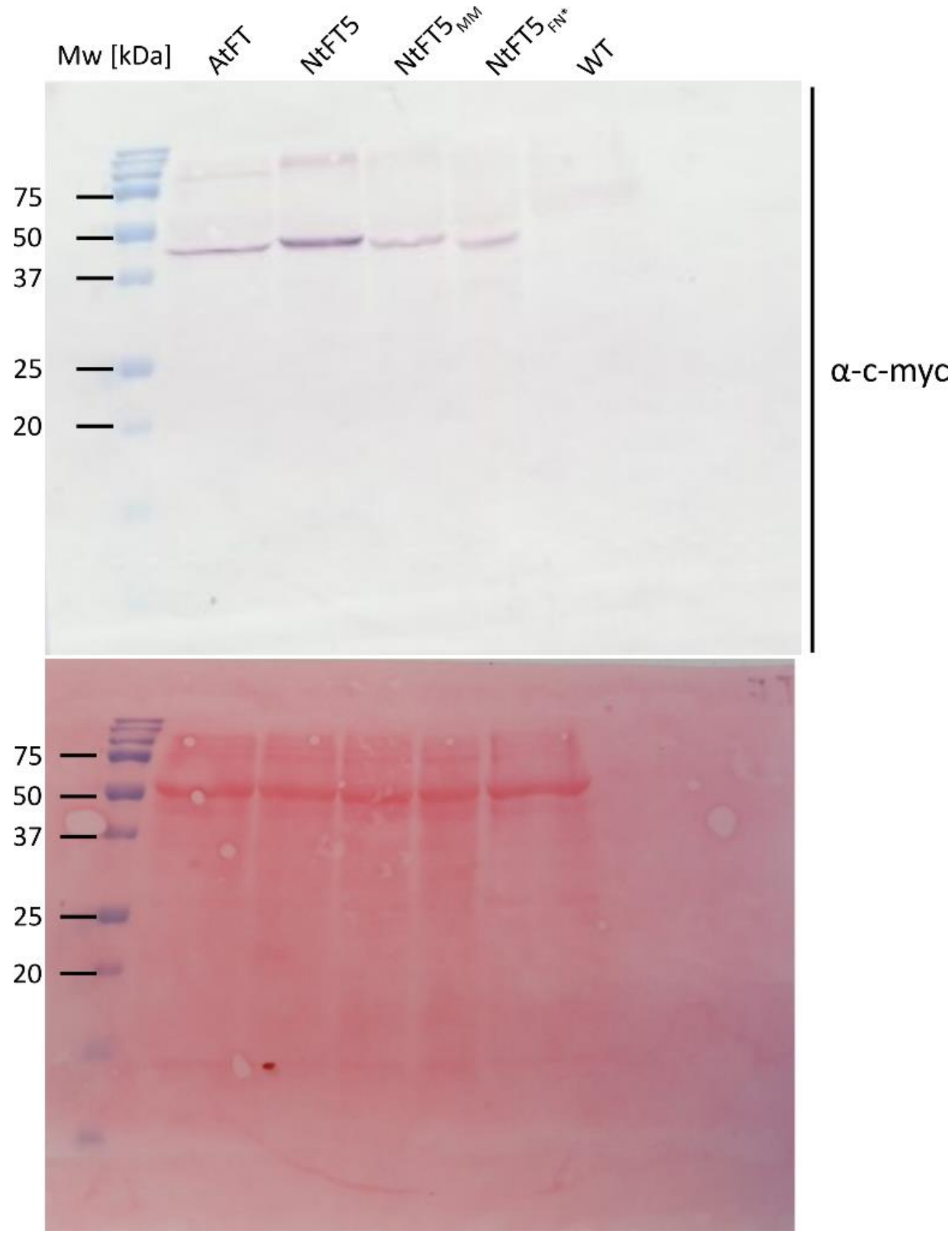

Figure S6. Western blot analysis of BiFC experiments with different mRFP-FT fusion proteins and NtFD1.
Protein extracts (12 µg per lane) were fractionated by SDS-PAGE, transferred to membranes and probed with a specific primary antibody against the c-Myc tag (9E10; Bio-Rad) diluted 1:500. We detected a band at the anticipated molecular weight for mRFP fusions of AtFT, NtFT5, $NtFT5_{MM}$ and $NtFT5_{FN*}$ in the BiFC experiments with NtFD1, indicating that all FT proteins are stable. The bands were visualized using an alkaline phosphatase-conjugated goat anti-mouse IgG secondary antibody (A3562; Sigma-Aldrich) diluted 1:10,000. Precision Plus Protein standards were used as size markers (Mw). Top panel shows the western blot. Bottom panel shows the membrane stained with Ponceau S as a loading control. Uncropped and unedited images of western blot and membrane are shown.

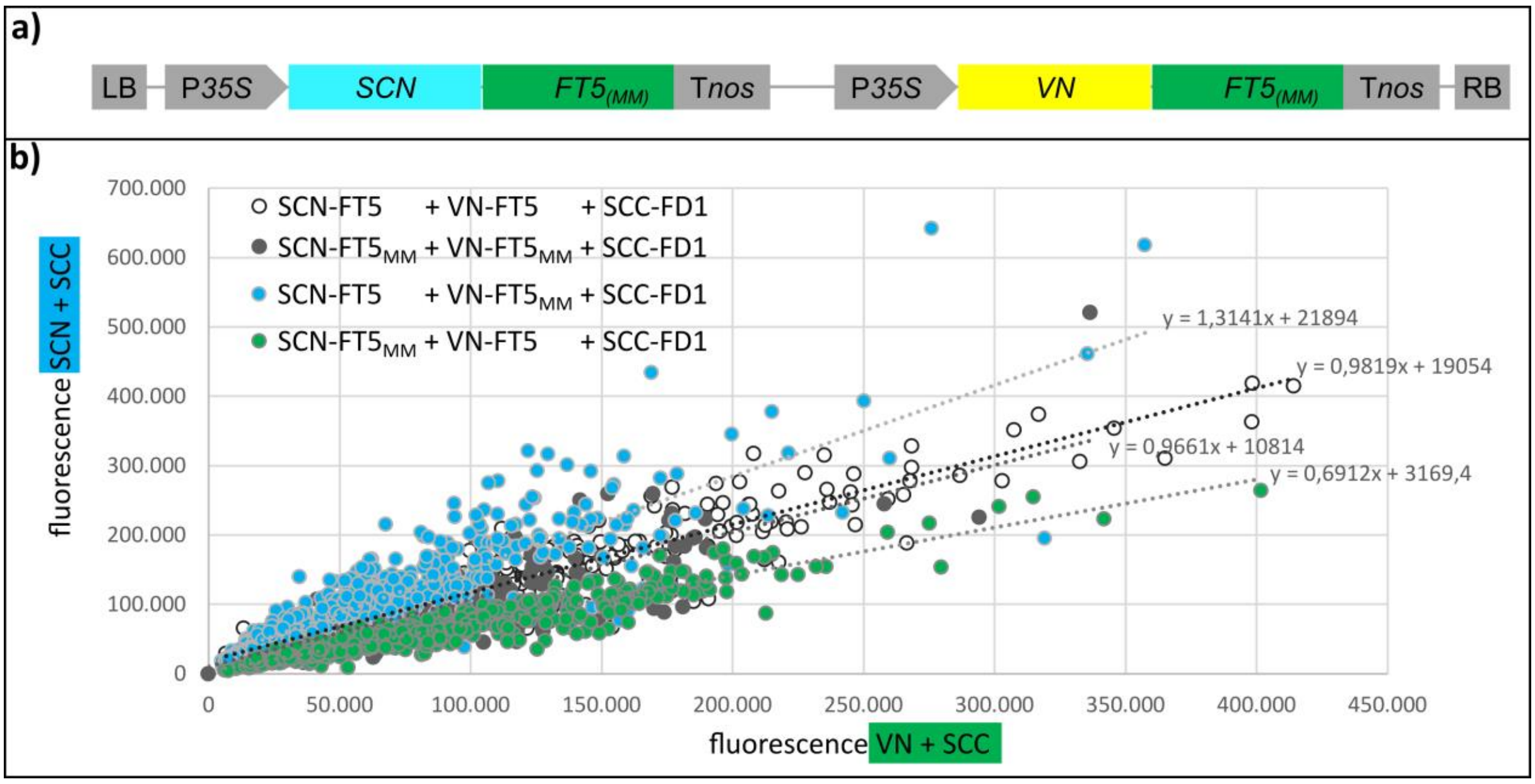

Figure S7. Multicolor BiFC (mcBiFC) assay.

a Schematic map of the T-DNA carrying two expression cassettes. **b** Analysis of different combinations of NtFT5 and $NtFT5_{MM}$ fusion proteins interacting with the SCC-NtFD1 fusion. Each dot represents a single nucleus. The *x*-axis indicates the strength of the VN+SCC signal, whereas the *y*-axis indicates the strength of the SCN+SCC signal. The combinations SCN-NtFT5 + VN-NtFT5 + SCC-NtFD1 (white, n=428) and SCN-$NtFT5_{MM}$ + VN-$NtFT5_{MM}$ + SCC-NtFD1 (black, n=341) show balanced competition. The combinations SCN-NtFT5 + VN-$NtFT5_{MM}$ + SCC-NtFD1 (blue, n=535) and SCN-$NtFT5_{MM}$ + VN-NtFT5 + SCC-NtFD1 (green, n=542) are both shifted towards the signal of the NtFT5-NtFD1 interaction. Abbreviations: SCN = N-terminal part of S(CFP)3A; VN = N-terminal part of Venus; SCC = C-terminal part of S(CFP)3A; P*35S* = CaMV 35S promoter; T*nos* = *A. tumefaciens nopaline synthase* terminator; LB = left border; RB = right border.

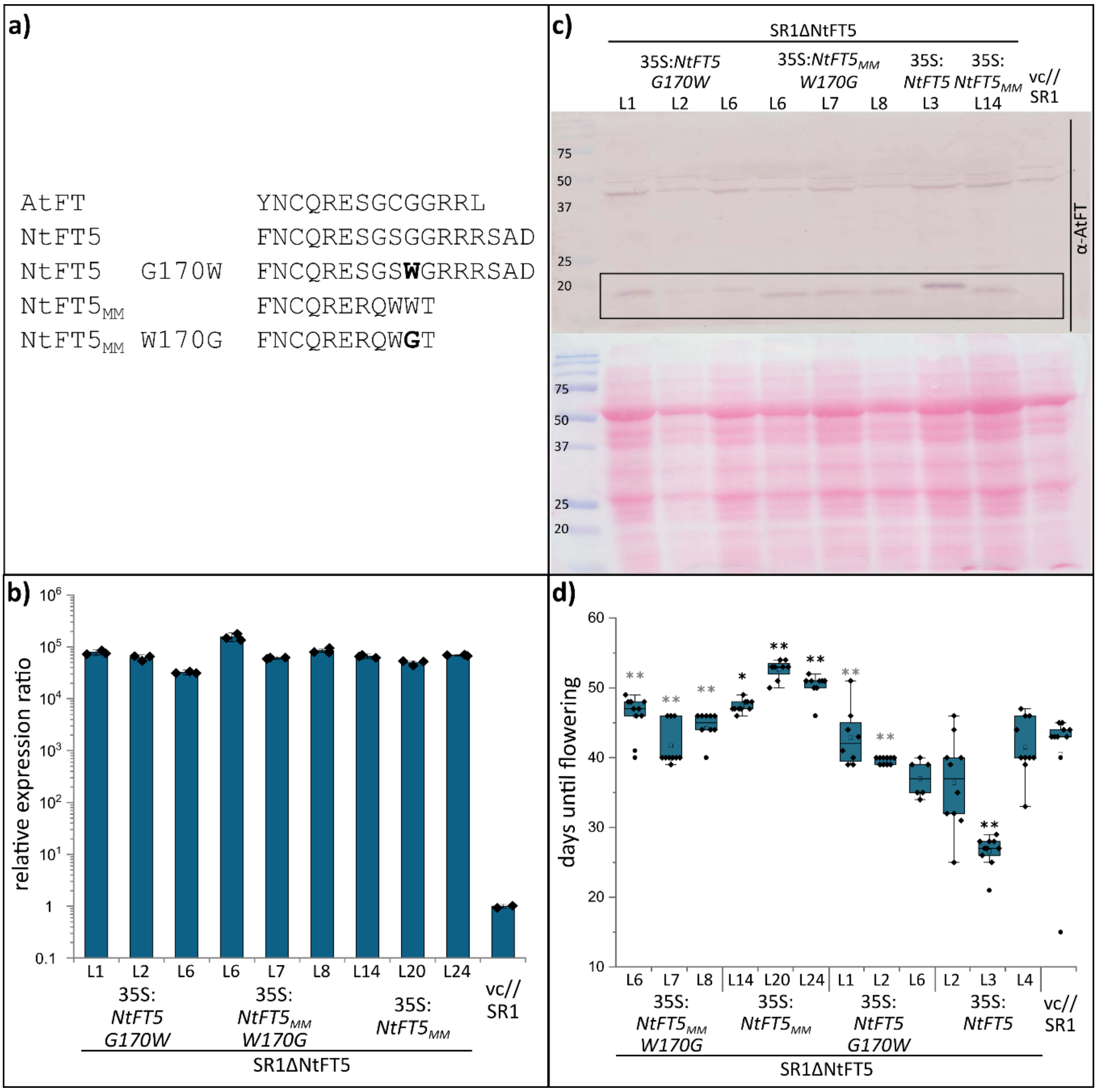

Figure S8. Functional characterization of NtFT5$_{MM\ W170G}$ and NtFT5$_{G170W}$ mutants.

a Partial amino acid sequence alignment of native NtFT5$_{(MM)}$, mutated NtFT5$_{(MM)}$ and AtFT. Amino acid substitutions are shown in bold. **b** Expression of native and mutated *NtFT5*$_{(MM)}$ was analyzed by qPCR using samples isolated from five pooled seedlings. The values were normalized to the reference gene *NtEF-1α*. Expression in the vector control was set to 1 and error bars represent the combined standard deviation of the target and reference gene technical replicates. The levels of *NtFT5*$_{MM}$ mRNA were 50,000-fold (L20), 66,000-fold (L14) and 69,000-fold (L24) higher than the vector control. The levels of *NtFT5*$_{G170W}$ mRNA were 32,000-fold (L6), 62,000-fold (L2) and 78,000-fold (L1) higher than the vector control. The levels of *NtFT5*$_{MM\ W170G}$ mRNA were 61,000-fold (L7), 84,000-fold (L8) and 154,000-fold (L6) higher than the vector control. **c** We loaded 45 µg of protein extracted from transgenic seedlings overexpressing NtFT5$_{MM\ W170G}$, NtFT5$_{G170W}$, NtFT5 or NtFT5$_{MM}$ in the SR1ΔNtFT5 background in each lane and detected the NtFT protein (*) with an AtFT-specific primary antibody and an alkaline phosphatase-labeled secondary antibody. Precision Plus Protein standards were used as size markers (Mw). Top panel shows the western blot. Bottom panel shows the membrane stained with Ponceau S as a loading control. Uncropped and unedited images of western blot and membrane are shown. **d** The number of days until flowering was defined as the period between seed sowing and the day the first flower opened. Strong overexpression of *NtFT5*$_{MM}$ in SR1Δ*NtFT5* plants resulted in delayed flowering in all transgenic lines compared to the SR1 vector control, whereas the overexpression of *NtFT5*$_{MM\ W170G}$ in SR1Δ*NtFT5* plants showed a significantly earlier flowering phenotype than 35S:*NtFT5*$_{MM}$//SR1Δ*NtFT5* lines. The lines overexpressing *NtFT5* showed an early flowering phenotype (significantly earlier in L3) when compared to the SR1 vector control, whereas NtFT5$_{G170W}$ lines flowered significantly later (L1 and L2) than 35S:*NtFT5*//SR1Δ*NtFT5* lines. Individual data points (diamonds) are shown for three 35S:*NtFT5*$_{MM}$//SR1Δ*NtFT5* lines (L14 and L24, n=10; L20, n=8), three 35S:*NtFT5*//SR1Δ*NtFT5* lines (L2, L3 and L4, n=10), three 35S:*NtFT5*$_{MM\ W170G}$//SR1Δ*NtFT5* lines (L6, L7 and L8, n=10), three 35S:*NtFT5*$_{G170W}$//SR1Δ*NtFT5* lines (L1, n=8; L2 n=10; L6, n=6) and one vector control line (n=10). Normal distribution was confirmed by applying the Kolmogorov-Smirnov test. Statistical significance was determined using a pairwise Welch's *t*-test with Bonferroni-Holm correction (**P<0.01, *P<0.05; black = significant *vs* vc; gray = significant *vs* native construct). In the boxplots, center line = median, square = mean, box limits = upper and lower quartiles, whiskers = 1.5× interquartile range, and circles = outliers.

```
                1                                                                                                                                                                              175
AtFT      ---MSI----NIRDPLIVSRVVGDVLDPFNRSITLKVTYGQR-EVTNGLDLRPSQVQNKPRVEIGGEDLRNFYTLVMVDPDVPSPSNPHLREYLHWLVTDIPATTGTTFGNEIVCYENPSPTAGIHRVVFILFRQLGRQTV-YAP---GWRQNFNTREFAEIYNLGLPVAAVFYNCQRESGCGGRRL---
NtFT4     ---MPR------IDPLIVGRVVGDVLDPFTRSVDLRVVYNNR-EVNNACGLKPSQIVTQPRVQIGGDDLRNFYTLVMVDPDAPSPSNPNLREYLHWLVTDIPATTDTSFGNEVICYENPQPSLGIHRFVFVLFRQLGRETV-YAP---GWRQNFSTRDFAEVYNLGLPVSAVYFNCHRESGTGGRRAY--
NtFT5     ---MPR-----EREPLVVGRVIGDVLDPFTRSIGLGVIYRDR-EVNNGCELRPSQVINQPRVEVGGDDLRTFYTLVMVDPDAPSPSDPNLREYLHWLVTDIPATTGASFGQEIVCYESPRPTMGIHRFVFVLFRQLGRQTV-YAP---GWRQNFNTKDFAELYNLGLPVAAVYFNCQRESGSGGRRRSAD
StSP6A    ---MPR------VDPLIVGRVIGDVLDPFTRSVDLRVVYNNK-DVNNACVLKPSQVVMQPRVHIGGDDLRNFYTLIMVDPDAPSPSDPNLREYLHWLVTDIPATTNTSFGNEVVCYENPTPTMGIHRFVLVLFRQSRRETV-YAP---GWRQNFNTRDFAELYNLGLPVAAVYFNSHRESGTGGRRA---
StSP3D    ---MPR-----ERDPLVVGRVVGDVLDPFTRTIGLRVIYRDR-EVNNGCELRPSQVVNQPRVEVGGEDLRTFFTLVMVDPDAPSPSDPNLREYLHWLVTDIPATTGSSFGQEIVSYESPRPSMGIHRFVFVLFRQLGRQTV-YAP---GWRQNFNTRDFAELYNLGLPVAAVYFNCQRETGSGGRRRSAD
SlSP3D    ---MPR-----ERDPLVVGRVVGDVLDPFTRTIGLRVIYRDR-EVNNGCELRPSQVINQPRVEVGGDDLRTFFTLVMVDPDAPSPSDPNLREYLHWLVTDIPATTGSSFGQEIVSYESPRPSMGIHRFVFVLFRQLGRQTV-YAP---GWRQNFNTRDFAELYNLGLPVAAVYFNCQRESGSGGRRRSAD
OsHd3a    MAGSGR-----DRDPLVVGRVVGDVLDAFVRSTNLKVTYGSK-TVSNGCELKPSMVTHQPRVEVGGNDMRTFYTLVMVDPDAPSPSDPNLREYLHWLVTDIPGTTAASFGQEVMCYESPRPTMGIHRLVFVLFQQLGRQTV-YAP---GWRQNFNTKDFAELYNLGSPVAAVYFNCQREAGSGGRRVYP-
GmFT2a    ---MPG----GSRNPLVVGRVIGEVIDPFEISIPFRVTYGNR-EVGNGCELKPSQVANQPRVSVGGDDLRNFYTMVLVDPDAPSPSNPNFREYLHWLVTDIPETTGPNFGNEVVSYESPRPTMGIHRLVFVLFRQQFRQRV-YAP---GWRQNFNTREFAELYNLGLPVAAVFFNCQRESGSGGRTF---
GmFT5a    ---MAR------ENPLVIGGVIGDVLNPFTSSVSLTVSINNR-AISNGLELRPSQVVNRPRVTVGGEDLRTFYTLVMVDADAPSPSNPVLREYLHWMVTDIPATTNASFGREVVFYESPNPSVGIHRIVFVLFQQLGRDTV-ITP---EWRHNFNSRNFAEINNLA-PVAAAYANCQRERGCGGRRY---
MtFTa1    ---MAG----SSRNPLAVGRVIGDVIDSFENSIPLRVTYGNR-DVNNGCELKPSQIGNQPRVSVGGNDLRNLYTLVMVDPDSPSPSNPTFKEYLHWLVTDIPGTTEVTFGNEVVNYERPRPTSGIHRFVFVLFRQQCRQRV-YAP---GWRQNFNTREFAELYNLGSPVAAVFFNCQRESGSGGRTFR--
PsFTa1    ---MAG----SSRNPLAVGRVIGDVIDPFENSVPLRVTYGSR-DVNNGCELKPSHVGNQPRVNVGGNDLRNIYTLVLVDPDSPSPSNPTFREYLHWLVTDIPATTEVSFGNEIVSYERPRPTSGIHRFVFILFRQQCRQRV-YAP---GWRQNFNTREFAELYNLGSPVAAVFFNCQRESGSGGRTFR--
BvFT2     ---MPR----APRDPLVVGRVIGDVLDPFSRTVNLRVSYSNR-DVNNGCELRPSQVVNQPRVEVGGDDLRTFYTLVMVDPDAPSPSNPHLREYLHWLVTDIPGTTGASFGQEVVCYENPRPSVGIHRFILVLFRQLGRQTV-YAP---GWRQNFNTRDFAELYNLGLPVAAVYFNCQREGGSGGRRL---
NtFT1     ---MSR------LDPLIVSGVIGDVLDSFTRSIDFSVVYNNRVQVYNGCGLRPSQIVNQPRVDIGGDDLRTFYTMVMVDPDAPTPSNPNLREYLHWLVTDIPATTGANFGNEIIRYESPRPSLGIHRYIFVLFQQLDREVV-NAPDIIDSRQNFNTRDFARFHNLNSPVAAVYFNCNREGGTGGRHL---
NtFT2     ---MLR------ANPLVVSGVIGDVLDPFTKSVDFDVVYNNNVQVYNGCGLRPSQIVNQPRVDIAGDDFRTFYTLVMVDPDAPTPSNPNLREYLHWLVTDIPATTEATFGNEIVSYERPQPSLGIHRYIFVLFRQLDREVV-NAPDIIDSREIFNTRDFARFHGLNLPVAAVYFNCNREGGTGGRHL---
NtFT3     ---MSR------LDPLIVSGVIGDVLDPFTRSIDFNVVYNNRMQVYNGCGLRPSQIVHQPRVDVGGDDLRTFYTLVMVDPDAPTPSNPNQREYLHWLVTNIPATTGAHFGNEIIQYESPRPSLGIHRYIFVLFRQLTRDVV-NAPDIIDSRENFNTRDFARFYDLNSPVAAMYFNSNRESGTGGRHL---
NtFT8     -MKMAR-----GRDTLVLGRVIGDVLDPFTRSIDLRVVYNSR-EVINGCDFRPSQVIHQPRIEIGGADACNFYTLVMVDPDAPTPTNPNMREYLHWLVTDIPATTGVNFGNEVVSYESPRPTIGIHRFVFVLFLQLRRENNVYAP---QSRQNFNTRDFAQLHNLGLPTAAVYFNGQRENGTGGRRL---
NtFT10    ---MQR-----GRDTLSLARVIGDVLDPFTKSISPRVFYNTR-EVKNGCDLRPSHVNNQPRVEVGGDDLRTFYTLVMVDPDAPTPSNPHQKEYLHWLVTDIPATTGVSFGNEVVSYECPRPTMGIHRLVLVLFRQSRRESV-YAP---ESRENFNTRDFAKHYNLGLPVAALYFNCQRENGTGGRRLI--
BvFT1     ---MPRTSASAPRDPLVLGGVIGDVLEPFERSVTLKISFNNR-NVNNGGDFRPSQVVNQPRVEVGGDDLRTCYTLVMVDPDAPSPSNPHQREYLHWLVTDIPGTTSASFGEEIVYYENPRPSTGIHRFVFALFRQLGRQTV-NAP---QQRQNFNTRDFAELYNLGLPVAAVYFNCQREGGCGGRRF---
AtTFL1    MENMGT----RVIEPLIMGRVVGDVLDFFTPTTKMNVSYNKK-QVSNGHELFPSSVSSKPRVEIHGGDLRSFFTLVMIDPDVPGPSDPFLKEHLHWIVTNIPGTTDATFGKEVVSYELPRPSIGIHRFVFVLFRQKQRRVI-FPN--IPSRDHFNTRKFAVEYDLGLPVAAVFFNAQRETAA--RRR---
NtCET1    ---MAS----RVVEPLVVARVIGEVVDSFNPSVKLNVIYNGSKQVFNGHELMPAVIAAKPRVEIGGEDMRSAYTLIMTDPDVPGPSDPYLREHLHWIVTDIPGSTDSSFGREIVSYESPKPVIGIHRYVLLLYKQSGRQTV-KPA---ATRDHFNTRRYTAENGLGSPVAAVYFNAQRETAA--RRR---
NtCET2    ---MGS----KMSDPLVIGRVIGEVVDYFTPSVKMSVTYNSSKHVYNGHELFPSSVTSKPRVEVHGGDLRSFFTMIMIDPDVPGPSDPYLREHLHWIVTDIPGTTDCSFGKEIVGYEMPRPNIGIHRFVFLLFKQKKRQTVLTAP---LSRDRFNTRKFAEENELGSPVAAVFFNCQRETAA--RRR---
NtCET4    ---MGS----KMSDPLVIGRVIGEVVDYFTPSVKMSVTYNSSKHVYNGHELFPSSVTSKPRVEVHGGDLRSFFTLIMIDPDVPGPSDPYLREHLHWIVTDIPGTTDCSFGREIVGYEMPRPNIGIHRFVFLLFKQKKRQTLLSAP---LSRDRFNTRKFSEENELGSPVAAAFFNCQRETAA--RRR---
StTFL1    ---MAS----KMCEPLVIGRVIGEVVDNFCPSVKMSVVYNNNKHVYNGHEFFPSSVTSKPRVEVHGGDLRTFFTLVMIDPDVPGPSDPYLREHLHWIVTDIPGTTDCSFGREVVGYEMPRPNIGIHRFVFLLFKQKKRQTISSAP---VSRDQFSSRKFSEENELGSPVAAVFFNCQRETAA--RRR---
OsRCN1    ---MSR-----SVEPLVVGRVIGEVLDTFNPCMKMIVTYNSNKLVFNGHELYPSAVVSKPRVEVQGGDLRSFFTLVMTDPDVPGPSDPYLREHLHWIVTDIPGTTDASFGREVISYESPKPNIGIHRFIFVLFKQKRRQTV-IVP---SFRDHFNTRRFAEENDLGLPVAAVYFNAQRETAA--RRR---
OsRCN2    ---MSR-----VLEPLIVGKVIGEVLDNFNPTVKMTATYGANKQVFNGHEFFPSAVAGKPRVEVQGGDLRSFFTLVMTDPDVPGPSDPYLREHLHWIVTDIPGTTDASFGREVVSYESPRPNIGIHRFILVLFRQKRRQAV-SPP---PSRDRFSTRQFAEDNDLGLPVAAVYFNAQRETAA--RRR---
GmTFL1a   ---MAR----MPLEPLIVGRVIGEVLDSFTTSTKMIVSYNKN-QVYNGHELFPSTVNTKPKVEIEGGDMRSFFTLIMTDPDVPGPSDPYLREHLHWIVTDIPGTTDATFGKELVSYEIPKPNIGIHRFVFVLFKQKRRQCV-TPP---TSRDHFNTRKFAAENDLALPVAAVYFNAQRETAA--RRR---
PsTFL1a   ---MAR----MAQEPLIVGRVIGEVLDSFTTSMKMTVSYNKK-QVFNGHEFFPSTINTKPKVEIDGADMRSFYTLVMTDPDVPGPSDPYLREHLHWIVTDIPGTTDATFGKEIVSYEIPKPNIGIHRFVFVLFKQRARDSVRATP---SSRDHFNTRSFASQNDLGLPVAAVYFNAQRETAA--RRR---
                         :.* :. *:*:*:: * : *. : *: : .*.: : * * . :*::: *.* * *::* .*:***:**:** :* ** *:: ** * * **** :: *: * * * *.:. :: * *.:* : * :** . *
```

Figure S9. Amino acid sequence alignment of FT-like and TFL-like proteins.
The glycine residue (highlighted in blue) at position 171 in AtFT is conserved in other FT-like proteins that act as floral activators (green) and repressors (red) in various plant species. TFL-like proteins (black) do not feature a glycine residue at this position. In Arabidopsis, the three arginine residues R166/R173/R174 (highlighted in yellow) mediate direct DNA contacts within the FD–14-3-3–DNA complex, enabling recruitment of AtFT to the florigen activation complex[1]. Accession numbers: *Arabidopsis thaliana* AtFT[2] (NM_105222), AtTFL1[3] (NM_120465); tobacco (*Nicotiana tabacum*) NtFT1[4] (JX679067), NtFT2[4] (JX679068), NtFT3[4] (JX679069), NtFT4[4] (JX679070), NtFT5[5] (KY306470), NtFT8[5] (KY306476), NtFT10[5] (KY306478), NtCET1[6] (AF145259), NtCET2[6] (AF145260), NtCET4[6] (AF145261); potato (*Solanum tuberosum*) StSP3D[7] (BAV67096), StSP6A[7] (BAV67095), StTFL1[8] (ABC24691); tomato (*Solanum lycopersicum*) SlSP3D[9] (AY186735); rice (*Oryza sativa*) OsHd3a[10] (NM_001063395), OsRNC1[11] (AP014967; LOC_Os11g05470.1), OsRNC2[11] (AP014958; LOC_Os02g32950.1); soybean (*Glycine max*) GmFT2a[12] (AB550122; Glyma16g26660), GmFT5a[12] (AB550126; Glyma_16G044100), GmTFL1a[13] (AB511821); *Medicago truncatula* MtFTa1[14] (HQ721813); pea (*Pisum sativum*) PsFTa1[15] (HQ538822) PsTFL1a[16] (AY340579); sugar beet (*Beta vulgaris*) BvFT1[17] (HM448910), BvFT2[17] (HM448912).

Table S1. Genotypes and phenotypes of F_2 plants. M = MM♂ x Hicks♀; H = Hicks♂ x MM♀; f = flowering, nf = non-flowering

indivi-dual	genotype	pheno-type	leaf number	days until flowering	indivi-dual	genotype	pheno-type	leaf number	days until flowering
**M001**	NtFT5/NtFT5$_{MM}$	f	23	56	**H002**	NtFT5/NtFT5$_{MM}$	f	24	53
**M004**	NtFT5/NtFT5$_{MM}$	f	20	56	**H005**	NtFT5/NtFT5$_{MM}$	f	40	72
**M006**	NtFT5/NtFT5$_{MM}$	f	20	60	**H006**	NtFT5/NtFT5$_{MM}$	f	31	61
**M011**	NtFT5/NtFT5$_{MM}$	f	32	77	**H008**	NtFT5/NtFT5$_{MM}$	f	33	86
**M012**	NtFT5/NtFT5$_{MM}$	f	32	58	**H009**	NtFT5/NtFT5$_{MM}$	f	20	59
**M013**	NtFT5/NtFT5$_{MM}$	f	33	67	**H010**	NtFT5/NtFT5$_{MM}$	f	20	50
**M014**	NtFT5/NtFT5$_{MM}$	f	21	56	**H013**	NtFT5/NtFT5$_{MM}$	f	22	54
**M016**	NtFT5/NtFT5$_{MM}$	f	17	61	**H014**	NtFT5/NtFT5$_{MM}$	f	21	50
**M017**	NtFT5/NtFT5$_{MM}$	f	32	67	**H016**	NtFT5/NtFT5$_{MM}$	f	26	56
**M018**	NtFT5/NtFT5$_{MM}$	f	24	56	**H018**	NtFT5/NtFT5$_{MM}$	f	33	59
**M021**	NtFT5/NtFT5$_{MM}$	f	31	61	**H022**	NtFT5/NtFT5$_{MM}$	f	23	52
**M024**	NtFT5/NtFT5$_{MM}$	f	26	48	**H023**	NtFT5/NtFT5$_{MM}$	f	35	77
**M026**	NtFT5/NtFT5$_{MM}$	f	28	50	**H026**	NtFT5/NtFT5$_{MM}$	f	31	71
**M027**	NtFT5/NtFT5$_{MM}$	f	45	95	**H027**	NtFT5/NtFT5$_{MM}$	f	24	57
**M028**	NtFT5/NtFT5$_{MM}$	f	23	57	**H028**	NtFT5/NtFT5$_{MM}$	f	22	51
**M034**	NtFT5/NtFT5$_{MM}$	f	23	50	**H030**	NtFT5/NtFT5$_{MM}$	f	50	90
**M037**	NtFT5/NtFT5$_{MM}$	f	20	49	**H031**	NtFT5/NtFT5$_{MM}$	f	29	57
**M039**	NtFT5/NtFT5$_{MM}$	f	24	55	**H032**	NtFT5/NtFT5$_{MM}$	f	26	51
**M040**	NtFT5/NtFT5$_{MM}$	f	28	57	**H034**	NtFT5/NtFT5$_{MM}$	f	20	50
**M043**	NtFT5/NtFT5$_{MM}$	f	20	46	**H035**	NtFT5/NtFT5$_{MM}$	f	23	56
**M044**	NtFT5/NtFT5$_{MM}$	f	24	56	**H037**	NtFT5/NtFT5$_{MM}$	f	22	60
**M045**	NtFT5/NtFT5$_{MM}$	f	44	105	**H038**	NtFT5/NtFT5$_{MM}$	f	55	90
**M051**	NtFT5/NtFT5$_{MM}$	f	19	62	**H039**	NtFT5/NtFT5$_{MM}$	f	24	59
**M055**	NtFT5/NtFT5$_{MM}$	f	25	53	**H040**	NtFT5/NtFT5$_{MM}$	f	24	53
**M056**	NtFT5/NtFT5$_{MM}$	f	24	55	**H042**	NtFT5/NtFT5$_{MM}$	f	27	48
**M057**	NtFT5/NtFT5$_{MM}$	f	27	53	**H043**	NtFT5/NtFT5$_{MM}$	f	28	60
**M058**	NtFT5/NtFT5$_{MM}$	f	42	105	**H044**	NtFT5/NtFT5$_{MM}$	f	24	56
**M060**	NtFT5/NtFT5$_{MM}$	f	37	73	**H045**	NtFT5/NtFT5$_{MM}$	f	23	56
**M061**	NtFT5/NtFT5$_{MM}$	f	21	48	**H046**	NtFT5/NtFT5$_{MM}$	f	26	57
**M066**	NtFT5/NtFT5$_{MM}$	f	23	53	**H048**	NtFT5/NtFT5$_{MM}$	f	20	48
**M067**	NtFT5/NtFT5$_{MM}$	f	25	56	**H049**	NtFT5/NtFT5$_{MM}$	f	26	56
**M068**	NtFT5/NtFT5$_{MM}$	f	21	53	**H058**	NtFT5/NtFT5$_{MM}$	f	21	48
**M070**	NtFT5/NtFT5$_{MM}$	f	24	53	**H059**	NtFT5/NtFT5$_{MM}$	f	24	56
**M073**	NtFT5/NtFT5$_{MM}$	f	23	49	**H060**	NtFT5/NtFT5$_{MM}$	f	27	53
**M074**	NtFT5/NtFT5$_{MM}$	f	25	56	**H061**	NtFT5/NtFT5$_{MM}$	f	25	60
**M075**	NtFT5/NtFT5$_{MM}$	f	25	57	**H063**	NtFT5/NtFT5$_{MM}$	f	20	47
**M076**	NtFT5/NtFT5$_{MM}$	f	24	73	**H064**	NtFT5/NtFT5$_{MM}$	f	24	56
**M079**	NtFT5/NtFT5$_{MM}$	f	26	56	**H065**	NtFT5/NtFT5$_{MM}$	f	22	53
**M080**	NtFT5/NtFT5$_{MM}$	f	28	78	**H066**	NtFT5/NtFT5$_{MM}$	f	24	60
**M082**	NtFT5/NtFT5$_{MM}$	f	21	50	**H067**	NtFT5/NtFT5$_{MM}$	f	24	51
**M083**	NtFT5/NtFT5$_{MM}$	f	26	53	**H068**	NtFT5/NtFT5$_{MM}$	f	23	56
**M086**	NtFT5/NtFT5$_{MM}$	f	20	53	**H069**	NtFT5/NtFT5$_{MM}$	f	22	48
**M088**	NtFT5/NtFT5$_{MM}$	f	27	53	**H072**	NtFT5/NtFT5$_{MM}$	f	26	56
**M090**	NtFT5/NtFT5$_{MM}$	f	24	56	**H073**	NtFT5/NtFT5$_{MM}$	f	24	56
**M094**	NtFT5/NtFT5$_{MM}$	f	44	86	**H075**	NtFT5/NtFT5$_{MM}$	f	26	62
**M095**	NtFT5/NtFT5$_{MM}$	f	25	56	**H076**	NtFT5/NtFT5$_{MM}$	f	23	61
**M097**	NtFT5/NtFT5$_{MM}$	f	26	53	**H077**	NtFT5/NtFT5$_{MM}$	f	19	50
**M098**	NtFT5/NtFT5$_{MM}$	f	40	86	**H079**	NtFT5/NtFT5$_{MM}$	f	26	52
**M099**	NtFT5/NtFT5$_{MM}$	f	23	50	**H080**	NtFT5/NtFT5$_{MM}$	f	28	51
**M100**	NtFT5/NtFT5$_{MM}$	f	25	55	**H081**	NtFT5/NtFT5$_{MM}$	f	30	63
**M002**	NtFT5$_{MM}$	nf	70		**H082**	NtFT5/NtFT5$_{MM}$	f	21	47
**M009**	NtFT5$_{MM}$	nf	63		**H083**	NtFT5/NtFT5$_{MM}$	f	21	52
**M015**	NtFT5$_{MM}$	nf	63		**H084**	NtFT5/NtFT5$_{MM}$	f	22	56
**M019**	NtFT5$_{MM}$	nf	65		**H085**	NtFT5/NtFT5$_{MM}$	f	23	56
**M020**	NtFT5$_{MM}$	nf	49		**H086**	NtFT5/NtFT5$_{MM}$	f	33	71
**M031**	NtFT5$_{MM}$	nf	43		**H088**	NtFT5/NtFT5$_{MM}$	f	26	56
**M035**	NtFT5$_{MM}$	nf	37		**H089**	NtFT5/NtFT5$_{MM}$	f	23	49
**M036**	NtFT5$_{MM}$	nf	49		**H090**	NtFT5/NtFT5$_{MM}$	f	24	50

**M041**	$NtFT5_{MM}$	nf	37		**H091**	$NtFT5/NtFT5_{MM}$	f	44	108
**M047**	$NtFT5_{MM}$	nf	45		**H094**	$NtFT5/NtFT5_{MM}$	f	25	55
**M048**	$NtFT5_{MM}$	nf	64		**H096**	$NtFT5/NtFT5_{MM}$	f	18	47
**M053**	$NtFT5_{MM}$	nf	36		**H097**	$NtFT5/NtFT5_{MM}$	f	23	48
**M077**	$NtFT5_{MM}$	nf	47		**H011**	$NtFT5_{MM}$	nf	59	
**M078**	$NtFT5_{MM}$	nf	44		**H020**	$NtFT5_{MM}$	nf	42	
**M081**	$NtFT5_{MM}$	nf	52		**H024**	$NtFT5_{MM}$	nf	58	
**M084**	$NtFT5_{MM}$	nf	54		**H029**	$NtFT5_{MM}$	nf	66	
**M085**	$NtFT5_{MM}$	nf	46		**H036**	$NtFT5_{MM}$	nf	48	
**M087**	$NtFT5_{MM}$	nf	nd		**H052**	$NtFT5_{MM}$	nf	58	
**M096**	$NtFT5_{MM}$	nf	70		**H053**	$NtFT5_{MM}$	nf	52	
**M005**	NtFT5	f	19	53	**H071**	$NtFT5_{MM}$	nf	64	
**M007**	NtFT5	f	22	50	**H074**	$NtFT5_{MM}$	nf	37	
**M010**	NtFT5	f	23	50	**H078**	$NtFT5_{MM}$	nf	48	
**M023**	NtFT5	f	24	62	**H087**	$NtFT5_{MM}$	nf	36	
**M025**	NtFT5	f	23	52	**H092**	$NtFT5_{MM}$	nf	40	
**M029**	NtFT5	f	21	50	**H093**	$NtFT5_{MM}$	nf	50	
**M030**	NtFT5	f	22	47	**H095**	$NtFT5_{MM}$	nf	66	
**M033**	NtFT5	f	24	49	**H098**	$NtFT5_{MM}$	nf	36	
**M038**	NtFT5	f	38	86	**H099**	$NtFT5_{MM}$	nf	53	
**M042**	NtFT5	f	20	46	**H100**	$NtFT5_{MM}$	nf	42	
**M046**	NtFT5	f	19	48	**H001**	NtFT5	f	23	50
**M049**	NtFT5	f	22	49	**H003**	NtFT5	f	21	48
**M050**	NtFT5	f	21	49	**H004**	NtFT5	f	21	53
**M059**	NtFT5	f	23	50	**H007**	NtFT5	f	20	53
**M062**	NtFT5	f	26	42	**H012**	NtFT5	f	22	50
**M063**	NtFT5	f	24	47	**H017**	NtFT5	f	24	51
**M064**	NtFT5	f	21	47	**H019**	NtFT5	f	23	49
**M065**	NtFT5	f	23	53	**H021**	NtFT5	f	29	48
**M069**	NtFT5	f	20	60	**H025**	NtFT5	f	23	47
**M071**	NtFT5	f	21	49	**H033**	NtFT5	f	21	48
**M072**	NtFT5	f	23	49	**H050**	NtFT5	f	21	51
**M089**	NtFT5	f	21	50	**H051**	NtFT5	f	22	48
**M091**	NtFT5	f	19	63	**H054**	NtFT5	f	24	57
**M092**	NtFT5	f	22	48	**H056**	NtFT5	f	26	63
**M093**	NtFT5	f	23	49	**H057**	NtFT5	f	22	49
					**H062**	NtFT5	f	23	52
					**H070**	NtFT5	f	21	48
**MM**					**Hicks**				
**1**	$NtFT5_{MM}$	nf	53		**1**	NtFT5	f	21	46
**2**	$NtFT5_{MM}$	nf	62		**2**	NtFT5	f	20	46
**3**	$NtFT5_{MM}$	nf	59		**3**	NtFT5	f	20	46
**4**	$NtFT5_{MM}$	nf	50		**4**	NtFT5	f	20	46
**5**	$NtFT5_{MM}$	nf	52		**5**	NtFT5	f	18	46
**6**	$NtFT5_{MM}$	nf	55		**6**	NtFT5	f	21	46
**7**	$NtFT5_{MM}$	nf	52		**7**	NtFT5	f	20	46
**8**	$NtFT5_{MM}$	nf	53		**8**	NtFT5	f	19	46
**9**	$NtFT5_{MM}$	nf	54		**9**	NtFT5	f	20	46
					**10**	NtFT5	f	21	46

Table S2. List of the oligonucleotides used in this study. Underlined sequences are the overhangs containing the restriction sites used for cloning (lower case letters).

Purpose	Name	5'→3' sequence	T_m
pRT104/ pENTR4/ pTRBO cloning	*NtFT5* for XhoI	AGActcgagATGCCAAGAGAACGTGAAC	55 °C
	NtFT5 rev XbaI	AGAtctagaTCAATCGGCAGACCTTC	
	NtFT5$_{FN*}$ rev XbaI	ACAtctagaTTAATTAAAATAGACAGCAGCAAC	
	NtFT5$_{FNC*}$ rev XbaI	ACAtctagaTAAACAATTAAAATAGACAGCAGC	
	NtFT5$_{R61G}$ for	GAGATGACCTTgGTACCTTTTACAC	
	NtFT5$_{R61G}$ rev	GTGTAAAAGGTACcAAGGTCATCTC	
	NtFT5$_{R129A}$ for	AGACAATTGGGTgctCAGACAGTG	
	NtFT5$_{R129A}$ rev	CACTGTCTGagcACCCAATTGTCT	
	NtFT5$_{MM\ W170G}$ rev XbaI	ACAtctagaCTACGTtccCCACTGCCTCTCT	
	NtFT5$_{G170W}$ rev XbaI	ACAtctagaTCAATCGGCAGACCTTCTACGTCCccaACTG	
	NtFT5 for PacI	AGAttaattaaATGCCAAGAGAACGTGAAC	
	NtFT5 rev NotI	AGAgcggccgcTTAATCGGCAGACCTTCTA	
	NtFT5$_{MM}$ rev NotI	AGAgcggccgcTCAATCGGCAGACCTTC	
	AtFT for SalI	AGAgtcgacATGTCTATAAATATAAGAGACC	
	AtFT rev NotI	AGAgcggccgcCTAAAGTCTTCTTCCTCCGCAG	
P-NtFT5$_{2.6kb}$	P-NtFT5$_{2.6kb}$ for XmaI	AGAgggcccGTCCGTACCAACTTCTTAGTTC	57°C
	P-NtFT5$_{2.6kb}$ rev XhoI	AGActcgagGTTGATGCTTAAATAAATAAAACTAAC	
qRT-PCR expression analysis	qRT *NtFT4* for	GATATCCCAGCAACTACAGATACAAG	67 °C
	qRT *NtFT4* rev	GAAACGGGCAAACCAAGATTGTAAAC	
	qRT *NtFT5* for	TCCCAAGTTATTAACCAGCCC	67 °C
	qRT *NtFT5* rev	CATAGCACACAATTTCCTGGC	
	qRT *NtEF-1a* for	AAGCTGACTGTGCTGTCCTGA	66.7 °C
	qRT *NtEF-1a* rev	GGTGGTAGCATCCATCTTGTTG	
Fragment length analysis	NtFT5 ExIV 396bp for	AATTGGGTCGACAGACAGTG	
	NtFT5 Stop rev	TCAATCGGCAGACCTTCTAC	
mcBiFC	GGGS Linker NtFT5 for	GGTGGCGGTTCTGGCGGAGGTTCTGGTGGTGGcTCCATGCCAAGAGAACGTG	
	NtFT5 BsrGI rev	ACAtgtacaTCAATCGGCAGACCTTC	
	SCYCE_for_Sal	aagagtcgacATGGACAAGCAGAAGAAC	
	HA_rev_Linker_ BamHI	aagaGGATCCaccaccagaacctccgccagaaccgccaccAGCGTAATCTGGAAC	

Supplementary References

1. Gao, H. et al. Florigen activation complex forms via multifaceted assembly in Arabidopsis. *Nature* **648**, 686–695 (2025).
2. Kardailsky, I. et al. Activation tagging of the floral inducer FT. *Science* **286**, 1962–1965 (1999).
3. Kobayashi, Y., Kaya, H., Goto, K., Iwabuchi, M. & Araki, T. A pair of related genes with antagonistic roles in mediating flowering signals. *Science* **286**, 1960–1962 (1999).
4. Harig, L. et al. Proteins from the FLOWERING LOCUS T-like subclade of the PEBP family act antagonistically to regulate floral initiation in tobacco. *Plant J.* **72**, 908–921 (2012).
5. Beinecke, F.A. et al. The FT/FD-dependent initiation of flowering under long-day conditions in the day-neutral species Nicotiana tabacum originates from the facultative short-day ancestor Nicotiana tomentosiformis. *Plant J.* **96**, 329–342 (2018).
6. Amaya, I., Ratcliffe, O.J., & Bradley, D. J. Expression of CENTRORADIALIS (CEN) and CEN-like genes in tobacco reveals a conserved mechanism controlling phase change in diverse species. *Plant Cell* **11**, 1405–1418 (1999).
7. Navarro, C. et al. Control of flowering and storage organ formation in potato by FLOWERING LOCUS T. *Nature* **478**, 119–122 (2011).
8. Guo, J-L. et al. Cloning and characterization of a potato *TFL1* gene involved in tuberization regulation. *Plant Cell Tiss Organ Cult* **103**, 103–109 (2010).
9. Lifschitz, E. et al. The tomato FT ortholog triggers systemic signals that regulate growth and flowering and substitute for diverse environmental stimuli. *Proc. Natl Acad. Sci. USA* **103**, 6398–6403 (2006).
10. Kojima, S. et al. Hd3a, a rice ortholog of the Arabidopsis FT gene, promotes transition to flowering downstream of Hd1 under short-day conditions. *Plant Cell Physiol*. **43**, 1096–1105 (2002).
11. Nakagawa, M., Shimamoto, K., & Kyozuka, J. Overexpression *of RCN1* and *RCN2,* rice *TERMINAL FLOWER 1/CENTRORADIALIS* homologs, confers delay of phase. *Plant J*. **29**, 743–750 (2002).
12. Kong, F. et al. Two coordinately regulated homologs of FLOWERING LOCUS T are involved in the control of photoperiodic flowering in soybean. *Plant Physiol*. **154**, 1220–1231 (2010).
13. Tian, Z. et al. Artificial selection for determinate growth habit in soybean. *Proc. Natl Acad. Sci. USA* **107**, 8563–8568 (2010).
14. Laurie, R.E. et al. The Medicago FLOWERING LOCUS T homolog, MtFTa1, is a key regulator of flowering time. *Plant Physiol*. **156**, 2207–2224 (2011).
15. Hecht, V. et al. The pea *GIGAS* gene is a *FLOWERING LOCUS T* homolog necessary for graft-transmissible specification of flowering but not for responsiveness to photoperiod. *Plant Cell* **23**: 147–161 (2011).
16. Foucher, F. et al. DETERMINATE and LATE FLOWERING are two TERMINAL FLOWER1/CENTRORADIALIS homologs that control two distinct phases of flowering initiation and development in pea. *Plant Cell* **15**, 2742–2754 (2003).
17. Pin, P.A. et al. An antagonistic Pair of *FT* homologs mediates the control of flowering time in sugar beet. *Science* **330**, 1397–1400 (2010).
