# Supplementary material for: A mutant allele of the long-day floral activator NtFT5 is associated with the short-day-specific flowering of tobacco cultivar Maryland Mammoth
Source: Commun Biol. 2026 Aug 26;9:1144. doi: 10.1038/s42003-026-10729-4 (PMC13507162; doi:10.1038/s42003-026-10729-4)
Supplement: Supplementary file 3 [file 42003_2026_10729_MOESM3_ESM.pdf]

Description of Additional Supplementary Data 1

File Name: Supplementary Data 1
Description: The source data (relative gene expression levels, correlation coefficients, leaf number at flowering, days until flowering, genotypes and phenotypes of F2 plants, and mcBiFC signal data) corresponding to the graphs and charts presented in Figure 1-6 and Supplementary Figures S1–S8.
